# Serial Ketamine Infusions as Adjunctive Therapy to Inpatient Care for Depression: The KARMA-Dep 2 Randomized Clinical Trial

**DOI:** 10.1001/jamapsychiatry.2025.3019

**Published:** 2025-10-22

**Authors:** Ana Jelovac, Cathal McCaffrey, Masashi Terao, Enda Shanahan, Emma Whooley, Kelly McDonagh, Sarah McDonogh, Orlaith Loughran, Ellie Shackleton, Anna Igoe, Sarah Thompson, Enas Mohamed, Duyen Nguyen, Ciaran O’Neill, Cathal Walsh, Declan M. McLoughlin

**Affiliations:** 1 Department of Psychiatry, School of Medicine, Trinity College Dublin, St Patrick’s University Hospital, Dublin, Ireland; 2 Centre for Public Health, Global Research Institute for Health Sciences, Queen’s University Belfast, Belfast, United Kingdom; 3 Department of Public Health and Primary Care, Trinity College Dublin, Dublin, Ireland; 4 Trinity College Institute of Neuroscience, Trinity College Dublin, Dublin, Ireland

## Abstract

**Importance:**

Serial ketamine infusions are being increasingly adopted as off-label treatment for major depression in routine clinical practice, yet robust psychoactive placebo-controlled trial evidence for short- and long-term efficacy and safety remains limited.

**Objective:**

To assess antidepressant efficacy, safety, tolerability, cost-effectiveness, and quality of life during and after serial ketamine infusions compared with midazolam as an adjunct to usual inpatient care.

**Design, Setting, and Participants:**

The KARMA-Dep 2 trial was an investigator-led, double-blind, randomized, midazolam-controlled, pragmatic trial conducted at an academic center in Ireland between September 2021 and August 2024. Participants included adults (≥18 years) hospitalized with a *DSM-5* major depressive episode (unipolar or bipolar) and baseline Montgomery-Åsberg Depression Rating Scale (MADRS) score ≥20.

**Interventions:**

Participants were randomized 1:1 to receive up to 8 twice-weekly intravenous infusions of either ketamine (0.5 mg/kg) or midazolam (0.045 mg/kg) as an adjunct to usual-care pharmacotherapy and other aspects of routine inpatient psychiatric care. Participants were followed up for 6 months.

**Main Outcomes and Measures:**

The primary outcome was change in depression symptom severity measured by the observer-rated MADRS score from baseline to end of treatment. Secondary outcomes included self-reported depression severity, safety, tolerability, health care costs, and quality of life.

**Results:**

Of 65 randomized participants (mean [SD] age, 53.5 [18.6] years; 37 [59.7%] male), 62 were included in the final analysis. In the analysis of primary outcome, end-of-treatment MADRS scores did not significantly differ between the ketamine and midazolam groups (adjusted mean difference, −3.16 points, 95% CI, −8.54 to 2.22; *P* = .25; Cohen *d*, −0.29). Similarly, there was no significant between-group difference between Quick Inventory of Depressive Symptoms, Self-Report, scores (adjusted mean difference, −0.002; 95% CI, −2.71 to 2.71; *P* > .99; Cohen *d*, −0.0004). There were no significant between-group differences on other secondary outcomes, including cognition, cost-effectiveness, or quality of life. Most patients and raters accurately guessed treatment allocation.

**Conclusions and Relevance:**

Serial adjunctive ketamine infusions were not more effective than serial midazolam infusions in reducing depressive symptoms in inpatients receiving usual psychiatric care.

**Trial Registration:**

ClinicalTrials.gov Identifier: NCT04939649

## Key Points

**Question:**

For inpatients with moderate to severe depression, is repeated intravenous ketamine superior to a psychoactive placebo (midazolam) in reducing depressive symptoms?

**Findings:**

In this randomized clinical trial, there was no statistically significant difference in the primary outcome, end-of-treatment Montgomery-Åsberg Depression Rating Scale score, between the ketamine and midazolam groups. No significant differences were observed on secondary efficacy, cognitive, economic, or quality-of-life outcomes.

**Meaning:**

These findings do not support a superior antidepressant effect for serial intravenous ketamine infusions as an adjunctive therapy to usual inpatient care for moderate to severe depression.

## Teaser

This randomized clinical trial evaluates outcomes following adjunctive ketamine infusions vs midazolam for depression.

## Introduction

As many as one-third of patients with major depression do not achieve remission with oral antidepressants.^1^ Although single ketamine infusions produce rapid antidepressant effects, these benefits typically dissipate within days. Esketamine nasal spray has received regulatory approval in multiple jurisdictions, yet off-label intravenous racemic ketamine use is widespread and increasing,^2^ with some evidence suggesting that it may be more effective than esketamine.^3^ In 2022, 28.3% of patients receiving ketamine in the US had a billing diagnosis of depression.^4^

A key challenge in evaluating ketamine’s efficacy is the difficulty in maintaining blinding due to its distinctive dissociative effects. Meta-analytic evidence shows substantially larger effect sizes in saline-controlled trials (Cohen *d*, 1.8) compared to those using midazolam as a psychoactive placebo (Cohen *d*, 0.7).^5^ While a number of saline-controlled^6,7,8^ or single-infusion ketamine vs midazolam comparisons^9,10,11,12^ are available, the evidence from midazolam-controlled trials of serial adjunctive ketamine infusions, reflecting contemporary real-world clinical practice,^13^ remains sparse.^14,15,16^ In the largest such trial to date, Shiroma et al^14^ randomized 54 outpatients with depression to receive 6 thrice-weekly infusions of ketamine or midazolam (with midazolam participants crossing over to ketamine after 5 infusions) and found no significant difference in Montgomery-Åsberg Depression Rating Scale (MADRS) scores 24 hours after the final infusion. However, there was a significant advantage for ketamine after 5 infusions, before the midazolam arm crossover. In a pilot trial, Gallagher et al^15^ found no difference in 24-item Hamilton Depression Rating Scale outcomes between 4 once-weekly ketamine or midazolam infusions in 25 inpatients at end-of-treatment assessment. Similarly, a pilot trial by Pattanaseri et al^16^ randomized 20 patients with depression to receive ketamine or midazolam infusions over 3 consecutive days, reporting no significant between-group differences in MADRS scores after the final infusion.

The present randomized, double-blind, psychoactive placebo-controlled trial was designed to evaluate the efficacy of serial adjunctive ketamine infusions combined with usual inpatient care in adults hospitalized with moderate to severe depression. We hypothesized that repeated ketamine infusions would improve mood outcomes compared to midazolam. Secondary objectives included evaluating the impact of ketamine infusions on safety, tolerability, health care costs, and quality of life.

## Methods

### Study Design and Setting

Ketamine as an Adjunctive Therapy for Major Depression (2) (KARMA-Dep [2]) was an investigator-led, pragmatic, randomized, midazolam-controlled, patient- and rater-blind, parallel-group superiority trial of up to 8 serial adjunctive intravenous ketamine or midazolam infusions with a 24-week naturalistic follow-up. The trial was conducted between September 13, 2021, and August 12, 2024, at an academic center: St Patrick’s University Hospital in Dublin, Ireland. The study received ethical approval from the Mater Misericordiae Institutional Review Board and the site’s research ethics committee. Protocol amendments were approved by the National Research Ethics Committee. The protocol,^17^ including all amendments, is available in Supplement 1. The trial was registered prior to participant enrollment on EudraCT (2019-003109-92) and ClinicalTrials.gov (NCT04939649). The statistical analysis plan, finalized before database lock, is available online (https://osf.io/cjhnd) and in Supplement 1. The trial was funded by a governmental agency (Health Research Board) and had an academic sponsor (Trinity College Dublin). The hospital’s service user group assisted with study design, and a service user representative was a member of the Trial Steering Committee. This study followed the Consolidated Standards of Reporting Trials (CONSORT) reporting guideline.

### Eligibility Criteria

**Figure 1.**
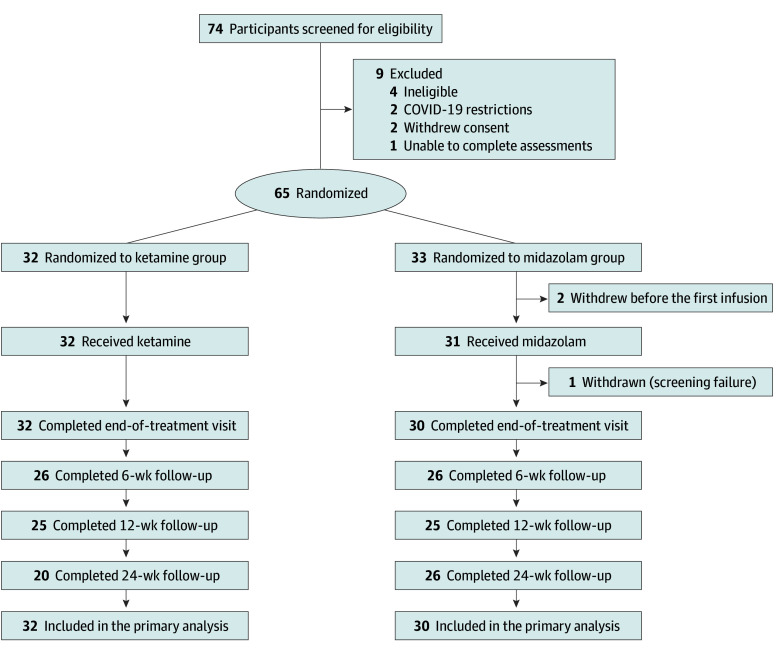
CONSORT Flow Diagram

Electronic medical records of all inpatient admissions (n = 3517) were prescreened by study physicians. Potentially eligible patients (n = 371) were invited to participate in the screening visit and, if agreeable (n = 74), provided written informed consent for screening to confirm eligibility (Figure 1). The trial enrolled adult (age ≥18 years) voluntary inpatients with unipolar or bipolar depression meeting *DSM-5* criteria for a major depressive episode confirmed by the Mini-International Neuropsychiatric Interview^18^ and scoring 20 or higher on the MADRS^19^ at screening visit and 40 minutes before the first infusion. Exclusion criteria are described in the Methods in Supplement 2.

### Randomization and Allocation Concealment

Eligible consenting participants were randomly assigned in a 1:1 ratio using randomly permuted blocks of 4, 6, and 8. Randomization was carried out independently by a statistician at the Centre for Support and Training in Analysis and Research, University College Dublin, not otherwise involved in the trial. Allocation information was stored in sequentially numbered opaque sealed envelopes in a secure location accessible to the trial anesthesiologist. To ensure patient safety, the trial anesthesiologist administering the infusions and trial pharmacists were not blinded but were not involved in recruitment, outcome assessments, or data analysis. Study physicians enrolling patients, patients, health care professionals, outcome assessors, investigators, and the trial statistician were blind to treatment allocation.

### Interventions

Ketamine (0.5 mg/kg of body weight) and midazolam (0.045 mg/kg) were administered intravenously as colorless saline solutions over a 40-minute period by an attending anesthesiologist who was present throughout infusions and postinfusion observation periods. Midazolam was used as an active comparator to help maintain blinding due to its acute sedative effects and similar half-life to ketamine. To reflect real-world clinical practice and enhance generalizability, patients continued to receive usual care, including concomitant pharmacotherapy, nursing care, psychotherapies, occupational therapy, psychoeducation programs, and other nonpharmacological interventions during the randomized treatment phase of the trial and the naturalistic follow-up phase.

Vital signs (heart rate, pulse oximetry, and blood pressure) were monitored at each infusion before, during, and up to 120 minutes after the start of infusions. Continuous electrocardiogram monitoring was performed throughout infusion observation periods. Infusions were discontinued early if nonphysiological hemodynamic changes occurred, operationalized as heart rate greater than 110 bpm or systolic/diastolic blood pressure greater than 180/100 persisting for more than 15 minutes and unresponsive to β-blocker therapy. Participants were discontinued from the interventional phase of the trial by the investigators if an infusion was discontinued for the above hemodynamic changes, if other serious medical contraindications occurred, or if patients developed acute psychiatric worsening (mania, psychosis, or becoming severely depressed or suicidal). Reflecting the pragmatic nature of the trial, treatment could also end earlier than 8 infusions, based on clinical judgment of the treating psychiatrists blind to treatment allocation. Patients could also discontinue the randomized treatment early for any reason. In the event of earlier completion of the randomized treatment phase, an end-of-treatment visit was organized, and participants entered the 24-week naturalistic follow-up phase.

### Measures

The primary outcome was change in MADRS score (range 0-60, with higher scores indicating worse depression) from baseline (ie, 40 minutes before the first infusion) to end of treatment (ie, 24 hours after the final infusion). MADRS mood ratings were repeated 6, 12, and 24 weeks after the final infusion. MADRS interviews were performed by trained assessors (psychiatrists, masters-level psychology research assistants, and psychiatric nurses) using a structured interview guide.^20^ Training was carried out at the outset and repeated every 6 months using videotaped MADRS interviews with formal assessment of interrater reliability. The median intraclass correlation coefficient was 0.98 (range, 0.95-0.99).

Secondary efficacy outcomes were change in self-rated depressive symptoms from baseline to end of treatment on the 16-item Quick Inventory of Depressive Symptoms, Self-Report (QIDS-SR-16)^21^; response (ie, ≥50% improvement in MADRS score from baseline to end of treatment); remission (MADRS ≤10 at end of treatment); and relapse (evaluated in remitters and defined as MADRS ≥18 at any follow-up visit and/or psychiatric readmission or deliberate self-harm or suicide).

Before, during, and after each infusion, psychotomimetic and other adverse effects were monitored using the Clinician-Administered Dissociative States Scale (CADSS),^22^ item 1 (elevated mood) on the Young Mania Rating Scale (YMRS),^23^ positive symptom subscale of the Brief Psychiatric Rating Scale (BPRS),^24^ responsiveness subscale of the Observer’s Assessment of Alertness/Sedation Scale (OAA/S),^25^ Patient-Rated Inventory of Side Effects (PRISE),^26^ and 20-item Physician Withdrawal Checklist (PWC-20).^27^ Adverse events were recorded at each study visit and reported to the data monitoring committee and trial steering committee. Serious adverse events were also reported to the pharmacovigilance service at the Wellcome-Health Research Board Clinical Research Facility at St James’s Hospital, Dublin.

Adequacy of antidepressant trials during the current depressive episode was rated using the Antidepressant Treatment Response Questionnaire,^28^ based on review of medical records, clinical interview and, where necessary, dispensing pharmacy records. Global cognitive function was monitored before, during and after the infusion course using the Montreal Cognitive Assessment (MoCA),^29^ using alternate forms in a counterbalanced order to reduce practice and order effects. Health care costs were estimated using a version of the Client Service Receipt Inventory (CSRI).^30^ Health-related quality of life was measured using the 5-Level EuroQol 5-Dimensional Questionnaire (EQ-5D-5L).^31^

### Statistical Analysis

Based on a saline-controlled trial of serial ketamine infusions available when designing the present study,^8^ we estimated that 41 patients (52 assuming 20% dropout) per treatment group would be needed to have 90% power at 5% significance level to detect a difference of 8 or more points in MADRS score between the 2 groups. For reference, a between-group minimum clinically important difference on the MADRS is estimated at 7 to 9 points.^32^

The primary end point, for which there were no missing data, was analyzed using a general linear model with robust variance estimation to compare MADRS scores at the end of the trial between treatment arms, with baseline MADRS score as a covariate. The treatment effect is presented as adjusted mean difference in MADRS scores between groups with its corresponding 95% CI and a 2-sided *P* value (<.05 considered statistically significant). For secondary outcomes (QIDS-SR-16 and MoCA scores), the Benjamini-Hochberg procedure was applied to control the false discovery rate. For details of statistical and economic analyses, see Supplements 1 and 2. Analyses were conducted in Stata version 18 (StataCorp) and R version 4.5.0 (R Foundation).

## Results

### Study Participants

Of 65 randomized participants (mean [SD] age, 53.5 [18.6] years; 37 [59.7%] male and 25 [40.3%] female), 62 were included in the final analysis. Baseline characteristics of the final analysis set of participants (32 in the ketamine group and 30 in the midazolam group) are presented in Table 1. Participants received a median of 8 (range 1-8) infusions in the ketamine and 8 (1-8) in the midazolam group, all during inpatient admission.

**Table 1.** Demographic and Clinical Characteristics

Characteristic	Total sample (N = 62)	Ketamine group (n = 32)	Midazolam group (n = 30)
Age, y			
Mean (SD)	53.5 (18.6)	54.3 (18.2)	52.7 (19.2)
Range	20-89	20-84	21-89
Sex, No. (%)			
Male	37 (59.7)	19 (59.4)	18 (60.0)
Female	25 (40.3)	13 (40.6)	12 (40.0)
Race, No. (%)^a^			
White	62 (100)	32 (100)	30 (100)
Education, mean (SD), y	14.1 (3.4)	13.4 (2.9)	14.9 (3.8)
Employment status, No. (%)			
Employed	32 (51.6)	16 (50.0)	16 (53.3)
Other	30 (48.4)	16 (50.0)	14 (46.7)
Primary diagnosis, No. (%)			
Unipolar depression	56 (90.3)	28 (87.5)	28 (93.3)
Bipolar depression	6 (9.7)	4 (12.5)	2 (6.7)
Treatment-resistant depression (ATRQ criteria), No. (%)	30 (48.4)	16 (50.0)	14 (46.7)
Duration of episode, median (IQR), wk	20.5 (10.0-52.0)	18.5 (10.0-42.0)	22.5 (8.0-57.0)
Previous depressive episodes, median (IQR)	5.0 (2.0-11.0)	5.5 (2.5-12.5)	4.0 (2.0-8.0)
Age at onset, mean (SD), y	32.4 (16.8)	32.3 (16.1)	32.5 (17.7)
Body mass index, mean (SD)^b^	30.3 (4.8)	30.8 (4.2)	29.7 (5.3)
MADRS score at screening, mean (SD)	31.1 (5.8)	31.9 (6.7)	30.2 (4.6)
MADRS score at baseline, mean (SD)	28.8 (5.4)	29.8 (5.9)	27.7 (4.7)
QIDS-SR-16 score at screening, mean (SD)	15.3 (5.0)	15.9 (5.3)	14.6 (4.8)
QIDS-SR-16 score at baseline, mean (SD)	13.6 (4.6)	13.7 (4.5)	13.6 (4.7)
MoCA score at screening, mean (SD)	25.9 (3.2)	26.0 (3.0)	25.9 (3.5)
Baseline psychotropic medication, No. (%)			
Selective serotonin reuptake inhibitor	19 (30.6)	7 (21.9)	12 (40.0)
Serotonin-norepinephrine reuptake inhibitor	34 (54.8)	19 (59.4)	15 (50.0)
Tricyclic antidepressant	8 (12.9)	7 (21.9)	1 (3.3)
Mirtazapine	25 (40.3)	12 (37.5)	13 (43.3)
Vortioxetine	4 (6.5)	2 (6.3)	2 (6.7)
Atypical antipsychotic	30 (48.4)	16 (50.0)	14 (46.7)
Anticonvulsant mood stabilizer	11 (17.7)	6 (18.8)	5 (16.7)
Lithium	9 (14.5)	4 (12.5)	5 (16.7)
Benzodiazepine	21 (33.9)	13 (40.6)	8 (26.7)
Nonbenzodiazepine hypnotic	14 (22.6)	7 (21.9)	7 (23.3)

Abbreviations: ATRQ, Antidepressant Treatment Response Questionnaire; MADRS, Montgomery-Åsberg Depression Rating Scale; MoCA, Montreal Cognitive Assessment; QIDS-SR-16, 16-item Quick Inventory of Depressive Symptomatology–Self-Report.

^a^ Race data were collected via electronic health records and are reported for descriptive purposes.

^b^ Body mass index calculated as weight in kilograms divided by height in meters squared.

### Efficacy Outcomes

**Figure 2.**
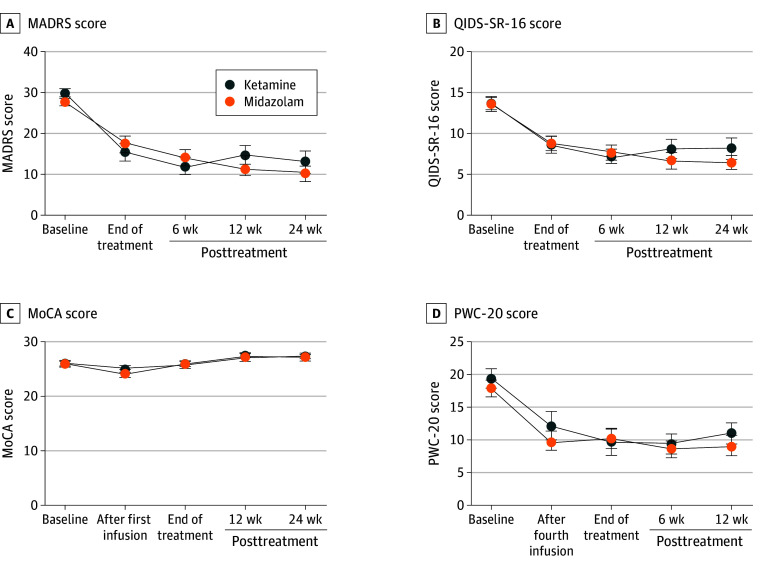
Clinical and Cognitive Outcomes During and After Serial Ketamine and Midazolam Infusions Sample sizes for each group at each time point are provided in eTable 5 in Supplement 2. Error bars represent standard errors. MADRS indicates Montgomery-Åsberg Depression Rating Scale; MoCA, Montreal Cognitive Assessment; PWC-20, 20-Item Physician Withdrawal Checklist; QIDS-SR-16, 16-Item Quick Inventory of Depressive Symptoms, Self-Report.

Adjusting for baseline score, there was no statistically significant difference in the primary outcome, end-of-treatment MADRS scores, favoring ketamine (n = 32) over midazolam (n = 30) (adjusted mean difference, −3.16; 95% CI, −8.54 to 2.22; *P* = .25; Cohen *d*, −0.29) (Figure 2A). Similarly, the analysis of secondary efficacy outcome, end-of-treatment QIDS-SR-16 scores, showed no significant treatment effect (−0.002; 95% CI, −2.71 to 2.71; *P* > .99; Cohen *d*, −0.0004) (Figure 2B), favoring ketamine (n = 31) over midazolam (n = 29). Intrainfusion MADRS and QIDS-SR-16 scores are presented in eFigure 1 in Supplement 2.

The response rate was 46.9% (15 of 32) in the ketamine group and 33.3% (10 of 30) in the midazolam group. Remission rates were 43.8% (14 of 32) and 30.0% (9 of 30), respectively. Among participants who achieved remission, 28.6% (4 of 14) in the ketamine group relapsed, while 44.4% (4 of 9) in the midazolam group relapsed during the 24-week follow-up.

### Safety and Tolerability Outcomes

**Figure 3.**
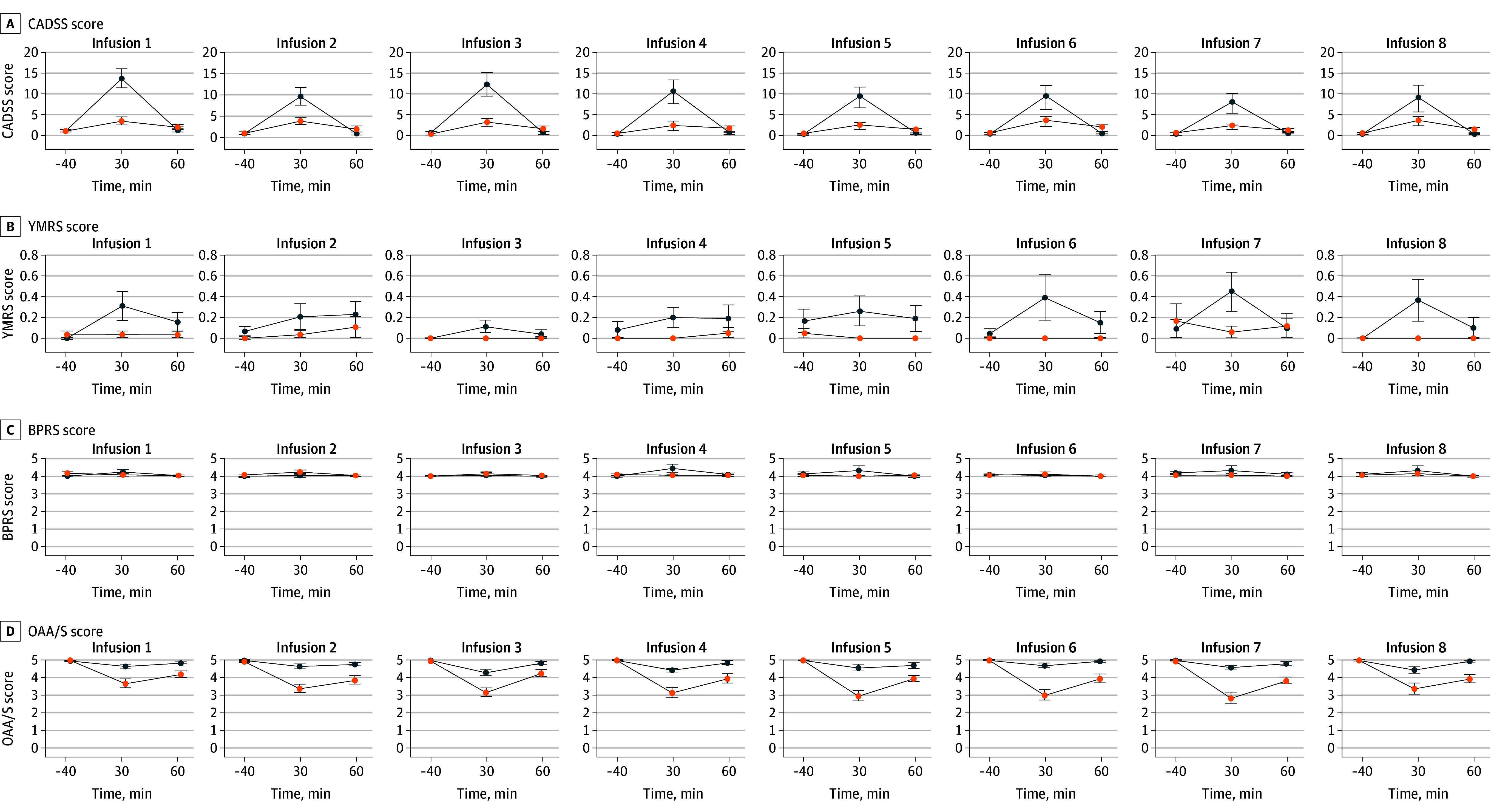
Dissociative Symptoms, Mania, Positive Symptoms, and Sedation During Ketamine and Midazolam Infusion Sessions Error bars represent standard errors. BPRS indicates Positive Symptom Subscale of the Brief Psychiatric Rating Scale; CADSS, Clinician-Administered Dissociative States Scale; OAA/S, Responsiveness Subscale of the Observer’s Assessment of Alertness/Sedation Scale; YMRS, Item 1 (Elevated Mood) from the Young Mania Rating Scale.

End-of-treatment cognitive outcome (MoCA) was similar between the ketamine (n = 28) and midazolam (n = 30) groups after controlling for baseline function (adjusted mean difference favoring midazolam, −0.22; 95% CI, −1.41 to 0.97; *P* > .99; Cohen *d*, −0.10) (Figure 2C). Scores on the PWC-20 scale, measuring possible withdrawal symptoms, decreased over the study course in both treatment groups (Figure 2D). During the infusion sessions, dissociative effects (CADSS) and mood elevation (YMRS) were increased in the ketamine group (Figure 3). A pronounced sedating effect was observed for midazolam 30 minutes after the start of infusions (OAA/S). In contrast, there was little difference between groups in positive symptoms as measured by the BPRS.

A summary of systematically collected side effects using the PRISE is provided in eTables 1 and 2 in Supplement 2. Overall, symptoms reported at screening tended to diminish in frequency and severity over the study period. Notably, urological symptoms did not increase over time in either group.

### Adverse Events

Spontaneously reported adverse events occurring in 10% or more of patients are presented in Table 2. The most common adverse events were fatigue, sleep disturbance, and abnormal liver function test results.

**Table 2.** Treatment-Emergent Adverse Events Occurring in ≥10% of Participants

Adverse event	No. (%)	
	Ketamine group (n = 32)	Midazolam group (n = 30)
Participants with ≥1 adverse event	29 (90.6)	28 (93.3)
Fatigue	12 (37.5)	13 (43.3)
Sleep disturbance	8 (25.0)	14 (46.7)
Abnormal liver function test	9 (28.1)	10 (33.3)
Dizziness	7 (21.9)	6 (20.0)
Headache	8 (25.0)	5 (16.7)
Bradycardia	3 (9.4)	7 (23.3)
Hypertension	7 (21.9)	3 (10.0)
Other arrhythmias	3 (9.4)	5 (16.7)
Hypotension	2 (6.2)	5 (16.7)
Nausea	6 (18.8)	0 (0.0)
Anxiety or panic attack	2 (6.2)	3 (10.0)
Depression	1 (3.1)	4 (13.3)

There were 7 serious adverse events in the ketamine group and 6 in the midazolam group. During the infusion phase in the ketamine group, 1 patient had worsening of depression, 2 developed elevated blood pressure unresponsive to β-blocker therapy resulting in termination of the infusion, 1 had severe nausea requiring treatment, and 1 had a vasovagal episode during cannulation. During the 24-week follow-up phase, 1 ketamine patient was discovered to have an aortocaval mass, and 1 required hospitalization due to a fall resulting in right ankle and left orbital fracture.

In the midazolam group during the treatment phase, 2 patients experienced a worsening of depression, 1 engaged in new-onset deliberate self-harm without suicidal intent, and 1 developed hypotension following an infusion. During the follow-up phase, 1 patient developed intermittent complete heart block and asymptomatic bradycardia 19 days after the last infusion, and 1 was hospitalized for a lower respiratory tract infection.

### Longer-Term Cost-Effectiveness and Quality of Life

Mean costs from baseline to 24-week follow-up were €6553.54 (US $7724.63) for ketamine (n = 32) and €6154.91 (US $7254.76) for midazolam (n = 30) (eTable 3 in Supplement 2). Observed mean improvements in MADRS were 16.30 for ketamine (n = 20) and 17.81 for midazolam (n = 26). Bootstrapping analysis of seemingly unrelated regression suggested the between-group differences in costs and outcomes were nonsignificant, with the incremental cost-effectiveness ratio estimated at −€570.39 (−US $672.21) per 1-point improvement in MADRS score. Thus, ketamine was less effective and more costly (*z* score, −0.01; *P* = .99) (eFigures 2 and 3 in Supplement 2). Analyses with imputed data for MADRS yielded similar findings (eFigures 4 and 5 in Supplement 2).

The observed increase in EQ-5D-5L score from screening visit to 24-week follow-up was 0.33 for ketamine (n = 19) and 0.31 for midazolam (n = 25). After multiple imputation, the increase was estimated at 0.36 for ketamine and 0.32 for midazolam. The quality-of-life analysis was conducted on the imputed data and showed no significant difference in change in EQ-5D-5L score from screening visit to 24-week follow-up (*t*_59_ = 0.45; *P* = .65).

### Blinding

To assess integrity of the blind, treatment guesses by raters and patients were analyzed after the first infusion, at end of treatment, and at the 24-week follow-up. Rater guesses were significantly associated with actual treatment assignment at all 3 time points (eTable 4 in Supplement 2). Raters correctly identified patients in the ketamine group at rates of 90.6% after the first infusion, 90.6% at end of treatment, and 85.0% at 24-week follow-up. Rater accuracy in identifying patients in the midazolam group was similarly high across the 3 time points (86.7%, 96.7%, and 92.3%, respectively).

While the association between patient guess and treatment assignment was not statistically significant after the first infusion, it became so by the end of treatment and remained so at the 24-week follow-up (eTable 4 in Supplement 2). Patients who received ketamine correctly identified their treatment at high rates at all 3 time points (78.1%, 78.1%, and 85.0%, respectively), whereas patients who received midazolam correctly guessed 46.4%, 60.0%, and 61.5% of the time.

## Discussion

In this randomized clinical trial, serial intravenous ketamine was not significantly more effective than serial midazolam in reducing depressive symptoms, and no significant benefit was observed on any secondary efficacy, cognitive, cost-effectiveness, or quality-of-life outcome. The interpretation of this result is inseparable from the study’s functional unblinding that occurred for raters from the outset and emerged for patients as the trial progressed. The observed nonsignificant treatment effect of 3.2 points on the MADRS may therefore be inflated by expectancy bias, highlighting the challenge of maintaining blinding in ketamine trials. Overall, the treatments appeared to be safe in the acute phase and 6-month follow-up.

Despite widespread clinical enthusiasm based on early reports of large rapid antidepressant effects of single ketamine infusions,^6,7^ our finding of a small between-group difference falls below both the effect sizes reported in prior open-label or saline-controlled studies, and the minimal clinically important difference for the MADRS. This underscores the need for a cautious interpretation of earlier, less rigorously controlled research. Our results are similar to a 2023 report^33^ finding no significant antidepressant effect from a single ketamine infusion when blinding was successfully maintained by general anesthesia. Our results are also in line with some previous smaller trials^15,16^ of serial ketamine using midazolam as a comparator, but differ from Shiroma et al^14^ who found an 8.3-point difference on the MADRS after 5 infusions. This divergence may be partly explained by differences in patient populations, with the Shiroma et al sample being outpatients from a Veterans Affairs facility. Our 43.8% remission rate in the ketamine arm is similar to the 46.3% rate reported by Ekstrand et al^34^ in a large open-label inpatient trial comparing serial ketamine infusions with electroconvulsive therapy (ECT) with a similar mean age (55 years) as our ketamine arm (54.3 years). Our remission rate exceeds the 32.3% rate observed by Anand et al^35^ in a large open-label study of predominantly outpatients (mean age, 45.6 years) where ketamine was compared with ultrabrief-pulse ECT. A 2025 meta-analysis pooling these well-powered trials with smaller studies found ketamine significantly less effective than ECT (Hedges *g*, –0.59; 95% CI, –1.01 to –0.17).^36^

### Strengths and Limitations

Unlike some prior ketamine research that relied on self-selected volunteers, the present trial enrolled consecutive inpatient admissions at an academic center, potentially reducing selection bias and moderating expectancy effects. However, the findings should be interpreted in the context of several limitations. First, although this is the largest midazolam-controlled trial of serial intravenous ketamine infusions to date, the planned sample size was not achieved due to COVID-19 pandemic–related logistical challenges (repurposing of a second planned recruitment site as a quarantine facility not accepting psychiatric admissions), which reduced statistical power. Second, while midazolam was selected to mitigate unblinding, this was not successful, raising the possibility that even the small observed effect reflects expectancy rather than a specific treatment effect. Third, the study was conducted in a resource-intensive inpatient setting with close continuous monitoring by 2 physicians, which increased costs and may limit the generalizability to more common outpatient or community-based practices.

## Conclusions

The KARMA-Dep 2 trial did not find evidence that serial ketamine infusions were superior to an active comparator for inpatients with moderate to severe depression. These results underscore the need for a cautious interpretation of saline-controlled studies and innovative trial designs that can effectively control for nonspecific effects inherent in ketamine trials in psychiatry.

## Notes

Supplement 1. Trial protocol and statistical analysis plan

Supplement 2. eMethods. Summary of exclusion criteria and cost-effectiveness statistical analysis eFigure 1. Montgomery-Åsberg Depression Rating Scale (MADRS) and 16-Item Quick Inventory of Depressive Symptoms, Self-Report (QIDS-SR-16) Outcomes Before, During and 24 Hours After Each Infusion in the Ketamine and Midazolam Groups eFigure 2. Cost-Effectiveness Plane with Seemingly Unrelated Regression Estimations (Using Observed Data) eFigure 3. Cost-Effectiveness Acceptability Curve Estimated from Seemingly Unrelated Regression (Using Observed Data) eFigure 4. Cost-Effectiveness Plane with Seemingly Unrelated Regression Estimations (Using Stacked Imputed Data) eFigure 5. Cost-Effectiveness Acceptability Curve Estimated from Seemingly Unrelated Regression (Using Stacked Imputed Data) eTable 1. Distress Levels for Symptom Groups from the Patient-Rated Inventory of Side Effects (PRISE) Across All Visits by Treatment Group eTable 2. Frequency of Individual Symptoms from the Patient-Rated Inventory of Side Effects (PRISE) Across All Visits by Treatment Group eTable 3. Unit Cost for Healthcare Services eTable 4. Assessment of Blinding After First Infusion, at the End-of-Treatment, and 24-Week Follow-Up eTable 5. Sample Sizes for Figure 2 Outcomes eReferences

Supplement 3. Data sharing statement
