# Supplementary material for: Serial Ketamine Infusions as Adjunctive Therapy to Inpatient Care for Depression: The KARMA-Dep 2 Randomized Clinical Trial
Source: JAMA Psychiatry. 2025 Oct 22;82(12):1216–24. doi: 10.1001/jamapsychiatry.2025.3019 (PMC12547681; doi:10.1001/jamapsychiatry.2025.3019)
Supplement: Supplement 1. — Trial protocol and statistical analysis plan [file jamapsychiatry-e253019-s001.pdf]

1 STUDY TITLE

Ketamine as an adjunctive therapy for Major Depression (2) - a randomised controlled trial: [KARMA-Dep (2)]

2 STUDY SPONSOR

The Provost, Fellows, Foundation Scholars and the other members of Board, of the College of the Holy and Undivided Trinity of Queen Elizabeth near Dublin (Trinity College Dublin)

3 APPLICATION DETAILS

<table>
<tr><td colspan="2">3.1 Study title
Ketamine as an adjunctive therapy for Major Depression (2) - a randomised controlled trial: [KARMA-Dep (2)]</td></tr>
<tr><td colspan="2">3.2 Reference numbers
Protocol identification (code or reference number): CRFSPN004
EudraCT number: 2019-003109-92
Date and version number: Version 6.1, 20/Oct/2023</td></tr>
<tr><td>3.3 Applicant details
<u>Chief investigator/ Co-ordinating investigator</u>
Name/title: Prof Declan McLoughlin, Research Professor of Psychiatry, Trinity College Dublin, & Consultant in Old Age Psychiatry, St Patrick's University Hospital, James Street, Dublin 8</td><td>Contact details: Dept of Psychiatry, Trinity College Dublin, St Patrick's University Hospital, James Street, Dublin 8
T: 01 2493385
E: d.mcloughlin@tcd.ie</td></tr>
<tr><td><u>Principal Investigators</u>
Name(s)/ titles: Professor Declan McLoughlin, Research Professor of Psychiatry, Trinity College Dublin</td><td>Contact details:
Dept of Psychiatry, St Patrick's University Hospital, James Street, Dublin 8
T: 01 2493385
E: d.mcloughlin@tcd.ie</td></tr>
<tr><td><u>Sponsor</u>
Name: The Provost, Fellows, Foundation Scholars and the other members of Board, of the College of the Holy and Undivided Trinity of Queen Elizabeth near Dublin otherwise known as Trinity College Dublin
College Green,
Dublin 2</td><td>Sponsor contact details:
Trinity College Dublin, Dublin 2
E: clinicaltrialsponsorship@tcd.ie</td></tr>
<tr><td><u>Funder:</u>
Name: Health Research Board</td><td>Health Research Board
Grattan House
67-72 Lower Mount Street
Dublin 2</td></tr>
</table>

3.4 Signatures

A review of the protocol has been completed and is understood and approved by the following:

SPONSOR'S REPRESENTATIVE
Dr Ruben E. Keane
Head of Clinical Sponsorship Oversight
Trinity College Dublin

The undersigned has read and understood the trial protocol and agrees to conduct the trial in compliance with the protocol and applicable regulations and guidelines.

PRINCIPAL INVESTIGATOR
Prof. Declan McLoughlin
Research Professor of Psychiatry

3.5 Other relevant information

The Wellcome-HRB Clinical Research Facility (CRF)
St James's Hospital
James Street
Dublin D08 A9788
T: 09 4103906
E: pharmacovigilance@tcd.ie

Trial Statistician
Dr Ricardo Segurado
Centre for Support and Training in Analysis and Research (CSTAR)
The School of Public Health, Physiotherapy and Population Science,
Woodview House,
University College Dublin,
Belfield,
Dublin 4
T: +353 (0)1 716 2076
F: +353 (0)1 716 3421
E: ricardo.segurado@ucd.ie

Health Economist
Professor Ciaran O'Neill
The Centre for Public Health
Global Research Institute for Health Sciences
Queens University Belfast
63 University Road
Belfast
6T7 1NF
Northern Ireland
T: +44 (0)28 9097 8928
E: Ciaran.ONeill@qub.ac.uk

Data Management Group
Data Management Centre
Health Research Board Clinical Research Facility
University of Galway
T: 091 494283
E: biometrics@nuigalway.ie

Sponsorship Project and Quality Manager
The Wellcome-HRB Clinical Research Facility (CRF)
St James's Hospital (www.sjhcrf.ie/)
James Street
Dublin D08 A9788
T: 09 4103906
E: clinicaltrialsponsorship@tcd.ie

4 CONFIDENTIALITY STATEMENT

This document contains confidential information that must not be disclosed to anyone other than the Sponsor, the investigative team, regulatory authorities, and members of the Research Ethics Committee.

5 TABLE OF CONTENTS

1 STUDY TITLE 1
2 STUDY SPONSOR 1
3 APPLICATION DETAILS 1
3.1 Study title 1
3.2 Reference numbers 1
3.3 Applicant details 1
3.4 Signatures 2
3.5 Other relevant information 3
4 CONFIDENTIALITY STATEMENT 4
5 TABLE OF CONTENTS 4
6 DOCUMENT HISTORY 6
7 SYNOPSIS 12
8 ABBREVIATIONS 15
9 INTRODUCTION 16
9.1 Background information 16
9.2 Rationale for the study 23
10 STUDY OBJECTIVE 25
10.1 Primary objective 25
10.2 Secondary objectives 25
10.3 Primary and secondary/exploratory endpoints/outcome measures 26
11 TRIAL DESIGN 26
11.1 General considerations 26
11.2 Selection of study population 27
11.3 Study assessments and procedures 30
11.4 Definition of end-of-trial 43
11.5 Discontinuation/withdrawal of patients from study protocol 44

12 TREATMENT OF TRIAL PATIENTS 45
12.1 Description of study treatment(s) 45
12.2 Formulation, packaging and handling 45
12.3 Storage and disposition of study treatment(s) 46
12.4 Accountability of the study treatment(s) 46
12.5 Assessment of compliance 47
12.6 Overdose of study treatment 47
12.7 Prior and concomitant therapy 48
13 SAFETY REPORTING 49
13.1 Definitions 49
13.2 Evaluation of AEs and SAEs 50
13.3 Reporting procedures for all adverse events 51
13.4 Reporting procedures for serious adverse events 51
13.5 Data Monitoring Committee (DMC) 53
13.6 Trial Steering Committee 53
13.7 Pregnancy 54
14 STATISTICS 54
14.1 Description of statistical methods 54
14.2 Determination of sample size 54
14.3 Demographic and baseline disease characteristics 55
14.4 Efficacy analysis 55
14.5 Safety analysis 55
14.6 Criteria for the termination of the trial 55
14.7 Procedure for accounting for missing, unused and spurious data 55
14.8 Procedure for reporting any deviation(s) from the original statistical plan 56
15 DIRECT ACCESS TO SOURCE DATA/DOCUMENTS 56
16 DATA HANDLING AND RECORD KEEPING 56
16.1 Data collection, source documents and case report forms (CRF) 56
16.2 Data reporting 57
17 RETENTION OF ESSENTIAL DOCUMENTS 57
18 QUALITY CONTROL AND QUALITY ASSURANCE PROCEDURES 58
19 MONITORING 58

20 AUDITS AND INSPECTIONS 58
21 ETHICS 59
21.1 Declaration of Helsinki 59
21.2 Good Clinical Practice 59
21.3 Approvals 59
21.4 Informed consent 59
21.5 Benefits and risks assessment 59
21.6 Patient confidentiality 60
21.7 Other ethical considerations 61
22 FINANCING AND INSURANCE/INDEMNITY 61
23 CLINICAL STUDY REPORT AND PUBLICATION POLICY 61
24 REFERENCES 62

6 DOCUMENT HISTORY

Document	Date of Issue	Summary of Change
Protocol version no. 1.0	30/Sep/19	Not applicable
Original protocol	30/Sep/19	Not applicable
Protocol version no 2.0	08/Nov/19	The primary objective was updated and clarified in the synopsis and section 10.1 to include the wording "and assess the mood-rating score difference between arms from before the first infusion to 24 hours after the final infusion, supplemented by a 95% confidence interval. There will also be a 24-week follow-up after the final infusion session."<br><br>The Exclusion criteria was updated in the synopsis and section 11.2.3 to include "Currently taking any of the contraindicated medications listed in section 12.7.2"<br><br>The period of clinical supervision was updated to be consistent throughout the protocol to include "from the beginning of the infusion" in sections 9.2.2, 11.3, 11.3.2.5, 11.3.4, 11.3.9 and section 11.5.

Protocol version no 2.1	6/Mar/20	Pharmacovigilance contact information has been updated throughout the protocol from CRF-UCC to SJH-CRF<br><br>Observer's Assessment of Alertness/Sedation Scale - Responsiveness Subscale included in assessments section 11.3 and 11.3.4
Protocol version no 3.0	02/Sep/20	• Correction of error to clarify that Concomitant medications two months prior to visit 0 will be documented<br><br>• Section 11.2.1 and 11.3.9 updated to include; "Where possible, patients taking any regular benzodiazepines (every day for the past five days) should omit their dose on the morning of infusion sessions (see section 12.7.1). It is appreciated that omission of benzodiazepines may not be possible for all patients. This will be as per the Investigators discretion and documented in the patient notes"<br><br>• Section 12.7.1 updated to include: "However where possible, patients taking any benzodiazepines should omit their dose on the morning of infusion sessions. As per the SmPc for Ketalar, Diazepam is known to increase the half-life of ketamine and prolongs its pharmacodynamic effects. Concurrent use of diazepam or other benzodiazepines will increase plasma levels and reduce the clearance rate of ketamine. However, benzodiazepines do not appear to interact with the antidepressant effect of ketamine (Shiroma, P. R., Thuras, P., Wels, J., Albott, C. S., Erbes, C., Tye, S., & Lim, K. O. (2020). A randomized, double-blind, active placebo-controlled study of efficacy, safety, and durability of repeated vs single subanesthetic ketamine for treatment-resistant depression. *Translational psychiatry*, *10*(1), 206. https://doi.org/10.1038/s41398-020-00897-0)."<br><br>• Section 11.3.1 and 11.3.10.2 updated to include sponsor e-mail address clinicaltrialsponsorship@tcd.ie

		• Table 1 Schedule of Assessments updated to include, fasting >6 hours before dosing and the omission of diazepines on the morning of dosing.<br><br>• Sections 9.2.2, 11.3.9 and 11.4 updated to include video conference instead of home visits.<br><br>• Section 13.2.2 updated to include Unlikely related: A clinical event, including laboratory test abnormality, with a temporal relationship to drug administration which makes a causal relationship improbable, and in which other drugs, chemicals or underlying disease provide plausible explanations.
Protocol Version 4.2	03/Aug/2022	• Sections 7 (Trial design, duration of treatment), 9.2.1, 12.1, & Figure 4 updated to clarify that participants will receive a course of up to eight infusions over four weeks<br>• Section 11.3.9 & Table 1 (Footnote #16) updated to include fasting for at least 3 hours before dosing but should take their cardiovascular medications with a sip of water. This has been reduced from six to three hours for patient comfort. Patients are not undergoing general anaesthesia, hence a prolonged fasting time is unnecessary.<br>• Section 10.4, Table 1 and Section 11.3.2.10 have been updated to clarify that scores relating to sleep, appetite and medication adherence are carried over during visits. These include items 4 and 5 on the MADRS, and items 1,2,3,4,6,7,8,9 & 17 on the QIDS-SR. These scores will be carried over from -40 minutes before each infusion to +60 minutes, +120 minutes, and +24 hours after each infusion. This is in line with the use of the MADRS in other similar ketamine trials.<br>• Section 11.2.2., Section 11.3. (Table 1 and footnote #5), Section 11.3.2.6. and Section 11.3.8. Not all patients have an ECG done on admission. For logistical reasons, timing of pre-infusion ECG has been extended to

		include period from admission up to first infusion and review by the trial anaesthetist.<br>• Section 11.3 Table 1 and Abbreviations, Section 11.3.2.1. To minimise patient burden, we are removing the NART assessment from the protocol as it is not essential.<br>• Section 7 (Key Exclusion Criteria) and Section 11.2.3. Clarification of exclusion criteria regarding comorbid Axis 1 diagnoses.<br>• Section 11.3 Table 1 footnotes. We have broadened the time for completion of pre-infusion MADRS (-40 +/- 20 mins) to facilitate clinic logistics.<br>• Section 11.2.3: We have clarified exclusion criteria to be in line with SmPC's for both ketamine and midazolam. Patients can be randomised to either midazolam or ketamine. Bradycardia is a known adverse effect of midazolam and ketamine. Therefore, patients with pre-existing bradycardia will be excluded.<br>• Sections 11.3.4. and 11.3.9. We have removed "or 20% increase" referring to blood pressure increase lasting more than 15 minutes that would render a patient ineligible to continue in the trial. People with low-normal blood pressure can have a 20% increase yet still be within the normal range of blood pressure. We have clarified these sections to note that only persisting non-physiological changes that do not respond to treatment with beta blocker and that last for more than 15 minutes will lead to discontinuation from the trial.<br>• Section 11.3 (Table 1, footnote #14) and Section 11.3.2.9. The previous amendment (see date 2nd Sept 2020) should have been to uniformly correct collection of concomitant medication at Visit 0 from two months prior to baseline to one month. We have rectified this previous error to one month throughout the Protocol.
Protocol Version 5.0	10/Oct/2022	• Sections 7 and 11.2.3. Exclusion criteria with regards to psychiatric comorbidities have

		been clarified and amended to bring in line with recent major ketamine trials.<br>• Data entry procedure has been changed from single to double entry to help minimise data entry errors.
Protocol Version 6.0	17/May/2023	• Typographical and grammatical errors have been corrected throughout the text.<br>• The former name (NUIG) for the University of Galway has been replaced with the new name throughout the text.<br>• The millilitre amount of intravenous fluid has been removed throughout the text since the amount of fluid infused depends on the IMP and the weight of the patient.<br>• In Section 17, data retention period has been updated to 25 years in accordance with Clinical Trial Regulation (CTR) (EU Regulation 536/2014) as this trial will soon be transitioning to CTR.<br>• Statistical analysis plan in Sections 7 and 14 have been updated in line with the Statistical Analysis Plan V2.0 approved by the Data Monitoring Committee and the Trial Steering Committee.<br>• In Section 11.3.2.3 and Table 1, the CTQ and the SAPAS have been removed to reduce participant burden as they are non-essential and the study will be underpowered for these analyses.<br>• In Sections 7 and 10.3, exploratory objective 6 has been removed as the study will be underpowered to address this research question.<br>• The descriptive term “severe” depression has been removed throughout the text to make the text consistent with the MADRS rating scale cut-off of 20 for entry which technically also includes moderate depression.<br>• Concomitant medications are recorded primarily for safety reasons before and during the randomised treatment period (Visits 0-8). To ensure accuracy and full visibility to the monitoring staff, medications for Visits 0-8 are now being exported directly from the participant’s electronic health record instead of manually recorded on a handwritten medication log. At long-term follow-up (Visits 9, 10 and 11), concomitant medications will

		continue to be recorded on another scale (CSRI). To avoid duplication, the concomitant medication log is no longer recording medications at these long-term follow-up timepoints. This has been clarified in Section 11.3.2.9. and Table 1.<br>• There is no item 17 on the QIDS-SR_{16}. This error has been rectified throughout the text.<br>• It has been clarified in Table 1 footer that BMI is a derived variable from height and weight which are recorded variables.<br>• In Section 11.3.2.1. and Table 1, the assessment of treatment-resistant depression has been simplified from the dimensional Maudsley Staging Method (MSM) to the categorical Antidepressant Treatment Response Questionnaire (ATRQ). This is to bring the methodology of the trial in line with other major trials in this area. There is no additional participant burden with the change from MSM to ATRQ since all the necessary information to rate the ATRQ has already been collected for all existing participants as part of the MSM.<br>• In Section 9.2.2., it has been clarified that “low” doses refer to subanaesthetic doses.<br>• In Section 11.3.4. (iv), recent references have been added to support the statement that there is no evidence of a withdrawal syndrome in the published ketamine literature to date. The risk of withdrawal remains a theoretical concern. The literature on ketamine safety is monitored on an ongoing basis by the investigators. Should any new concerns arise in the future, the Protocol and the PIL will be updated as appropriate.<br>• In Section 11.3.6., the term “Withdrawal eCRF” was replaced with the correct term “End of Study Form”.<br>• In Section 13.2.5., the location of the displaying of the Emergency unblinding procedure has been updated.<br>• In Section 16.1., it has been clarified that information exported from the electronic health record cannot be pseudonymised. These data are filed separately from the pseudonymised source data documents.

		• It has been clarified throughout the text that AEs are followed up until resolution or final visit.<br>• Section 23 has been updated to remove the section about trial newsletter.<br>• The role of the sponsor in publication activities was removed in Section 23.<br>• The Department of Psychiatry, Trinity College Dublin affiliation has been added to Section 23<br>• The definition of relapse (Section 11.3.3) has been updated to bring in line with recent major trials of antidepressants in relapse prevention.
Protocol Version 6.1	12/Oct/2023	• It has been clarified throughout the document that depression can be "moderate or severe" at trial entry.<br>• Population figure for Ireland has been updated with a 2023 estimate on Page 17.

7 SYNOPSIS

Title of study	Ketamine as an adjunctive therapy for Major Depression (2) - a randomised controlled trial: [KARMA-Dep (2)]
Name of Sponsor/company	Trinity College Dublin
Phase of development	Phase III
Objectives	**Objective 1**: To conduct a pragmatic randomised controlled patient- and rater-blinded trial of repeated adjunctive twice-weekly ketamine vs. midazolam infusions over up to four weeks for patients hospitalised for moderate or severe depression and assess the mood-rating score difference between arms from before the first infusion to 24 hours after the final infusion, supplemented by a 95% confidence interval. There will also be a 24-week follow-up after the final infusion session.<br><br>**Objective 2:** To assess response and remission rates at the end of the randomised treatment phase and, relapse status after 24 weeks.<br><br>**Objective 3**: To assess the safety and tolerability in inpatients with moderate or severe depression of repeated (up to 8)

	infusions of ketamine vs midazolam regarding psychotomimetic, other psychiatric, and cognitive side-effects.<br><br>**Objective 4:** To assess the safety and tolerability in inpatients with moderate or severe depression of repeated (up to 8) infusions of ketamine vs midazolam regarding haemodynamic stability, neurological, urological, and other physical health side-effects.<br><br>**Objective 5:** To conduct quality-of-life and cost-effectiveness analyses for patients participating in the KARMA-Dep (2) Trial.
Trial design	This proposed trial is a pragmatic, randomised, controlled, parallel-group, superiority trial. Eligible consented participants will be randomly allocated in a 1:1 ratio to a course of up to eight infusions of either ketamine or midazolam twice weekly over up to four weeks. To facilitate generalisabilty of results, the trial will take place under "real world" conditions with both groups continuing usual in-patient care (e.g. regular medications, nursing care, psychological and other therapies) during the randomised treatment phase and usual out-patient care and review thereafter. Participants will be followed-up for 24 weeks after the end of the initial randomised treatment and assessment period to identify if and when relapse occurs. During the allocated infusions and follow-up period patients will be monitored for treatment-related adverse events relating to both mental and physical health.
Key inclusion criteria	• ≥18 years old<br>• Montgomery-Åsberg Depression Rating Scale (MADRS) score ≥20 at screening and start of the first infusion<br>• Voluntary admission for treatment of an acute depressive episode<br>• Meet DSM-5 criteria for a major depressive disorder (MDD) or bipolar affective disorder (current episode depression)
Key exclusion criteria	• Current involuntary admission<br>• Medical condition rendering unfit for ketamine/midazolam<br>• Currently taking any of the contraindicated medications listed in section 12.7.2<br>• Active suicidal intention<br>• Dementia<br>• Lifetime history of schizophrenia or schizoaffective disorder; active anorexia nervosa or bulimia nervosa

	in the past 12 months; alcohol or other substance use disorder (with the exception of nicotine) in the previous six months; any DSM-5 disorder other than a major depressive episode (unipolar or bipolar) as the primary presenting problem<br>• Electroconvulsive Therapy (ECT) administered within the last two months<br>• Pregnancy, breastfeeding or considering becoming pregnancy whilst on the trial for up to 12 weeks after last dose or inability to confirm use of adequate contraception during the trial<br>• Breastfeeding women
Number of patients	We aim to recruit 104 patients on admission who will be eligible for this study and randomly allocate 52 patients to each group.
Test product, dose and mode of administration	**Investigational Medicinal Product:** Ketamine: Ketalar 10mg/ml Solution for Injection/Infusion, Pfizer Ireland; 0.5mg/kg of body weight.<br><br>**Active comparator:** Midazolam: Hypnovel 10mg/5ml solution for injection, Roche Pharmaceuticals Ireland; 0.045 mg/kg of body weight.<br><br>Both made up as colourless saline solutions and administered over 40-minutes using an infusion pump to deliver the required total amount of ketamine/midazolam as per individual body weight, in an up to four-week course of twice-weekly infusions, i.e. maximum of eight infusions.
Duration of treatment	Up to four weeks
Statistical methods	Analyses will be conducted in the intention-to-treat set. The primary analysis will be conducted once at end-of-trial by a statistician blinded to group labels. To produce this, a general linear model will be fitted to the MADRS scores, with trial arm, site and baseline MADRS as covariates, and using heteroscedasticity-consistent “sandwich” variance-covariance estimators. The dependent variable will be the MADRS scores 24 hours after the final infusion, including where the full course of infusions was not completed.<br><br>The efficacy of the treatment will be evaluated by way of a statistical test of the coefficient for trial arm, the p-value for which will be compared to 0.05. The coefficient itself will be presented as the effect size, corresponding to an adjusted MADRS score mean difference between arms after the final infusion, and supplemented by a 95% confidence interval. For

	a single primary outcome, no adjustment to the type I error is needed.
Sample size	Based on a randomised trial of adjunctive serial ketamine infusions [1], 41 patients are required per initial randomisation group (n=82) to have 90% power to demonstrate, using a two-sided t-test at 5% level, that mean reduction in MADRS score in the ketamine group will be ≥8 points that achieved in midazolam group. This calculation conservatively assumes a standard deviation for the change in mean MADRS of 11, and that the assumptions of a t-test are broadly met, which is expected to be the case with approximately 40 patients per group. Allowing for a 20% dropout, we will recruit 52 patients per group (total n=104).

8 ABBREVIATIONS

AE	Adverse event
AMPAR	α-amino-3-hydroxy-5-methyl-4-isoxazolepropionic acid receptors
AR	Adverse reaction
ATRQ	Antidepressant Treatment Response Questionnaire
BP	Blood pressure
BPRS	Brief Psychiatric Rating Scale
CA	Competent authority
CADSS	Clinician-Administered Dissociative States Scale
CDMS	Clinical Data Management System
CI	Chief investigator/Co-ordinating investigator
CRF	Case report form
CRFG	Clinical Research Facility at the University of Galway
CSRI	Client Service Receipt Inventory
CT	Clinical trial
CTA	Clinical trial authorisation
DMC	Data Monitoring Committee
DMF	Data Management File
DMP	Data Management Plan
DSM-5	Diagnostic and Statistical Manual of Mental Disorders, Fifth Edition
DSUR	Data Safety Update Report
ECG	Electrocardiogram
EQ-5D-5L	Five-level version of the EuroQol five-dimensional questionnaire
EU	European Union
eCRF	Electronic case report form
GCP	Good Clinical Practice
GP	General Practitioner
HPRA	Health Products Regulatory Authority
HRSD-24	Hamilton Rating Scale for Depression, 24-item version
HSE	Health Service Executive
ICF	Informed consent form

ICH	International Conference on Harmonisation
IMP	Investigational medicinal product
ISO	International Organization for Standardization
I.V.	Intravenous
MADRS	Montgomery-Åsberg Depression Rating Scale
MDD	Major Depressive Disorder
MoCA	Montreal Cognitive Assessment
NCI CTCAE	National Cancer Institute Common Terminology Criteria for Adverse Events
NMDA	N-methyl-D-aspartate
OAA/S-R	Observer's Assessment of Alertness/Sedation scale – Responsiveness subscale
PVO	Pharmacovigilance Office
PI	Principal investigator
PIL	Patient Information Leaflet
PRISE	Patient-Rated Inventory of Side Effects
PWC-20	20-item Penn Physician Withdrawal Checklist
QIDS-SR_{16}	Quick Inventory of Depressive Symptoms - Self-Report, 16-item
REC	Research Ethics Committee
RSI	Reference safety information
SAE	Serious adverse event
SAR	Serious adverse reaction
SmPC	Summary of product characteristics
SOP	Standard operating procedure
SSRI	Selective Serotonin Re-uptake Inhibitor
STAR*D	Sequenced Treatment Alternatives to Relieve Depression
SUSAR	Suspected unexpected serious adverse reaction
TSC	Trial Steering Committee
YMRS	Young Mania Rating Scale

9 INTRODUCTION

9.1 Background information

9.1.1 DEPRESSION – THE SCALE OF THE PROBLEM

Major depressive disorder (MDD) is a debilitating mental illness with a lifetime prevalence of 12-20% and one-month prevalence of 5%. Women are affected 1.5 times as much as men. The core features of a depressive episode are pervasive low mood, anergia, and anhedonia present for at least two weeks and accompanied by somatic symptoms (e.g. sleep and appetite disturbance), cognitive impairments, and negative cognitions. Severe depression can be life-threatening due to: extreme self-neglect; psychomotor retardation leading to stupor/catatonia; psychosis; and/or suicidal ideation and intent. Such severely ill patients often require hospitalization.

According to the World Health Organisation, depression is now the leading cause of disability worldwide (http://www.who.int/mental_health/management/depression/prevalence_global_health_estimates/en/). In Europe, depression is the most costly brain disorder, accounting for 1% (€118 billion annually) of the total EU economy [2]. Much of this is due to treatment-resistance, with 30% of patients failing to respond to two or more trials of antidepressant medications and/or

psychotherapies [3]. Also, about 50% of people who experience one depressive episode will have further episodes, with 5-9 episodes in their lifetime. Even following successful antidepressant therapy for acute episodes, relapse rates are high, ranging 40-70% [3].

In Ireland alone, with a population of 5.28 million people, 6,000 require hospitalization annually for depression, accounting for 30% of in-patient admissions to mental health services. Depression is thus a major national and global public health concern because of its high prevalence and societal costs; additionally, it can lead to chronic disability, is a risk factor for both dementia and stroke in later life, and 6% will die by suicide.

Despite intensive research efforts, the standard pharmacological care for depression over the past 60 years has remained focused mainly on monoamine neurotransmitters, e.g. selective serotonin re-uptake inhibitors (SSRI), serotonin-norepinephrine reuptake inhibitors, tricyclic antidepressants. The large STAR*D study in the USA found that only 30% of patients achieved remission after first-line SSRI treatment and half of MDD patients did not remit after two antidepressant trials [3]. Moreover, these standard treatments can take weeks to work with less than 40% of patients remitting within 10-14 weeks, highlighting the need for novel treatment approaches. One such approach might be the dissociative anaesthetic ketamine.

9.1.2 KETAMINE – A NEW ERA OF RAPID-ACTING ANTIDEPRESSANTS

Ketamine is a routinely used and relatively inexpensive anaesthetic. It is usually given intravenously (i.v.) and has a short half-life (2-3 hours). It is an antagonist of the N-methyl-D-asparate receptor (NMDAR) and targets the excitatory neurotransmitter glutamate [4]. Ketamine is a racemic mixture of *R*- and *S*-ketamine (also known as esketamine) enantiomers which in turn are converted to (R)- and (S)-norketamine and -hydroxynorketamine metabolites. Esketamine has been developed by the *Janssen* pharmaceutical company as an intranasal formulation for treatment-resistant depression and was approved by the FDA in the USA in March 2019. Intranasal esketamine has not yet been approved for depression in the EU and the cost and how its use will be supervised remain to be determined [5].

The precise antidepressant mechanism of ketamine is not known and may be related to glutamate activation, rather than inhibition, and/or effects of hydroxynorketamine metabolites on glutamate binding to α-amino-3-hydroxy-5-methyl-4-isoxazolepropionic acid receptors (AMPARs) [4].

Single, slowly administered, sub-anaesthetic ketamine infusions elicit rapid, though transient, antidepressant responses and target core symptoms in treatment-resistant depression, including suicidal ideation [6-9]. A 40-minute 0.5mg/kg of body weight i.v. infusion has been the most effective dose to date for both unipolar and bipolar depression, with lower doses having less of an antidepressant effect and higher doses causing intolerable dissociative side-effects [7, 10, 11]. Depressive symptoms improve within one-hour of single infusions, with peak effect-size at 24-hours (e.g. Hedges' g= -1.0, 95% CI 1.28 to 0.73, $p<0.001$) [6] and lasting up to 5-8 days, i.e. beyond immediate NMDAR blockade. Other methods to administer ketamine are being evaluated, including intramuscular and subcutaneous injections, oral ingestion, and intranasal sprays. While these methods are easier to administer than slow i.v. infusions, they may be less predictable regarding bioavailability and definitive randomized comparisons with i.v. infusions are lacking.

Together, these findings represent a paradigm shift away from conventional slow-acting monoaminergic antidepressants to a potential new era of rapid-acting antidepressants. However, a definitive role for ketamine in managing depression is not yet agreed and there are concerns about unregulated off-label use and associated lack of oversight [10].

9.1.3 REPEATED KETAMINE INFUSIONS FOR DEPRESSION

Apart from its potential role in the acute management of suicidal ideation [9], it is unlikely that the transient effects of single ketamine infusions are of practical therapeutic value for treating depression. The majority of randomized trials have reported on studies using single ketamine infusions and these have been the focus of the most recent meta-analyses, demonstrating robust but transient antidepressant effects [6-9].

Not surprisingly, repeated sub-anaesthetic ketamine infusions have more sustained antidepressant effects. In an open-label trial of unmedicated patients with recurrent MDD (n=24), Murrough *et al* (2013) reported a 71% (17/24 patients) response rate after two weeks of thrice-weekly ketamine infusions [12]. Of these 17 responders, 13 (76%) relapsed during an 83-day follow-up with median time to relapse of 18 days. Shiroma *et al* (2014) reported a similar open-label trial of two weeks of thrice-weekly ketamine infusions in which recurrent MDD patients (n=14) remained on stable doses of antidepressant medications [13]. Eleven (86%) participants were deemed responders and six (55%) relapsed during a 28-day follow-up period with mean time to relapse of 16 days. Repeated ketamine infusions were reported as being generally safe and reasonably well-tolerated during both studies.

Only two randomized, placebo-controlled, trials of repeated sub-anaesthetic ketamine infusions have been reported to date. In the first study, Singh et al (2016) randomized patients with recurrent MDD (n=67) to one of four treatment groups based on twice vs thrice-weekly infusions of ketamine (0.5 mg/kg) vs saline placebo [1]. Patients continued stable doses of antidepressant medications. The initial double-blind phase was for two weeks and participants did similarly well in the twice- and thrice-weekly ketamine groups. Response criteria were met by 11/18 (69%) in the ketamine group and only 2/13 (15%) in the placebo group. Drug-related side-effects were more common in the twice-weekly ketamine group (72% of patients) than the placebo group (38%) and were similar to those reported in previous studies, i.e. headache, anxiety, dissociation, nausea, dizziness. Two patients in the ketamine group (11%) and one (6%) in the placebo group discontinued because of treatment-emergent adverse events. In the second trial, out-patients with MDD plus chronic (i.e. ≥3 months) suicidal ideation (n=26) were randomized to six sessions of twice-weekly infusions of ketamine (0.5 mg/kg) vs saline placebo while remaining on a stable medication regimen [14]. At the end of the infusion phase and at the three-month follow-up there were no differences between the groups regarding mood or suicidal ideation. At the end of the infusion phase response criteria were met by 3/12 (25%) in the ketamine group and 4/12 (33%) in the placebo group. The authors suggested that a higher dose of ketamine may be required in depressed patients with chronic suicidal ideation, which has often been an exclusion criterion in ketamine trials to date.

9.1.4 INTRANASAL ESKETAMINE FOR DEPRESSION: INDUSTRY-FUNDED TRIALS

More recently, in an industry (Janssen)-funded trial, twice-weekly intranasal esketamine at three different doses (28 mg, 56 mg, 84 mg) has also been reported to have sustained, dose-dependent, antidepressant effects over two weeks in a randomized placebo-controlled trial involving medicated patients with MDD (n=67) [15]. The response rate after two weeks in the highest-dose (84 mg) group was 42% (5 out of 12 allocated patients) compared to 6% (2/33) in the placebo group. A subsequent larger industry (Janssen)-funded trial (n=346) found no significant difference (mean difference [95% CI] of -3.2 [-6.88, -0.45] points on the MADRS depression scale) between four weeks of twice-weekly fixed-dose intranasal esketamine (84mg) and placebo spray added to a newly-started open label oral antidepressant [16]. However, a similar study (n=227) but with flexibly-dosed esketamine (56-84 mg) reported a statistically significant, though only slightly larger, benefit (mean

difference [95% CI] of -4.0 [-7.31, -0.64] points on the MADRS). Interestingly, continuing once-weekly esketamine nasal spray, compared to placebo spray, in addition to an oral antidepressant has been reported to reduce relapse rates (26.7% and 45.3%, respectively; hazard ratio 0.49, 95% CI 0.29-0.84) over 44 weeks in depressed patients who previously met stable remission criteria with open label esketamine nasal spray plus oral antidepressant [5].

9.1.5 KETAMINE FOR DEPRESSION: SYSTEMATIC REVIEWS

The above studies demonstrate that the antidepressant efficacy of ketamine infusions can be maintained with repeated administrations though relapse rates appear to be high once infusions are stopped. It may also be that ketamine infusions are more efficacious than intranasal esketamine, though this remains to be formally evaluated. However, some caution is warranted in interpreting the generalizability of these efficacy studies due to small sample sizes, exclusion of patients >65 years, focus on outpatients, restriction on other therapeutic options, lack of an "active" placebo to help mask some of the non-antidepressant effects of ketamine [17], brief follow-up, and industrial sponsorship. Nor is it clear if once-weekly ketamine infusions, which can have effects lasting up to one week [6-9], are as effective as twice-weekly infusions.

There have so far been two *Cochrane Reviews* on ketamine and other glutamate modulators for both depression and bipolar depression in adults [18, 19]. Noting bias due in a large part to small sample sizes, the Cochrane depression review found that i.v. ketamine increased the likelihood of response after 24 hours (OR 10.8, CI 2.0 to 58.0; 3 RCTs, 56 participants) but effects were less certain after one-two weeks. The Cochrane bipolar depression review was similar: after 24 hours ketamine increased the likelihood of response (OR 11.6, CI 1.25 to 107.7; 2 RCTs, 33 participants) and the statistically significant difference disappeared after three days. There has been no update on these Cochrane reviews but more recent meta-analyses have larger sample sizes, including up to nine trials with 368 patients [6-8]. Kishimoto *et al* (2016) [5] identified nine ketamine RCTs (n=234) and found depressive symptoms improve within one-hour of single infusions, with peak effect-size at 24-hours (studies=7, Hedges' g = −1.00, 95% CI −1.28 to −0.73, p<0.001; heterogeneity: τ2 = 0.00, I2 = 0.00, Q = 2.14, p=0.91) and this lasted for up to 5-8 days (studies=5, Hedges' g = −0.38, 95% CI −0.73 to −0.03, p=0.036; heterogeneity: τ2 = 0.02, I2 = 9.38, Q = 4.41, p=0.35). Most recently, with a slightly larger sample size, Han *et al* had similar findings [8]. They identified eight RCTs (n=301) for which response rates were available at 24 hours after a single ketamine infusion; the pooled OR was 10.09 (95% CI: 4.96–20.52, z=6.38, P=0.00001) with low heterogeneity (*I*2=0%, P=0.57) and no evidence of publication bias.

Between them, the *Cochrane Reviews* and subsequent meta-analyses confirm the acute rapid-onset effects of low-dose ketamine infusions and that these effects wear off within one week. In their conclusions, the reviews collectively highlight the need for better-quality, larger, and longer-term parallel group trials to assess safety and efficacy, including using repeated ketamine infusions. The proposed definitive *KARMA-Dep (2) Trial* will address these recommendations.

9.1.6 THE KARMA-Dep PILOT TRIAL (EudraCT number: 2016-004764-18; ClinicalTrials.gov Identifier: NCT03256162)

To inform the present definitive KARMA-Dep (2) Trial, we recently completed a HPRA-approved randomized pragmatic pilot trial of four once-weekly 40 min infusions of ketamine (0.5 mg/kg) compared to midazolam (0.045 mg/kg) as adjunctive therapy for patients hospitalized with depression. The aim of this pilot trial was to assess trial processes not to test hypotheses [20].

Between September 2017 and June 2018 admissions to St Patrick's University Hospital were assessed for eligibility. In total, 1581 admissions were reviewed. Of these, 125 (8%) were eligible to participate in the pilot trial and 25 (20%) agreed to participate. The recruitment rate is shown in Figure 1. Thirteen were randomly assigned to the ketamine arm and 12 to the midazolam arm. There were no differences in baseline HRSD-24 mood scores between the two groups (Figure 2). The difference between the ketamine and midazolam groups with regards to the changes in mood scores at the end of the allocated infusion phase (Follow-up week 1, Figure 2) was -2.6 [95% CI -8.26 to 3.03] points on the HRSD-24 depression scale with some persisting benefit for the ketamine in the remainder of the 12-week follow-up.

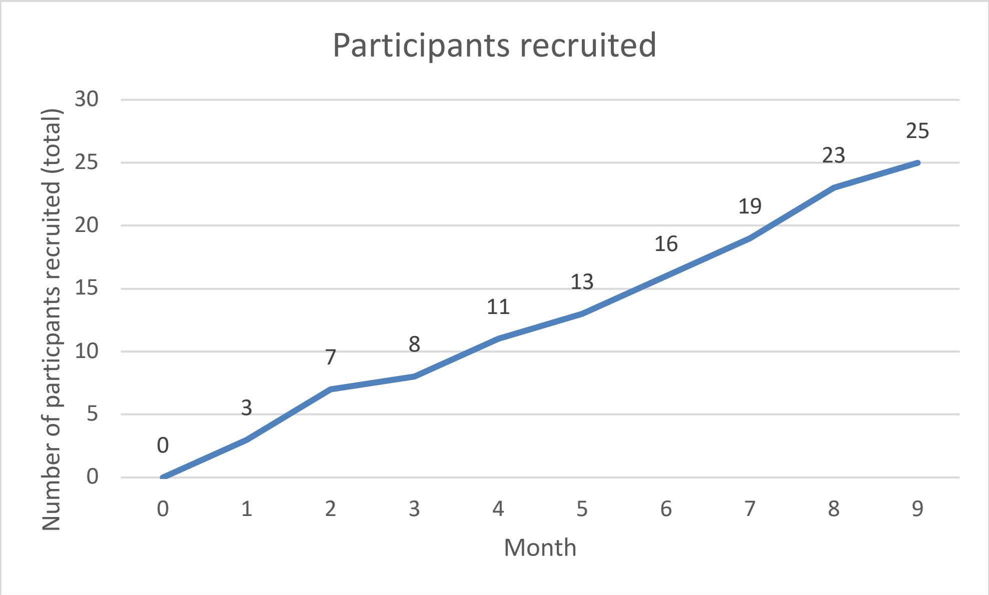

Figure 1: KARMA-Dep Pilot Trial recruitment accrual on a monthly basis.

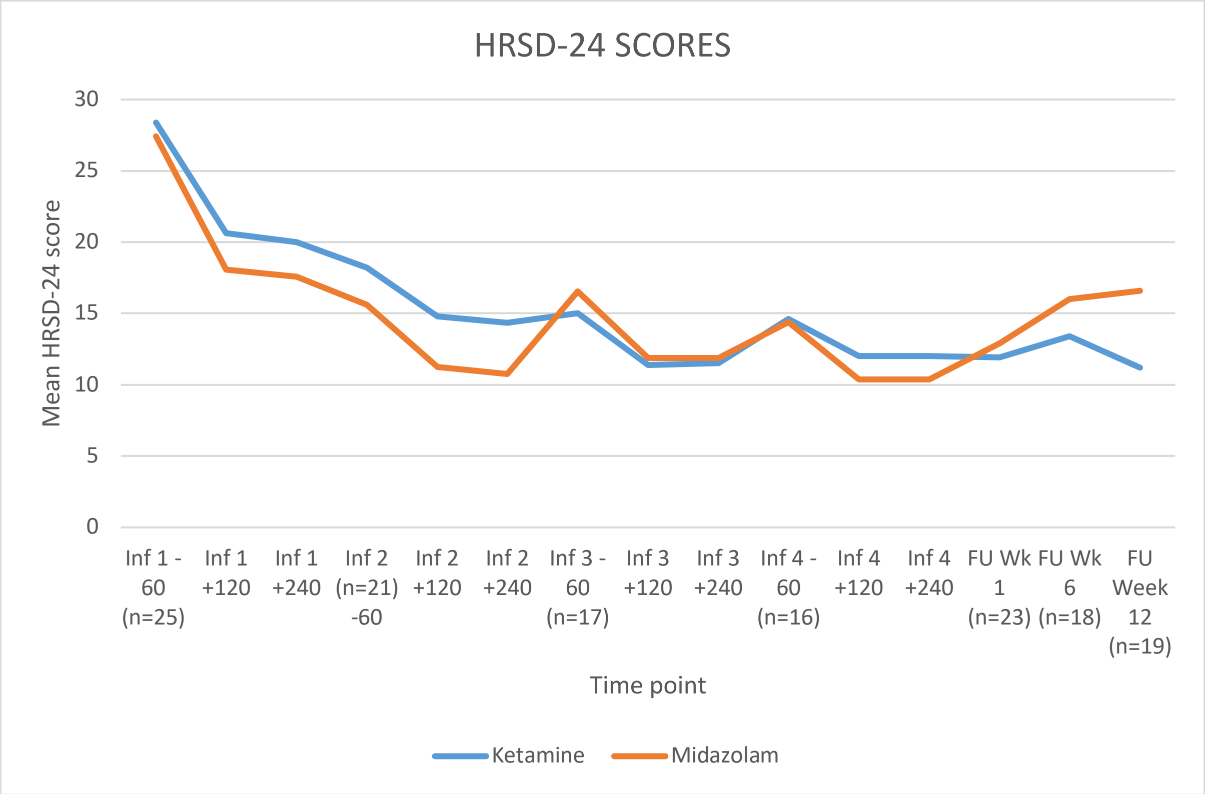

Figure 2. Mean HRSD-24 scores for both arms throughout the pilot trial. Data for all patients are shown are shown. Inf, Infusion; -60, 60 minutes pre-infusion; +120, 120 minutes post commencement of infusion; +240, 240 minutes post commencement of infusion; FU, Follow-up; Wk, week; n, total number of participants at each timepoint.

The infusions were generally safe and well tolerated. As expected, dissociative side-effects were greater in the ketamine group, but were generally mild and transient, resolving shortly after infusions finished and diminished with subsequent infusions (Figure 3) [21, 22].

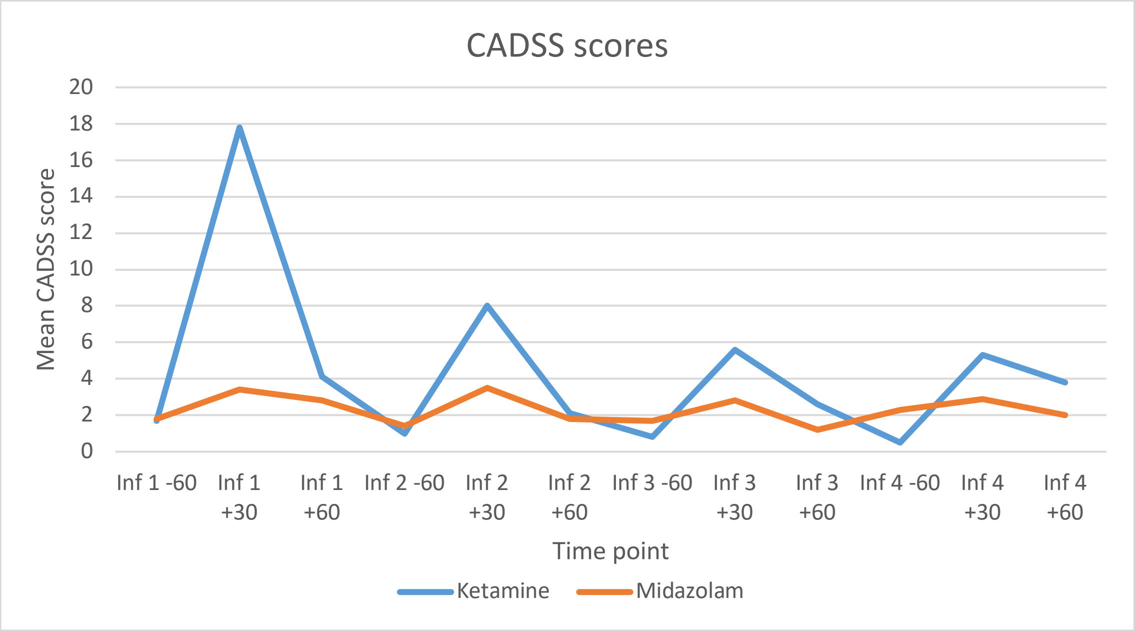

Figure 3 Mean CADSS scores during the four infusion sessions. The higher the CADSS score the more dissociated effects reported by trial participants [23]. Inf, Infusion; -60, 60 minutes pre-infusion; +30, 30 minutes post commencement of infusion; +60, 60 minutes post commencement of infusion.

All patients who were randomised had at least one infusion. Sixteen patients (64%) completed all four infusions, eight in the ketamine groups and eight in the midazolam group.

In the ketamine group, four patients stopped after one infusion (two of these were due to adverse events, one transferred to a programme with conflicting timetable and one was prescribed ECT) and one stopped after two infusions (discharged from hospital due to feeling well). The adverse events that resulted in patients stopping treatment after the first infusion were intolerable dissociative side-effects (one patient) and intolerable dissociative side-effects with vomiting and brief, but significant, increase in blood pressure (one patient).

In the midazolam group, three patients stopped after infusion two and one after infusion three. Reasons for discontinuing the trial were: nightmares during two infusions; referred for ECT; discharged from hospital; and left hospital against medical advice.

Retention rates of patients was good. Nearly all of the patients participated in follow-up assessments: at one week follow-up, 23/25 (92%) competed their assessments; 18/25 (72%) at week 6; and 19/25 (76%) at week 12. During the infusions and 12-week follow-up period there were no Serious Adverse Events/Reactions or Suspected Unexpected Serious Adverse Reactions (SUSAR).

In conclusion, the KARMA-Dep Pilot Trial was the first pragmatic pilot trial of adjunctive serial ketamine infusions for hospitalized depression, an important possible use of ketamine. This study shows that a definitive trial of adjunctive ketamine is feasible. In contrast to most other studies that found relatively large differences between twice-weekly ketamine and saline/midazolam comparator, we used once-weekly infusions. For the definitive KARMA-Dep (2) Trial we therefore decided to change from once-weekly to twice-weekly infusions.

Based on our pilot trial recruitment and follow-up data, we have added a second recruitment site (i.e. St Patrick's Hospital Lucan, Co. Dublin) to the definitive trial to increase recruitment to approximately four patients/month to achieve recruitment targets in a timely fashion. Additionally,

our experience in infusion clinics is that reported and observable side-effects are limited to the infusion period (40 mins) and resolve within one hour. After this time, it is not beneficial to keep the patients in the clinic. To optimize retention, for the definitive trial, we have therefore reduced the post-infusion observation period from +240 mins to +120 mins from the beginning of infusions. We have also extended the follow-up duration to 24 weeks as the lack of longer-term outcome data is a common theme in systematic reviews that needs to be addressed.

Another planned minor change is to use the MADRS depression rating scale instead of the HDRS-24, as this instrument is being used more commonly in ketamine trials. This will more readily allow direct comparison between our definitive KARMA-Dep (2) Trial and other relevant trials and facilitate pooling data for future meta-analyses.

Our group has also completed two HPRA-approved trials of ketamine for depression relapse prevention (NCT024114932 and NCT02661061) [24, 25]. We therefore have the appropriate expertise and resources to successfully complete the proposed definitive KARMA-Dep (2) Trial, which is being funded by the Health Research Board.

9.1.7 THE DEFINITIVE KARMA-Dep (2) TRIAL

One practical therapeutic role for ketamine may therefore be as an adjunctive agent to on-going treatment for depression, especially for persons with depression severe enough to be hospitalised. This would entail serial ketamine infusions for a defined period of up to four weeks, which in itself would help limit concerns about prolonged repeated use, safety, and risk of dependency. The **main hypothesis** that underlies this trial therefore is that repeated ketamine infusions (twice-weekly, max 8 infusions) as adjunctive therapy to routine care will improve depression outcome in patients hospitalized with moderate or severe depression. As a **secondary hypothesis**, we also predict that this will be associated with reduced health care costs and improved quality of life. The results of the proposed trial should be generalizable to patients with depression hospitalized in industrialized nations but may be applicable more internationally and to inpatients with moderate or severe depression in general.

9.2 Rationale for the study

9.2.1 Investigational Medicinal Products

Ketamine (as hydrochloride; 0.5 mg/kg; Pfizer Healthcare Ireland) and midazolam (as hydrochloride; 0.045 mg/kg; Roche Products Ireland Ltd) will be made up as colourless saline solutions and administered over 40-minutes using an infusion pump, in a course of up to eight infusions twice-weekly over up to four weeks. A recent study comparing different doses of single i.v. ketamine infusions, when added to stable antidepressant therapy, found that doses below 0.5mg/kg were not clinically effective and that a dose of 1.0 mg/kg was not more effective than 0.5 mg/kg [26]. We are therefore continuing to use ketamine infusions at a dose of 0.5 mg/kg over 40 minutes. It is now well-established that repeated sub-anaesthetic doses of ketamine are well-tolerated by psychiatric populations [27]. It is hypothesised that a course of ketamine infusions over this schedule may improve recovery in patients with moderate or severe depression admitted to hospital when compared with the active comparator, midazolam.

Midazolam itself is not an antidepressant medication. It was chosen as an active comparator to help maintain blinding because it has similar acute sedative effects as ketamine at sub-anaesthetic

doses and a similar half-life [28, 29]. Additionally, compared to saline, midazolam has been shown to result in greater antidepressant control effects and is therefore considered to demonstrate a more realistic estimate of the antidepressant effect of ketamine in randomised trials [17].

9.2.2 Risks and benefits

1. Physical:

(i) Risks and Hazards associated with Ketamine: for more detail, please see Summary of Product Characteristics (SmPC). Ketamine is psychotomimetic (with abuse potential) but is safe at low (subanaesthetic) doses, with patients and healthy controls experiencing mild dissociative and psychotic symptoms (which can be unpleasant) that resolve soon after finishing infusions. In subanaesthetic doses, ketamine is a safe drug but can cause transient rises in pulse and blood pressure during infusion and for up to 80 minutes afterward. Thus, monitoring procedures will be followed as described in section 11.3.4. A recent review of ketamine in depression concluded that outside recreational usage, there have been no reports of persistent adverse effects with subanaesthetic uses of ketamine [30].
Classification: Transient to serious.
Probability: Serious adverse effect=Rare. Non-serious adverse effect=Unlikely.
Transient adverse effect=Common.

(ii) Risks and Hazards associated with Midazolam: for more detail, please see Summary of Product Characteristics (SmPC). Midazolam has recently been used as an active comparator to ketamine in parallel-group design trials as it mimics some of the effects of ketamine and may improve blinding over inactive placebo saline [17]. At subanaesthetic doses there have been no reported serious adverse events. However transient physical symptoms can occur during infusions, including minor lowering of blood pressure. The monitoring procedures detailed in section 11.3.4 have been put in place to ensure any possible harm is minimised. Infusions will be administered by an experienced Consultant Anaesthetist with assistance from researchers in a facility equipped for general anaesthesia and resuscitation.
Classification: Transient to serious.
Probability: Serious adverse effect=Rare. Non-serious adverse effect=Unlikely.
Transient adverse effect=Common.

(iii) Risks and hazards associated with peripheral venous cannulation and intravenous administration of investigative medicinal products:
Complications that can arise following the procedure of cannulation include infiltration, extravasation, venous spasm, phlebitis, thrombophlebitis, haematoma, nerve injury, arterial puncture, embolism and needle stick injury. In this study, peripheral venous cannulation will be performed by a consultant Anaesthetist using aseptic technique and in accordance with the local venous cannulation policy. Cannulae will be used for a 40-minute infusion and removed prior to discharge from the infusion clinic. The cannulation site will be monitored during the infusion and regularly for up to 120 minutes from the beginning of the infusion. Discomfort is common during the insertion of a peripheral venous cannula, however every effort will be made to minimise pain or discomfort, including the use of topical anaesthetics where indicated.
Classification: Transient to serious.
Probability: Serious adverse effect=Rare, Transient adverse effect=Common.

2. Psychological:

(i) Distress: Some participants may find questionnaires distressing or anxiety-provoking, or the experience may change the way they view or manage their illness. This can be difficult to predict. However, researchers will be vigilant for possible negative psychological effects and seek to minimise these wherever possible. In the case of patients, where distress is noted, this will be brought to the attention of the treating team.
Classification: Transient.
Probability: Unknown

3. Psychosocial:

(i) Inconvenience: Attending for assessments or interventions may cause inconvenience to participants. We will seek to minimise lifestyle inconvenience by keeping assessments concise, accommodating participants on return visits to the hospital for non-trial-related appointments where possible, or telephone/ video conference assessments as necessary and reimbursing participants for travel and meal expenses where applicable.
Classification: Transient.
Probability: Common.

10 STUDY OBJECTIVE

The main aim of this randomised controlled trial is to test the hypothesis that repeated ketamine infusions as adjunctive therapy to routine care will improve mood outcome in patients hospitalised with moderate or severe depression.

10.1 Primary objective

To conduct a pragmatic randomised controlled patient- and rater-blinded trial of repeated adjunctive twice-weekly ketamine vs. midazolam infusions over up to four weeks for patients hospitalised for moderate or severe depression and assess the MADRS score difference between arms from before the first infusion to 24 hours after the final infusion, supplemented by a 95% confidence interval. There will also be a 24-week follow-up.

10.2 Secondary objectives

- To assess response and remission rates at the end of the randomised treatment phase and relapse status after 24 weeks.

- To assess the safety and tolerability in inpatients with moderate or severe depression of repeated (up to 8) infusions of ketamine vs midazolam regarding psychotomimetic, other psychiatric, and cognitive side-effects.

- To assess the safety and tolerability in inpatients with moderate or severe depression of repeated (up to 8) infusions of ketamine vs midazolam regarding haemodynamic stability, neurological, urological, and other physical health side-effects.

- To conduct quality-of-life, cost-effectiveness and cost-utility analyses for patients participating in the KARMA-Dep (2) Trial.

10.3 Primary and secondary/exploratory endpoints/outcome measures

The primary outcome is the change from baseline in the Montgomery-Åsberg Depression Rating Scale (MADRS) [31] score 24 hours after the final infusion. The MADRS is an observer-rated scale commonly used in research to measure the symptoms and severity of depression and is sensitive to change. It comprises 10 sets of questions on the following symptoms: 1. Apparent sadness; 2. Reported sadness; 3. Inner tension; 4. Reduced sleep; 5. Reduced appetite; 6. Concentration difficulties; 7. Lassitude; 8. Inability to feel; 9. Pessimistic thoughts; and 10. Suicidal thoughts. These questions address how the patient has felt over the past week and each item on the scale is rated from 0 to 6, with 0 being "normal/not present" and 6 being "extreme." Scores on items 4 and 5 will be carried over from -40 minutes before each infusion to +60 minutes, +120 minutes, and +24 hours after each infusion in line with the methodology of previous literature [32-35]. The higher the MADRS score the greater the severity of depression. Using a structured interview guide, the intraclass correlation for total score between raters has been reported as $r = 0.93$, with good to excellent interrater reliability for all ten items [36]. Standard criteria for depression severity, treatment response, remission and relapse will be used (please see definitions in section 11.3.3 below) in a 24-week follow-up schedule which involves the MADRS and other instruments at 6, 12 and 24 weeks post final infusion.

Secondary safety and tolerability outcomes consist of psychotomimetic, dissociative, cognitive and physical health effects of repeated ketamine infusions, measured before, during and after infusions using a range of validated instruments (see section 11.3.4 below).

11 TRIAL DESIGN

11.1 General considerations

This randomised, controlled, parallel-group, pragmatic, phase III trial will take place within St Patrick's Mental Health Services. Participants will be recruited from inpatients in St Patrick's University Hospital, Dublin 8, and its sister hospital St Patrick's Hospital Lucan, Co. Dublin. All patients will be treated in the Ketamine Research Clinic in St Patrick's University Hospital. The predicted trial flow is illustrated in Fig 4.

Figure 4: CONSORT Trial Flow Diagram [37, 38] The diagram includes numbers to be allocated and numbers predicted to be lost to follow-up and patients who might discontinue the allocated treatment course.

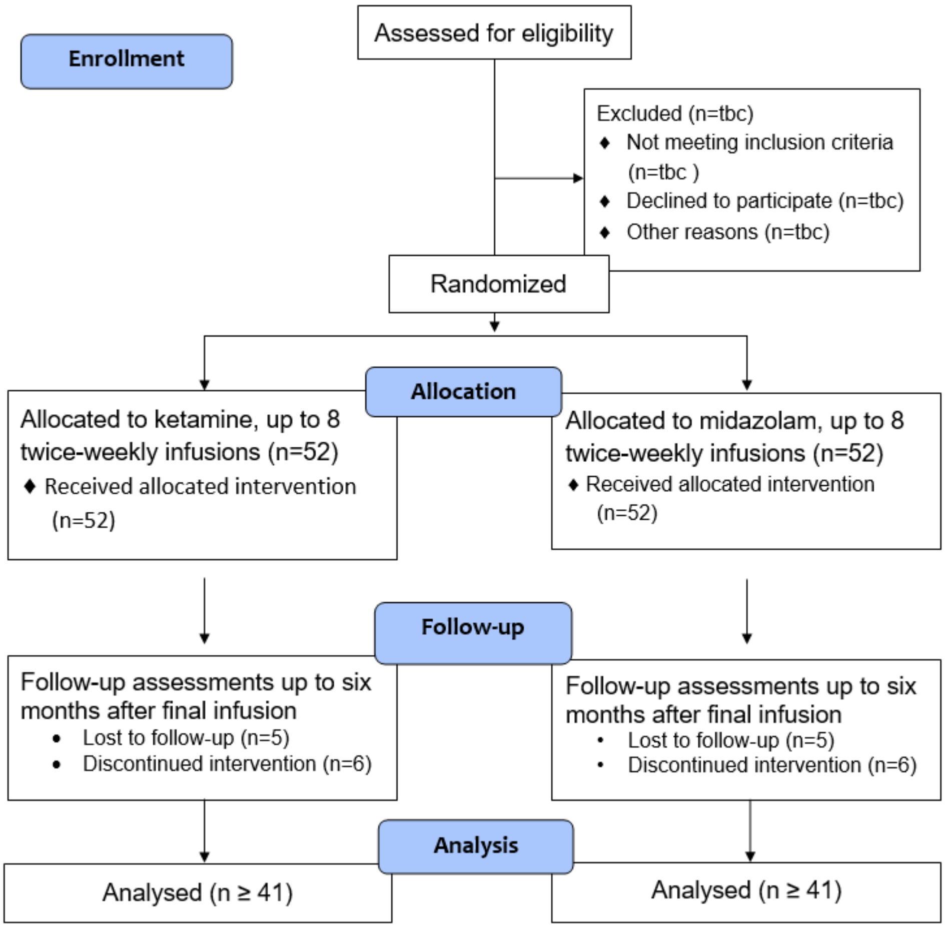

11.2 Selection of study population

11.2.1 Overall description of trial patients

Inpatients will be recruited from St Patrick's University Hospital (241 beds), in central Dublin, and its sister St Patrick's Hospital Lucan (52 beds) located 12 km away in Lucan in suburban/rural west Co. Dublin. Both hospitals are part of the national *St Patrick's Mental Health Services* (https://www.stpatricks.ie/), Ireland's largest single independent provider of mental health care. The Service runs the largest ECT Clinic in the British Isles and has specialist mood disorders programmes in both hospital sites. St Patrick's Hospital Lucan, in particular, focuses on treatment-resistant depression.

Participants recruited from these two hospital sites are broadly representative of the Irish population with moderate or severe depression requiring hospital admission. For example, we recently reported the largest ever European ECT trial (n =138; *EFFECT-Dep,* NCT01907217) recruiting from these two hospitals. Using publicly available national data collected annually by the Mental Health Commission (https://www.mhcirl.ie/), we were able to demonstrate that *EFFECT-Dep Trial* participants were similar in age and gender to the entire background Irish population treated with ECT during the study period [39]. Regarding gender, 63% of the depressed sample were female in

the aforementioned *EFFECT-Dep Trial* and 52% in our completed *KARMA-Dep* pilot trial. We therefore expect the gender balance to be representative of the Irish background population with depression in the larger definitive *KARMA-Dep (2) Trial*.

Baseline clinical and demographic information will be obtained from interview and electronic healthcare records. Patients, assessors, and patients' treating teams will all be blinded to allocation group. Both groups will continue treatment-as-usual. This entails continuing any regular prescribed psychotropic and other medications and allows for medication changes, which will be monitored and recorded at twice-weekly reviews during the infusion period and at follow-up timepoints. Where possible, patients taking any regular benzodiazepines (every day for the past five days) should omit their dose on the morning of infusion sessions (see section 12.7.1). It is appreciated that omission of benzodiazepines may not be possible for all patients. This will be as per the Investigator's discretion. Patients may also avail of psychotherapies, occupational therapy and psychoeducation programmes. This will reflect the conditions under which adjunctive ketamine would be used in routine clinical inpatient practice, thereby enhancing the generalisability and external validity of the trial.

11.2.2 Inclusion criteria

Participants may be male or female, aged ≥18 years, and from a variety of geographical (within Ireland) and socioeconomic backgrounds. To be eligible for inclusion, each participant must meet each of the following criteria at screening (Visit 0) and continue to fulfil these criteria at baseline before the first ketamine/midazolam infusion (Visit 1) as indicated in Table 1 below.

(i) Participants must be voluntary admissions and able and willing to give written informed consent and comply with the requirements of this study protocol.
(ii) Admitted to hospital and diagnosed with major depressive disorder or bipolar disorder (current episode depression), confirmed by Inpatients with a clinical ICD-10 diagnosis of depression on pre-screening must have a confirmed DSM-5 diagnosis of a major depressive episode (unipolar or bipolar) using the Mini International Neuropsychiatric Interview (MINI; updated Version 7 for DSM-5) [40]; and have a Montgomery-Åsberg Rating Scale for Depression (MADRS) [31] score ≥20 at screening and start of the first infusion.
(iii) Female patients of childbearing potential and male patients whose partner is of childbearing potential must be willing to ensure that they or their partner use two contraception methods, including a barrier method, during the randomised treatment phase and for 12 weeks thereafter.
(iv) Female patients' plasma pregnancy test performed at the screening visit must be negative.
(v) Patients have clinically acceptable laboratory and ECG findings during the current admission prior to the first infusion session.

11.2.3 Exclusion criteria

Patients are excluded from the study if any of the following criteria are met at Screening (Visit 0) or at baseline before the first infusion session (Visit 1):

(i) Current involuntary admission
(ii) Patients unable to provide written informed consent
(iii) Patients who have participated in another ketamine study or received any other investigational agent within the past 12 months

(iv) Medical condition rendering unfit for ketamine or midazolam (Ketamine is contraindicated, as per Summary of Product Characteristics, in persons in whom an elevation of blood pressure would constitute a serious hazard. Ketamine should not be used in patients with eclampsia or pre-eclampsia, severe coronary or myocardial disease, C.V.A. or cerebral trauma or if there is hypersensitivity to the active substance. Contraindications to midazolam include known hypersensitivity to benzodiazepines, severe respiratory failure or acute respiratory depression. Bradycardia is a known adverse effect of midazolam and ketamine. Therefore, patients with pre-existing bradycardia will be excluded.)

(v) Currently taking any of the contraindicated medications listed in section 12.7.2

(vi) Active suicidal intention (score of 6 on item 10 (Suicidal Thoughts) in the MADRS)

(vii) Confirmed diagnosis of dementia

(viii) Lifetime history of schizophrenia or schizoaffective disorder; active anorexia nervosa or bulimia nervosa in the past 12 months; alcohol or other substance use disorder (with the exception of nicotine) in the previous six months; any DSM-5 disorder other than a major depressive episode (unipolar or bipolar) as the primary presenting problem

(ix) ECT administered within the last two months

(x) Pregnancy or inability to confirm use of adequate contraception during the trial

(xi) Breastfeeding women

11.3 Study assessments and procedures

Table 1: Schedule of assessments and procedures for the *KARMA-Dep (2) Trial*

		Inpatient								Follow-up		
		Treatment										
**Assessment**	**Screening Visit 0**	**Visit 1**	**Visit 2**	**Visit 3**	**Visit 4**	**Visit 5**	**Visit 6**	**Visit 7**	**Visit 8/Final Infusion Visit**	**Visit 9**	**Visit 10**	**Visit 11**
		**Week 1**		**Week 2**		**Week 3**		**Week 4**		**6 weeks ± 1 week**	**12 weeks ± 2 weeks**	**24 weeks ± 3 weeks**
Informed Consent[1]	X											
Inclusion/Exclus-ion criteria[2]	X	X	X	X	X	X	X	X	X			
Past and current medical conditions	X											
Demographics	X											
MINI, ATRQ	X											
Physical exam[3]	X											
Height, weight, BMI[4]	X	X		X		X		X				
ECG[5]		X	X	X	X	X	X	X	X			
Clinical laboratory assessments[6]	X								X			

Blood pregnancy test[7]	X										
Vital signs[8]		X	X	X	X	X	X	X	X			
Randomisation[9]	X										
Treatment review[10]		X	X	X	X	X	X	X	X	X	X	X
Drug administration[11]		X	X	X	X	X	X	X	X			
MADRS[$]	X	X[$]	X[$]	X[$]	X[$]	X[$]	X[$]	X[$]	X[$]	X	X	X
QIDS-SR_{16}[$]	X	X[$]	X[$]	X[$]	X[$]	X[$]	X[$]	X[$]	X[$]	X	X	X
MoCA[&]	X	X[&]							X[&]		X	X
OAA/S-R[+]		X[+]	X[+]	X[+]	X[+]	X[+]	X[+]	X[+]	X[+]			
CADSS[+]		X[+]	X[+]	X[+]	X[+]	X[+]	X[+]	X[+]	X[+]			
BPRS[+]		X[+]	X[+]	X[+]	X[+]	X[+]	X[+]	X[+]	X[+]			
YMRS[+]		X[+]	X[+]	X[+]	X[+]	X[+]	X[+]	X[+]	X[+]			
PRISE*	X	X*	X*	X*	X*	X*	X*	X*	X*	X	X	X
AE review[12]		X	X	X	X	X	X	X	X	X	X	X
SAE review[13]		X	X	X	X	X	X	X	X	X	X	X
PWC-20[#]	X[#]				X[#]				X[#]	X[#]	X[#]	
Concomitant medication[14]	X	X	X	X	X	X	X	X	X			
Healthcare costs: CSRI[14], medications costs	X									X	X	X
EQ-5D-5L	X									X	X	X
Drug accountability[15]		X	X	X	X	X	X	X	X			
Assessment of patient and rater blinding		X							X			X

Fasting[16]		X	X	X	X	X	X	X	X			

Abbreviations

MINI: The Mini-International Neuropsychiatric Interview, ATRQ: Antidepressant Treatment Response Questionnaire, BMI: Body mass index, ECG: Electrocardiogram, MADRS: Montgomery-Åsberg Rating Scale for Depression, QIDS-SR_{16}: Quick Inventory of Depressive Symptoms, MoCA: Montreal Cognitive Assessment, OAA/S-R: Observer's Assessment of Alertness/Sedation Scale - Responsiveness Subscale, CADSS: Clinician-administered Dissociative States Scale, BPRS: Brief Psychiatric Rating Scale, YMRS: Young Mania Rating Scale, PRISE: Patient-Rated Inventory of Side Effects, AE: Adverse event, SAE: Serious adverse event, PWC-20: 20-item Physician Withdrawal Checklist, CSRI: Client service Receipt Inventory, EQ-5D-5L: EuroQol-5 dimensions-5 level scale for health status.

[$] These assessments will be completed at screening (prior to randomisation), before and after all infusion sessions (at the following timepoints: -40 (±20) mins before the infusion begins; +60 (±10) mins and +120 (±10) mins after the infusion begins; +24 (±1) hours after the infusion ends) and at the 6, 12 and 24 weeks follow-up time points. Scores on sleep and appetite items including items 4 and 5 on the MADRS, and items 1,2,3,4,6,7,8,9 on the QIDS-SR_{16} will be carried over from -40 minutes before each infusion to +60 minutes, +120 minutes, and +24 hours after each infusion.
[&] MoCA will be completed at screening (prior to randomisation), at +120 (±10) mins after the infusion begins during the first and eighth/final infusion sessions (Visits 1 and 8), and at the 12 and 24 weeks follow-up time points.
[+] These assessments will be completed three times at each infusion session: before (-30 (±10) mins before the infusion begins), during (+30 (±5) mins after the infusion begins) and after (+60 (±10) mins after the infusion begins, i.e. 20 (±10) mins after the infusion ends).
* PRISE will be completed at screening (prior to randomisation), after all infusion sessions (+120 (±10) mins after the infusion begins), and at the 6, 12 and 24 weeks follow-up time points.
[#] PWC-20 will be completed at screening (Visit 0), 24 (±1) hours after the end of the fourth and final/eighth infusions, and the 6- and 12-week follow-up timepoints.

1. Informed consent will be obtained prior to any study-related procedures being undertaken. Informed consent will be taken no later than 10 days after admission to hospital.
2. Inclusion/Exclusion criteria will be assessed at screening and then again at every visit before dosing is carried out.

3. Physical Examination on admission will be reviewed at screening. During all other visits a physical exam can be performed if clinically indicated by the PI.
4. Weight (kg), height (cm), BMI (kg/m²). Height will be measured at screening only. BMI is a derived variable automatically calculated by eCRF using recorded weight and height. Weekly weight will be recorded.
5. A 12-lead ECG will be completed during the current admission and reviewed by the Anaesthetist before the first infusion (Visit 1). Continuous EEG (single lead) monitoring will occur during all infusions (0 to +40 mins after the infusion begins) and up to +120 minutes after the infusion begins, and longer if requested by the Anaesthetist, to measure haemodynamic change.
6. Clinical laboratory assessments will include all routine biochemistry and haematology blood tests performed on admission. These will also be reviewed by the Anaesthetist before the first infusion (Visit 1). Liver function tests will be repeated and reviewed after the final infusion session.
7. Blood pregnancy test to be carried out in women of childbearing potential at visit 0.
8. Heart rate, blood pressure and pulse oximetry will be recorded before, during (0 to +40 mins after the infusion begins) infusions and for up to +120 minutes after the infusion begins to measure haemodynamic changes.
9. Randomised allocation will only take place once all eligibility criteria are met at the screening visit.
10. At each visit, before drug administration is carried out, a treatment review will be completed to ensure the patient is still eligible and the correct dose will be administered.
11. Administration of either Ketamine (0.5 mg/kg; Pfizer Healthcare Ireland) or midazolam (0.045 mg/kg; Roche Products Ireland Ltd) will be made up as colourless saline solutions and administered over 40-minutes using an infusion pump to deliver the required total amount of ketamine/midazolam as per individual body weight.
12. AEs will be reviewed at every visit. All AEs must be reported and followed up until final visit.
13. SAEs are reviewed at every visit. All SAEs must be reported appropriately and followed up until resolved.
14. Concomitant medication: All over-the-counter or prescription medication, vitamins, herbal supplements, and any other therapies will be documented on the CSRI from the previous month prior to Visit 0. At all other visits, concomitant medication taken since last visit will be documented. Adherence to prohibited medication requirements must be assessed and documented.
15. Drug accountability will be completed after each infusion session.
16. Patients will be asked to fast >3 hours prior to the morning infusion clinics. However, if taking cardiovascular medications, these should be taken with a sip of water. Where possible, patients taking any regular benzodiazepines (every day for the past five days) should omit their dose on the morning of infusion sessions. Omission of benzodiazepines may not be possible for all patients; this will be as per the Investigator’s discretion.

11.3.1 Protocol Deviations

Intentional or prospective deviations from the protocol are not allowed unless when necessary to eliminate an immediate hazard to patients. The PI is responsible for complying with all protocol requirements and applicable local laws regarding protocol deviations. If a major protocol deviation occurs after a patient has been enrolled, the PI is responsible for notifying the study sponsor by e-mail to clinicaltrialsponsorship@tcd.ie and, if applicable, the Research Ethics Committee (REC) and the HPRA.

Protocol deviations will include: inclusion or exclusion criteria not satisfied, deviations related to study drug administration, deviations to the schedule of visits and procedures, or deviations related to nonpermitted concomitant medications.

Informed consent will be obtained prior to any study-related procedures being undertaken.

11.3.2 Description of Study Assessments

11.3.2.1 Diagnosis and treatment history

Diagnosis of a major depressive episode (DSM-5) will be confirmed by the structured diagnostic *Mini International Neuropsychiatric Interview* (MINI) [40]. It is likely that most inpatient participants will have treatment-resistant depression. The presence of treatment-resistant depression will be ascertained using the Antidepressant Treatment Response Questionnaire [41].

11.3.2.2 Medical and Surgical History

Details of current and previous diagnoses and treatments will be recorded.

11.3.2.3 Demographics & Personal and Psychiatric History

Additional demographic and baseline clinical data from patient interview and case-note review will include: age, sex; occupation, years in education, educational attainment; duration of index depressive episode, number of previous depressive episodes, age at first depressive episode; presence of psychotic symptoms (also detected by the MINI); personal and family psychiatric history, including alcohol/substance dependence; and current medications and other therapies.

11.3.2.4 Physical Examination

The complete physical examination will include the evaluation of the cardiovascular, dermatological, musculoskeletal, respiratory, gastrointestinal, neurological systems. Height, weight and temperature will also be recorded.

11.3.2.5 Vital Signs

Vital signs will not be recorded for the purposes of the trial outside of infusion clinics. During infusion clinics participants' heart rate, pulse oximetry and blood pressure will be monitored. before and during infusions and up to +120 minutes once the infusion has begun. Heart rate and pulse oximetry will be continuously monitored before and during infusions and up to +120 minutes once the infusion has begun; these will be recorded at the same times as for blood pressure. Blood pressure will be measured and recorded before infusions begin, every 10 (±3)

minutes during the infusion (+10, +20, +30 and +40 mins after the infusion has begun) and at +60(±5), +90(±10) and +120(±10) minutes after the infusion has begun.

11.3.2.6 12-Lead Electrocardiogram (ECG) Test

A resting 12-lead ECG, obtained routinely during the current admission before the first infusion session, will be reviewed by the investigator at screening. ECG monitoring will be performed for all participants at infusion sessions, before and after administration of the IMP as described just above in section 11.3.2.5. Abnormal findings will be noted for clinical significance, and the report will be signed by the investigator.

11.3.2.7 Clinical Laboratory Tests

Clinical laboratory test results will be recorded as part of this trial. All participants will have had recent routine laboratory investigations (i.e. full blood count, renal, liver and thyroid function tests) at admission. These will be examined by the PI or delegated sub-investigator at screening and also by the Anaesthetist prior to the first infusion clinic to help ensure the patient is medically stable. Liver function tests will be repeated after the final infusion session.

11.3.2.8 Pregnancy Tests

Women of childbearing potential who are willing to participate in the study and give consent are requested to inform researchers if there is any possibility they may be pregnant. For all women of childbearing potential, a blood serum pregnancy test will be performed at screening (Visit 0) before commencing study drug dosing. The results must be available, documented and negative before the first dose of study drug is administered. Date of last menstrual period (LMP) will be documented at the first infusion clinic. Information relating to the importance of contraception during the trial is provided in the Participant Information Leaflet.

11.3.2.9 Prior and Concomitant Medication

All over-the-counter or prescription medication, vitamins, herbal supplements, and any other therapies will be recorded on the CSRI in the previous month. In a direct export from the participant's electronic health record, all over-the-counter or prescription medication, vitamins, herbal supplements, and any other therapies will be recorded from the time of consent until the final infusion visit Contraindicated medications are listed in this Protocol (see section 12.7.2). Changes in medications and therapies will be monitored and documented at all randomised treatment assessment time points (see Table 1). At long-term follow-ups (Visit 9, 10 and 11), self-reported medications will be recorded on the CSRI.

11.3.2.10 Psychiatric Assessments

The *Mini-International Neuropsychiatric Interview* (MINI V7.0) will be performed by trained researchers at baseline assessment. MINI is a structured interview that is diagnostic for disorders in DSM-5 and ICD-10 and will be used in this study to confirm a diagnosis of major depressive episode due to either unipolar major depressive disorder or bipolar disorder [40].

The *Montgomery-Åsberg Depression Rating Scale* (MADRS) [31] is a validated depression rating measure that will be performed on a repeated basis by trained researchers at various points throughout the trial as a measure of depressive symptomatology and response to treatment. Sleep and appetite scores from items 4 and 5 will be carried over from -40 minutes before

each infusion to +60 minutes, +120 minutes, and 24 hours after each infusion in line with the methodology of previous literature [32-35].

The *Quick Inventory of Depressive Symptoms, Self-Report*(QIDS-SR_{16}) [42] is a validated self-report measure of depressive symptoms. It is best practice to include both self- and clinician-rated measures of depressive symptoms to assess treatment response in depression. Sleep and appetite items 1,2,3,4,6,7,8 & 9 on the QIDS-SR_{16} will be carried over from -40 minutes before each infusion to +60 minutes, +120 minutes & 24 hours after each infusion in line with the methodology of previous literature [32-35].

11.3.2.11 Cognitive Assessments

Cognitive measures will include the *Montreal Cognitive Assessment* (MoCA) [43], which will be performed on a repeated basis by trained researchers at various points throughout the trial. This validated cognitive measure has parallel versions for repeated use to minimise practice effects and provides a standardised measure of global cognition and includes measures of executive function.

11.3.2.12 Safety and Tolerability Measures

During infusion sessions, adverse or psychotomimetic effects of either ketamine or midazolam will be monitored using validated scales comprising: *Observer's Assessment of Alertness/Sedation Scale - Responsiveness Subscale* (OAA/S-R) [44] *Clinician-Administered Dissociative States Scale* (CADSS) [23]; *Brief Psychiatric Rating Scale* (BPRS) [45]; *Young Mania Rating Scale* [46]; and the *Patient-Rated Inventory of Side Effects* (PRISE) [47]. These will be administered before, during and after infusions in order to capture the range of possible subjective and objective side effects of either agent.

11.3.3 Efficacy Assessment

The primary outcome will be the change from baseline in the objectively rated Montgomery-Åsberg Rating Scale for Depression (MADRS) [31] score 24 hours after the final infusion. To enter the study patients must score ≥20 at screening and start of the first infusion.

Response to treatment is defined as a ≥50% improvement from baseline MADRS score. *Remission* is defined as achieving a MADRS score ≤10. For those deemed to be treatment "remitters", *relapse* at follow-up time points is defined as a MADRS score of ≥18. Hospital re-admission and deliberate self-harm/suicide also constitute relapse and timing of such events will be recorded.

MADRS mood ratings (see Table 1) will be repeated at weeks 6, 12 and 24 during the follow-up period; subjective mood will also be rated by patients using the Quick Inventory of Depressive Symptoms, self-report version (QIDS-SR_{16}) [48].

11.3.4 Safety Assessment

The following safety assessments are being used to collect information as safety endpoints related to the IMPs. These will be exempted from reporting in the CRF as AE's. However, if they come to fulfil the SAE criteria (see section 13.1.3 below) they will be reported to pharmacovigilance as such.

(i) Psychotomimetic and dissociative symptoms: Acute psychotomimetic effects of ketamine are usually short-lived and restricted to the infusion period, resolving within one hour. They include dissociative and psychiatric symptoms. In line with previous ketamine trials [1, 12, 24], we will perform the following assessments three times at each infusion session [before the infusion begins (-30 mins), during (+30 mins after the infusion begins) and after (+60 mins after the infusion begins)]:

- *Dissociative effects*: the *Clinician-Administered Dissociative States Scale* (CADSS) is a 23-item questionnaire for administration by raters for assessment of present-moment dissociative symptoms [23]. The scale assesses for dissociative symptoms in identity, proprioception, time perception, colour and depth perception, and other modalities. An open prompt question for each item is asked, e.g. "Do things seem to be moving in slow motion?" and if answered positively, is followed by a series of further questions to ascertain the severity of the symptom. Each item is scored 0-4 according to detailed instructions in the CADSS manual.
- *Psychotomimetic effects*: the *Brief Psychiatric Rating Scale* (BPRS) is an 18-item questionnaire assessing a wide variety of symptoms of psychotic disorders [49]. It is an observer-administered scale and incorporates patient self-report and clinician observation. Following the initial 16-item scale, two items were added and an interview guide developed. We will be using the four-item psychosis subscale to assess for possible psychotomimetic effects of ketamine. This subscale assesses domains of unusual thought content, suspiciousness, bizarre behaviour and hallucination. Each item is scored from 1-7 (very mild to extremely severe); thus in the four-item subscale, respondents can score a minimum of zero. The manual provides prompt questions for each item and a detailed scoring system for various responses.
- *Mood elevation*: the *Young Mania Rating Scale* (YMRS) is a commonly used observer-rated assessment tool for severity of mania and hypomania [50]. The full scale consists of several items that assess for emotional, biological and cognitive symptoms of mania and hypomania. Inter-rater reliability has been reported as 0.93 and it has good concurrent validity with other mania scales [50]. In the present study, the single mood item, rated 0-4, representing a range from normal mood to frank euphoria, will be used to assess for possible mood elevation during the infusion sessions.

(ii) Cognitive effects: Ketamine in healthy volunteers can cause transient impairment in working and episodic memory, procedural and semantic memory, executive function, verbal fluency, and verbal memory, resolving shortly after the infusion [51]. Procognitive effects of repeated doses of ketamine have been suggested in some studies. The following validated instrument will be used to assess cognitive outcomes during the trial: at baseline (prior to randomisation), at +120 mins after the infusion begins during the first and final infusion sessions, and at the 12 and 24 weeks follow-up time points (Table 1).

- The *Montreal Cognitive Assessment* (MoCA) was designed as a rapid (10 mins) screening instrument for mild cognitive dysfunction. The test-retest reliability has been reported as 0.92 and concurrent validity when compared to the Mini Mental State Examination was r= 0.87. There are parallel versions to avoid practice effects and it is regularly updated. For example, a recent development is the Memory Index Score. It assesses different cognitive domains: attention and concentration, executive functions, memory, language, visuoconstructional skills, conceptual thinking,

calculations, and orientation. The raw scores can also be extracted to generate functional “Index Scores” for orientation, attention, memory, executive, visuospatial, and language functions [52].

(iii) Physical safety and tolerability: In a recent review, the most common adverse events of single ketamine infusions were drowsiness, dizziness, incoordination, blurred vision, and dissociative symptoms, with only 1.95% of 205 infusions discontinued [53]. Approximately 33% of participants experienced mild haemodynamic changes (systolic or diastolic blood pressure (BP)>180/100 or >20% increase above pre-infusion reading or tachycardia >110 beats/min). Safety and tolerability will be monitored throughout all infusion sessions and during follow-up as relevant, as we have previously described [24]:

- Physical side-effects: heart rate, blood pressure, oximetry, ECG. As noted above, infusions will be discontinued by the Anaesthetist if there are persisting non-physiological haemodynamic changes (i.e. heart rate >110/minute or systolic/diastolic blood pressure >180/100 for more than 15 minutes) that do not respond to beta-blocker therapy.
- The *Observer’s Assessment of Alertness/Sedation Scale - Responsiveness Subscale* (OAA/S-R) [44] is a simple validated observer-rated assessment that will be used to measure levels of sedation experienced by participants during infusions. The *Responsiveness* subscale of the OAA/S ranges in score from 1 to 5, where 1 denotes deep sleep (i.e. no response to mild prodding or shaking) and 5 denotes fully alert. The OAA/S-R will be administered before (-30mins), during (+30mins) and after (+60mins) each of the twice-weekly infusions.
- The *Patient-Rated Inventory of Side Effects* (PRISE) [47] will be used to systematically enquire for both global and specific symptoms, irrespective of their being treatment-related or not. Any adverse events are coded from a list of possible events and also coded as being” tolerable” or “distressing”. The inventory includes systematic enquiries about neurological (e.g. headache, dizziness, blurring of vision, tremors), urinary (e.g. dysuria, difficulty urinating, frequency), fatigue, and anxiety symptoms. PRISE will be completed at screening (prior to randomisation), after all infusion sessions (at +120 mins after the infusion begins), and at the 6, 12 and 24 weeks follow-up time points.

(iv) Withdrawal effects: No evidence of a withdrawal syndrome was detected after a course of repeated intranasal esketamine in recent large trials [5, 54, 55] and a Cochrane meta-analysis of ketamine, esketamine and other glutamate receptor modulators [56]. There is no known risk of a withdrawal syndrome in the published ketamine literature to date, though there are theoretical concerns about addiction potential [57, 58]. We will therefore monitor for features of a withdrawal syndrome at screening (Visit 0), 24 hours after the fourth and final infusions, and the 6- and 12-week follow-up timepoints.

- The *20-item Physician Withdrawal Checklist* (PWC-20) will be used to assess for potential withdrawal symptoms during after completing the allocated course of ketamine/midazolam infusions [59]. The PWC-20 is a brief easy-to-use instrument and has been reported to have good validity, internal consistency, test-retest and interrater reliability.

All adverse medical, psychotomimetic and general events will be reported to the Trial Steering and Data Monitoring Committees.

11.3.5 Economic and Quality Of Life Analyses

Treatment costs will be collected and other healthcare costs estimated using a version the *Client Service Receipt Inventory* (CSRI) adapted for a recent antidepressant trial and cost-effectiveness study [60]. Health-related quality-of-life will be measured using the EQ-5D-5L (Five-level version of the EuroQol five-dimensional questionnaire) [61].

The aim of the economic analysis is to ascertain the relative value for money of ketamine used as an adjunctive therapy to routine care. The study will adopt an intention-to-treat design. All patients meeting the inclusion criteria and recruited to the study will be included in these analyses. Healthcare utilization will be captured at baseline and three follow-up visits. The service use instrument will be adapted to relate to appropriate time intervals. It will focus on the preceding month at the initial baseline assessment and in the interval since the last assessment visit at each subsequent data capture point (week 6, week 12 and week 24). Services specifically identified will include GP visits, ER visits, inpatient stays, outpatient visits and use of community and social services including psychotherapy. Patients will be asked to bring their currently prescribed medicines and/or a recent prescription with them at week 6, week 12, and week 24 assessments so that these can be recorded and costed using the MIMS pricing system. Other service use will be monetised using published references for primary and community care, Hospital In-Patient Enquiry (HIPE; https://www.hiqa.ie/areas-we-work/health-information/data-collections/hospital-patient-enquiry-hipe) for inpatient services, and from hospital pharmacy costs in respect of ketamine and other hospital prescribed medicines. Healthcare costs will be calculated from the perspective of a publicly funded third party payer. In addition to disease specific measures, in particular the MADRS depression score, the EQ-5D-5L descriptive system will be used to capture data on health-related quality of life.

Cost-effectiveness and cost-utility analyses will be undertaken. Incremental costs will be based on the cumulative difference in costs between the intervention and control groups. That is, the difference in the value of resources used during the course of the trial. Incremental effects will be estimated as the difference in the change in mental health between the intervention and control groups. That is, the difference in the change in mental health between the two groups calculated as the difference in the area under the curves of the intervention and control groups for each health outcome during the course of the trial. A series of incremental cost-effectiveness ratios (ICERs) will be estimated based on a bootstrapping exercise to allow for the joint dependence of costs and effects. The ICERs will estimate the cost per additional unit difference in health based on each instrument, following 1000 bootstrapped estimates of the trial sample. No discounting will be required in the case of cost-effectiveness analyses as the trial for each individual will be completed within the course of one year.

A cost-utility analysis will be undertaken in a similar fashion. Utility scores using the Irish EQ-5D-5L value set will be applied to ED-5D-5L data collected at each time point to estimate a series of health utility indices. Combined with any difference in longevity between the study groups observed over the course of the study, differences in quality adjusted life year estimates will be made. This will be related to costs in an incremental cost-utility ratio

(ICUR). As with the ICERs, these will be based on a bootstrapping exercise using trial data. As with ICERs, estimates will be for the observation period of the study, which being less than one year, will not necessitate reference to discounting. Cost-effectiveness and cost-utility analyses will be undertaken mindful of the greater sensitivity of disease specific measures and of the need for generic health-related quality of life measures to assess relative value for money.

While a publicly funded healthcare perspective will be adopted in the main analyses, it is recognised that significant costs may arise related to, for example, lost production on the part of the patient and their carer. The resource use instrument will therefore include an adaptation to capture information on episodes of absence from work for the periods covered at data capture points (absenteeism) and estimates of relative productivity (presenteeism) for the same intervals where actual absences from work did not arise.

Sampling uncertainty for estimated costs and effectiveness parameters will be undertaken together with the impact of methodological assumptions related, for example, to the perspective of the study.

11.3.6 Timing of study procedures

Table 1 provides a summary of timings of study procedures. Where additional study visits are clinically required an unscheduled visit will be performed. A separate unscheduled eCRF visit will be completed to record these visits.

For early withdrawal, the End of Study Form will be completed. In addition, the End of Study Form will be completed detailing the reason for patient withdrawal where given. The reason for withdrawal will also be recorded in the source documents.

11.3.7 Screening procedure

When potential trial patients are admitted for treatment of a depressive episode they will be provided with information about the study by an investigator. If agreeable to participate, informed consent will be obtained prior to any study related procedures being undertaken. The initial MADRS and MINI assessment will be performed to ensure further eligibility criteria are met. All screening, background data collection and baseline assessments will be completed before the first infusion begins as outlined in Table 1.

11.3.8 Screening baseline assessments

The following pre-treatment Baseline assessments will be performed prior to randomisation:

- confirmation of eligibility (review inclusion/exclusion criteria)
- recording of demographics, medical history, personal history and concomitant medications
- physical examination
- Evaluation of ECG test obtained during the current admission prior to the first infusion
- Evaluation of routine biochemistry and haematology results
- for women of childbearing potential a negative pregnancy test must be performed and documented
- Mini-International Neuropsychiatric Interview (MINI)
- Montgomery-Åsberg Depression Rating Scale (MADRS)
- Antidepressant Treatment Response Questionnaire (ATRQ)
- Montreal Cognitive Assessment (MoCA)

- Quick Inventory of Depressive Symptoms – Self-Rated ($QIDS\text{-}SR_{16}$)
- Client Service Receipt Inventory (CSRI)
- EQ-5D-5L

11.3.9 Subsequent study visits and procedures

The timetable for study visits and procedures is presented in Table 1. In line with the pragmatic trial design [37], patient's will undergo usual inpatient care as prescribed by their treating team during the admission for treatment of the index acute depressive episode. Following the baseline assessment above, patients will be assessed twice-weekly during and after allocated infusion sessions using the MADRS and $QIDS\text{-}SR_{16}$.

Patients must score ≥20 on the MADRS at baseline (Visit 0) and before the first infusion session (Visit 1) to be eligible for the study. Following randomisation and prior to the commencement of the randomised treatment phase, mood, and safety outcomes will be assessed as described in sections 11.3.3 and 11.3.4 above. Concomitant medications will be reviewed before each infusion session to monitor changes in medications. Randomisation will take place as detailed below (see section 11.3.10). Participants will be inpatients in the hospital for the eight infusion sessions at which time they will receive an infusion of either ketamine or midazolam. Patients will be asked to fast for at least three hours prior to morning infusion clinics. Where possible, patients taking any regular benzodiazepines (every day for the past five days) should omit their dose on the morning of infusion sessions (see section 12.7.1). It is appreciated that omission of benzodiazepines may not be possible for all patients. This will be as per the Investigators discretion and documented in the patient notes. Pre-infusion monitoring will be performed during the one hour before infusions begin and the infusions will take place over 40 minutes (0 to +40 mins) as detailed below. Ongoing monitoring of safety and tolerability outcomes will continue for +120 minutes after the infusion begins. Each patient will be assessed 24 hours after each infusion session to check for potential adverse effects and assess mood outcomes.

Ketamine (as hydrochloride; 0.5 mg/kg; Pfizer Healthcare Ireland)) and midazolam (as hydrochloride; 0.045 mg/kg; Roche Products Ireland Ltd) will be made up as colourless saline solutions and administered as slow infusions over 40 minutes using an infusion pump, as per previous similar studies, to deliver the required dosage based on body weight [25].

The drugs will be securely stored in the Hospital Pharmacy and made up for use by the consultant Anaesthetist on the mornings when infusions will be given and double checked by a staff pharmacist not otherwise involved in the trial, using the St Patrick's University Hospital Ketamine Research Clinic as the treatment facility. Like ketamine at 0.5 mg/kg, midazolam at 0.045 mg/kg has anaesthetic effects and causes some sedation and disorientation with a similar time course and adverse effect profile. In subanaesthetic doses, ketamine is a safe drug but can cause transient rises in pulse and blood pressure during infusion and for up to 80 minutes afterward. All patients will therefore be monitored for heart rate, blood pressure, pulse oximetry, and ECG before and during infusions and for up to +120 minutes after the infusion begins. Infusions will be discontinued if there are persisting non-physiological haemodynamic changes (i.e. heart rate >110/minute or systolic/diastolic blood pressure >180/100 for more than 15 minutes) that do not respond to beta-blocker therapy. Such events will also be discussed with the PI and reported to the Sponsor.

Assessments including cognitive and mood assessment will be performed as outlined in section 11.3.3 and 11.3.4 above, before, during and after infusions.

Patients will be withdrawn from the trial if: (i) an infusion is discontinued for the above haemodynamic reasons or other serious medical contra-indications, e.g. over-sedation, hypoxia, intolerable adverse physical reactions; (ii) the patient develops mania or psychosis; (iii) the patient becomes severely depressed and/or suicidal. The PI will assess the details of the withdrawal and the Sponsor will be notified.

To ensure patient safety, all infusions will take place while an in-patient. Patients will be advised not to drive or operate heavy machinery for 24-hours post-infusions and will be contacted by the researcher 24-hours after each infusion to enquire about side-effects and adverse events.

Participants will be followed up over 24 weeks to assess for relapse. Follow-up mood, safety outcome and health economics assessments as well as recording of concomitant medications will be repeated at weeks 6, 12 and 24 as outlined in Table 1. These assessments will take place in person, either at the site or by telephone or by video conference. Where in-person follow-up appointments are not possible, assessments can take place over the telephone/by video conference where practicable. Reasonable meal and travel expenses incurred by participants attending for follow-up appointments will be reimbursed.

At each visit, participants will be asked if they have experienced any AEs/SAEs since last visit.

11.3.10 Method of assigning Patients to treatment groups

Participants will be recruited at admission for treatment of a depressive episode. Those who meet eligibility criteria and are willing to participate in the study, will be invited to be randomly assigned to one of two treatment groups in a 1:1 ratio.

11.3.10.1 Randomisation

Random allocation, using randomly permuted blocks will be done independently by the Centre for Support and Training in Analysis and Research (CSTAR, University College Dublin, www.cstar.ie). This will be done independently of the Trial Statistician. To ensure allocation concealment, allocation information will be provided in a randomisation list available only to the Anaesthetist and trial Pharmacist and delivered by secure mail. This will be stored in a in the locked Clinic office within a locked box to which only the Anaesthetist has the key. A matching set of opaque randomisation envelopes will also be provided by CSTAR, stored in a locked drawer in the Assistant Director of Nursing's locked office in St Patrick's University Hospital, to be accessed by clinical staff in the event of emergency unblinding. This system has been successfully piloted in previous ketamine trials at St Patrick's University Hospital (e.g. NCT02414932) [24]. Pharmacovigilance in SJH-CRF will also have a matching set of opaque randomisation envelopes for emergency unblinding.

11.3.10.2 Blinding

Study treatment assignment will be blinded for both the investigators and the trial participants. To ensure patient safety during infusions and in the post-infusion period, the Anaesthetist administering the ketamine/midazolam infusions will not be blinded but will not be involved in outcome assessments or data analysis. Infusions will be prepared by the Anaesthetist in a location separate to the infusion area and labelled as per Annex 13 (*EU Guidelines to Good Manufacturing Practice, Medicinal Products for Human and Veterinary Use,*

Annex 13, Investigational Medicinal Products) prior to transfer to the infusion area. Success of blinding for patients and raters will be assessed by asking them to guess the allocated treatment after the first and final treatments and at the end of the 24-week follow-up.

The matching set of envelopes containing allocation information will remain unopened but may be used where emergency unblinding is indicated where, in the opinion of the investigator or other physician, it is necessary in order to assess and/or treat an adverse event. Unblinding for one or all participants will take place if it is in the best interests of the participants in order to assess and treat an adverse event. In the case of an emergency, when knowledge of the treatment assignment is essential for the clinical management of the patient, any investigator may unblind a single patient.

In the event of a potential SUSAR, unblinding will occur for regulatory purposes. A set of allocation information envelopes will also be held in the CRF-SJH to facilitate potential SUSAR unblinding for regulatory reporting. It may therefore be possible to keep the PI and study personnel blinded (refer to section 13.2.5).

Circumstances in which unblinding for multiple or all patients may take place include: multiple SAEs or SUSARs, new information regarding safety of the investigational medicinal products, and unsatisfactory progression of the trial as decided by the DMC.

Any breaking of the blind, whether intentional or unintentional, will be recorded and reported to the Sponsor as soon as possible by e-mail to clinicaltrialsponsorship@tcd.ie. Unblinding for multiple or all patients will be discussed by the Trial Steering Committee at the next meeting. Unblinding will be recorded and justified in the final report.

Provisions to ensure the integrity of the study blind include:

- Provisioned drug product and placebo being identical in colour, dosing method, and appearance
- All secondary packaging during administration being identical between groups (other than study randomization number)
- No biological tests being completed during the study that risk inadvertent unblinding to study treatment allocation

11.4 Definition of end-of-trial

End-of-trial is defined as the final follow-up visit/telephone or video conference assessment of the last participant. End-of-trial will be reported to the Research Ethics Committee (REC), Trial Steering Committee (TSC), Data Monitoring Committee (DMC) and HPRA within 90 days, or 15 days if the study is terminated prematurely. In the event of premature termination, the investigators will inform patients and ensure that the appropriate follow-up is arranged for all involved. A summary report of the study will be provided to the REC, Sponsor and HPRA within 12 months of end-of-trial.

The end-of-study visit form will include:
- Assessment of endpoints i.e. clinical (MADRS, QIDS-SR_{16}) and cognitive (MoCA) outcomes
- Assessment of safety - check for any adverse effects plus the safety assessments outlined in section 11.3.4
- Recording of concomitant medication

The Sponsors and/or the trial steering committee (TSC) have the right at any time to terminate the study for clinical or administrative reasons.

11.4.1 Premature termination of the study

The Sponsor and/or the TSC have the right at any time to terminate the study for clinical or administrative reasons. The DMC may request that the trial be prematurely terminated and this request will be discussed in a timely manner by the TSC.

Premature termination of the trial may take place in the event of the following:

(i) New information regarding safety of investigational medicinal products
(ii) Multiple SAEs or SUSARs
(iii) Unsatisfactory progression of the trial
(iv) Major breach of data confidentiality
(v) Any situation in which premature termination of the trial is judged by the investigators and/or Sponsor to be in the best interests of trial participants

Premature termination of the trial will be reported to the REC, TSC, DMC, HPRA, and Sponsor and will be justified in the Final Report.

11.5 Discontinuation/withdrawal of patients from study protocol

Patients have the right to voluntarily discontinue study treatment or withdraw from the study at any time for any reason without any consequences. The investigator has the right to discontinue a patient from study treatment or withdraw a patient from the study at any time if it is in the best interest of the patient.

Patients must discontinue the investigational medicinal product(s) and be withdrawn from the study for any of the following reasons:

- withdrawal of consent by the patient
- any medical condition that the investigator or Sponsor determines may jeopardize the patient’s safety if she or he continues receiving the study treatment
- premature termination of the trial
- pregnancy
- ineligibility (either arising during the study or retrospectively having been overlooked at screening)
- an adverse event or SAE which requires discontinuation of the study medication
- treatment failure and disease progression
- lack of compliance with the study and/or study procedures (e.g., attending infusions sessions, assessment visits).

- lost to follow-up - at least 3 documented attempts will be made to contact any patient lost to follow-up.
- Inability to remain under medical observation for the duration of the study
- Discontinuation of the study by the Sponsor
- Regulatory authority decision or change in Research Ethics Committee opinion for drug safety problems
- Any other situation where, in the opinion of the investigator or the Sponsor, continuation of the study would not be in the interest of the patient

Ideally, all patients who discontinue should comply with specified follow-up procedures as detailed in this protocol, e.g. assessment via interview comprising clinical and cognitive measures. The only exception to this requirement is when a patient withdraws consent for all study procedures. There is no mandatory physical health monitoring to be performed in the event of a patient withdrawing consent after a complete infusion session (40 mins), including post-infusion monitoring for up to +120 mins after the infusion begins, or between infusion sessions.

However, in the event that consent is withdrawn during an infusion session, monitoring of vital signs and mental health will be performed for +120 minutes following the beginning of the infusion.

If a patient is withdrawn before completing the study, the reason for withdrawal will be entered on the appropriate CRF page. If a patient is withdrawn due to an adverse event, the investigator will arrange for follow-up visits until the adverse event has resolved or until final visit.

12 TREATMENT OF TRIAL PATIENTS

12.1 Description of study treatment(s)

Investigational Medicinal Product: Ketamine: Ketalar 10mg/ml Solution for Injection/Infusion, Pfizer Ireland; 0.5mg/kg of body weight.

Active comparator: Midazolam: Hypnovel 10mg/5ml solution for injection, Roche Pharmaceuticals Ireland; 0.045 mg/kg of body weight.

Both made up as colourless saline solutions and administered over 40-minutes using an infusion pump to deliver the required total amount of ketamine/midazolam as per individual body weight, in a course of up to eight infusions twice-weekly over four weeks.

12.2 Formulation, packaging and handling

Commercial labels will remain on both study treatments. These detail the component, date of expiry and manufacturer. Additional labelling will be attached to each unit of product in accordance with Annex 13.

Clinical Trial labels will be added by Pharmacy staff to each vial of the treatment in St Patrick's University Hospital Pharmacy, in accordance with Annex 13 (*EU Guidelines to Good Manufacturing Practice, Medicinal Products for Human and Veterinary Use, Annex 13, Investigational Medicinal Products*). The saline solution or drug solution will also be labelled in accordance with Annex 13.

Suppliers:
Uniphar Group, 4045 Kingswood Road, Citywest Business Park, Co Dublin.

Manufacturers:
Ketamine: Pfizer Healthcare Ireland, 9 Riverwalk, Citywest Business Campus, Dublin 24
Midazolam: Roche Products (Ireland) Ltd., 3004 Lake Drive, Citywest, Naas Rd., Dublin 24

Pharmacy performing clinical trial labelling:
St Patrick's University Hospital Pharmacy, James St, Dublin 8

12.3 Storage and disposition of study treatment(s)

The treatments will be stored securely in a clean dry area of the Pharmacy Department at St Patrick's University Hospital as per local regulatory requirements. The IMPs used for this study, ketamine and midazolam, are for investigational use only and are only to be used within the context of this study. Both IMPs will remain segregated from pharmacy stock once they are labelled as clinical trial stock until use or destruction.

Products will be prepared into infusions in the clinic room of the Ketamine Research Clinic by the Consultant Anaesthetist who will administer them. The Anaesthetist will be unblinded throughout the trial and patients and raters will remain in a separate area of the Research Clinic for infusions and assessments. Once made up as colourless solutions, infusions will begin within one hour of preparation.

Any unused product will be disposed of according to the study specific Pharmacy Manual and SOPs specified by the Pharmacy for destruction of unused pharmaceutical products.

The study treatment will be stored at St Patrick's University Hospital Pharmacy Dept. Temperatures in the storage area of the Pharmacy are monitored constantly by electronic thermostat and a printed record is available. An alarm process is instigated if the temperature varies from the specified room temperature. All information in relation to the management of IMP will be detailed in the study specific Pharmacy Manual.

12.4 Accountability of the study treatment(s)

The study medication will be supplied to the Pharmacy by Uniphar Group, 4045 Kingswood Road, Citywest Business Park, Co Dublin. Standard shipment arrangements will continue. The site has the appropriate registration and authorization to manage schedule III drug according to local legal and regulatory requirements.

The KARMA-Dep 2 Pharmacy Manual will be issued to the study site and will include detailed instructions regarding: inventory, storage, randomisation, dispensing, dosing requirements, return processes and final disposition.

Upon delivery, receipt of the products will be inspected, recorded by PI/Designee and clinical trial labels applied. The products will then be transferred to the secure storage area as per local regulatory requirements and segregated from Pharmacy stock. The investigator is responsible for the control of the treatments under investigation. Adequate records for the receipt and disposition of the IMP will be maintained.

The PI/designee will complete and return all necessary paperwork as specified in the Pharmacy Manual. Unopened products, which are unused by end-of-trial, will be destroyed as per the study specific Pharmacy Manual and applicable hospital policies and Pharmacy SOPs.

Accountability and patient compliance with study treatments will be assessed by maintaining dispensing and return records.

Any quality issue (i.e. deficient clinical study material condition, appearance, documentation, labelling, etc.) at any stage during the study must be reported. Necessary corrective actions will be identified and implemented as determined by the Sponsor.

Should a discrepancy arise that cannot be accounted for, this should be reported to the Sponsor immediately and will be recorded and discussed by the TSC at their next meeting.

12.5 Assessment of compliance

In this study, interventions will be administered intravenously by the research team and thus there is limited opportunity for non-compliance with regards to actual treatment with the IMPs. The investigator is responsible for ensuring that the study treatment is administered in compliance with the protocol. Patient compliance will be assessed by maintaining dispensing records.

12.6 Overdose of study treatment

Given the safeguards in place (consultant Anaesthetist to prepare and administer infusions, assisted by members of the research team), it is deemed unlikely that an overdose of study treatment could occur. In the improbable event of an overdose, the study patient will be monitored for any change in neurological status or vital signs by the Consultant Anaesthetist, investigator and research team members. If there are signs of change, e.g. drowsiness, change in blood pressure/heart rate and a known overdose has occurred, the patient will be counselled and brought by ambulance, accompanied by a member of the research team, to the local Emergency Department (St. James's Hospital) for medical investigation. A letter detailing treatment and doses administered will be provided. Reasonable efforts will be made to contact the next of kin if the patient consents. Should the patient require medical investigation and/or treatment due to an overdose of study treatment, cost will be covered by the trial indemnity policy, unless due to negligence or malpractice. The Sponsor will be notified immediately following any overdose or suspected overdose.

12.7 Prior and concomitant therapy

Any medication, other than the study medication taken during the study, will be recorded in the CRF. Concomitant medications will be documented at the point of consent, before the first infusion (Visit 1), and changes will be noted at every assessment or intervention appointment thereafter.

12.7.1 Permitted medications/non- investigational medicinal products

All medications aside from those listed in section 12.7.2 below are permitted. Treatment-as-usual will continue for all participants during this study. However, where possible, patients taking any benzodiazepines should omit their dose on the morning of infusion sessions. As per the SmPc for Ketalar, diazepam is known to increase the half-life of ketamine and prolongs its pharmacodynamic effects. Concurrent use of diazepam or other benzodiazepines will increase plasma levels and reduce the clearance rate of ketamine. However, benzodiazepines do not appear to interact with the antidepressant effect of ketamine. No non-investigational medicinal products will be used outside authorisation for the purposes of this trial.

12.7.2 Prohibited medications

The following medications are contraindicated during the randomised treatment period as they may alter the pharmacokinetics of ketamine. Additionally, the medication theophylline is contraindicated as concomitant use of ketamine and theophylline may significantly reduce the seizure threshold with reports of unpredictable extensor-type seizures.

Contraindicated medications:

Ketoconazole
Voriconazole
Itraconazole
Fluconazole
Erythromycin
Telithromycin
Clarithromycin
Saquinavir
Nefazodone
Diltiazem
Verapamil
Theophylline

Participants taking any of these medications on randomisation will be excluded from the trial. Medication history will be checked at Baseline and each subsequent assessment, and participants who have been prescribed these medications during the trial will not receive further interventions (i.e. infusions of ketamine or midazolam). However, they will be followed up according to the framework presented here. Data collected will be included in intention-to-treat analyses if one infusion and one follow-up assessment have been completed. It is not permitted for patients to participate in investigational treatment studies while participating in this study.

13 SAFETY REPORTING

Safety and tolerability of the IMPs will be evaluated throughout the study, e.g. by AEs, laboratory values, vital signs and physical exam findings, etc.

13.1 Definitions

13.1.1 Adverse event (AE)

Any untoward medical occurrence in a patient or clinical trial patient administered a medicinal product and which does not necessarily have a causal relationship with this treatment.
An adverse event can therefore be any unfavourable and unintended sign (including an abnormal laboratory finding, for example), symptom or disease temporally associated with the use of a medicinal product, whether or not considered related to the medicinal product.

The adverse events reporting period will be from the time of consent to the end of the 24-week follow-up period. Prior medical history is not an AE unless there is a significant increase in frequency/severity.

13.1.2 Adverse reaction (AR)

All untoward and unintended responses to a medicinal product related to any dose.
The phrase 'responses to a medicinal product' means that a causal relationship between a study medication and an AE is at least a reasonable possibility, i.e., the relationship cannot be ruled out.

13.1.3 Serious adverse event

Any untoward medical occurrence or affect that at any dose:

- results in death,
- is life-threatening*,
- requires general medical hospitalisation or prolongation of existing hospitalisation,
- results in persistent or significant disability or incapacity,
- is a congenital anomaly or birth defect
- important medical events**

*Regarding a life-threatening event, this refers to an event in which the patient was at risk of death at the time of the event; it does not refer to an event that hypothetically might have caused death if it were more severe.
**Some medical events may jeopardise the patient or may require an intervention to prevent one of the above characteristics/consequences. Such events (hereinafter referred to as 'important medical events') should also be considered as 'serious' in accordance with the definition. The clinician should make a judgment call if they wish to report an event which does not fit any of the other serious criteria listed but which in their clinical judgement is an important medical event.

13.1.4 Severe adverse events

The term 'severity' is used here to describe the intensity of a specific event. This has to be distinguished from the term "serious".

13.1.5 Suspected unexpected serious adverse reactions (SUSAR)

An adverse reaction, the nature or severity of which is not consistent with the applicable product information (e.g. summary of product characteristics for an authorised medicinal product).

13.2 Evaluation of AEs and SAEs

13.2.1 Assessment of seriousness

The investigator should make an assessment of seriousness as defined in section 13.1.3 above.

13.2.2 Assessment of causality

All adverse events (AE) judged by either the investigator or the Sponsor as having a reasonable suspected causal relationship to an investigational medicinal product qualify as adverse reactions (AR).

The causality assessment given by the investigator cannot be downgraded by the Sponsor. The investigator/Sponsor must make an assessment of whether the AE/SAE is likely to be related to treatment according to the following definitions:

Definite: Where the event is considered to be related to the study medication.

Probable: The temporal relationship and absence of a more likely explanation suggest the event could be related to the study medication.

Possible: Although a relationship to the study medication cannot be completely ruled out, the nature of the event, the underlying disease, concomitant medication or temporal relationship make other explanations possible.

Unlikely related: A clinical event, including laboratory test abnormality, with a temporal relationship to drug administration which makes a causal relationship improbable, and in which other drugs, chemicals or underlying disease provide plausible explanations.

Not related: Where an event is not considered to be related to the study medication.

All AEs/SAEs judged as having a reasonable suspected causal relationship (e.g. possible, probable) to the study medication will be considered as ARs/SARs (serious adverse reaction; see 13.1.3 for definition of "serious").

All AEs/SAEs judged as being related (e.g. possible, probable) to an interaction between the study medication and another medication will also be considered to be ARs/SAR.

Alternative causes such as natural history of the underlying disease, concomitant therapy, other risk factors and the temporal relationship of the event to the treatment should be considered.

13.2.3 Assessment of severity

The investigator/sub-investigator will make an assessment of severity for each AE/SAE and record this on the CRF according to one of the following categories:

Mild: An event that is easily tolerated by the patient, causing minimal discomfort and not interfering with every day activities.

Moderate: An event that is sufficiently discomforting to interfere with normal everyday activities.

Severe: An event that prevents normal everyday activities.

Note: the term 'severe', should not be confused with 'serious', which is a regulatory definition based on patient/event outcome or action criteria.

13.2.4 Assessment of expectedness

The expectedness of an adverse reaction will be determined by the Chief Investigator delegated by Sponsor according to the relevant reference safety document, e.g. the summary of product characteristics for an authorised medicinal product, which is used according to the terms and conditions of the marketing authorisation. The Reference Safety Information (RSI) will be agreed with the HPRA at the time of clinical trial application and any updates to the RSI during the study will be notified to the PI who will determine if the RSI will need to be updated and therefore the HPRA notified.

13.2.5 Emergency unblinding procedures

Emergency unblinding can be performed by any investigator by opening one or all of the set of envelopes containing allocation information. These will be securely stored in the Ketamine Research Clinic where infusions are to be administered. Instructions for emergency unblinding will be included in the Ketamine Research Clinic and Assistant Director of Nursing's office.

13.3 Reporting procedures for all adverse events

All AEs occurring during the study observed by the investigator or reported by the patient, whether or not attributed to the study medication, will be recorded on the eCRF.

The following information will be recorded at a minimum: description, date of onset and end date, severity, assessment of relatedness to the study medication, other suspect medication or device, action taken and outcome. Follow-up information should be provided as necessary. For AEs that are "Serious" see section 13.4 below.

AEs considered related to the study medication as judged by an investigator will be followed until the event or sequelae have resolved or are considered stable. All related AEs that result in a patient's withdrawal from the study will be followed up until the final visit. It will be left to the investigator's clinical judgment whether or not an AE is of sufficient severity to require the patient's removal from treatment. A patient may also voluntarily withdraw from treatment due to what he or she perceives as an intolerable AE. If either of these occurs, the patient must undergo an end-of-study assessment and be given appropriate care under medical supervision until symptoms cease or the condition becomes stable.

Any pregnancy occurring during the clinical study and the outcome of the pregnancy should be recorded and followed-up for congenital abnormality or birth defect using the SAE reporting procedure outlined in section 13.6 below.

13.4 Reporting procedures for serious adverse events

13.4.1 Initial reporting of an SAE

The investigator or delegated site staff will report all serious adverse events immediately to the Sponsor-delegated Pharmacovigilance office (PVO) at SJH-CRF, except for those that the protocol identifies as not requiring immediate reporting.

The investigator will indicate, on the SAE form:

- Serious criterion (or criteria if more than one) – as per Section 13.1.3.
- Relationship of SAE to study drug/comparator – as per Section 13.2.2.

The study site will complete the SAE form and email it to: pharmacovigilance@tcd.ie. The minimum details needed to process an SAE are:

- An identifiable patient (Unique id number ONLY).
- An event that the Investigator has classified as serious.
- An investigational medication (study drug or comparator).
- An identifiable reporter (Name of person at study site who is reporting the event).

Receipt of the SAE report will be acknowledged by e-mail by the PV team within one working day. If incomplete, the initial SAE report should be followed by a detailed written report as soon as possible. The report will be made by the investigator to the PV office immediately upon becoming aware of the event. In no circumstances should this exceed **24 hours** following knowledge of the serious adverse event.

13.4.2 Follow up information:

Additional information received for a case (follow-up or corrections to the original case) should be detailed on a new SAE form and sent expeditiously to the PV office. The PV office will keep detailed records of all serious adverse events that are reported to them by the investigator or investigators.

13.4.3 Reporting Participant Data to SJH-CRF

The immediate and follow-up reports will identify patients only by the unique patient ID number assigned to them. Any accompanying source documentation (e.g. discharge summary, laboratory results etc.) should identify the patient by unique ID number only – patient identifiers i.e. date of birth, should be redacted before sending such documents.

13.4.4 Expectedness Assessment and Reporting by Sponsor

Expected/Unexpected: The Investigator will check any SARs for expectedness using the current reference safety information (RSI). If the SAR is unexpected this becomes a SUSAR. The Investigator and Pharmacovigilance Officer will notify the Sponsor of this classification and Pharmacovigilance staff will carry out unblinding (using the envelopes supplied to PV in SJH-CRF) and proceed to report the SUSAR to the relevant competent authorities (the HPRA in Ireland, who will onward report to the European Medicines Agency, via EudraVigilance) and the ethics committees concerned within the appropriate timelines as follows:

- Fatal or life-threatening SUSARs will be reported within **2 days** upon receipt of the SAE report.

- SUSARs which are not fatal or not life-threatening will be reported within **7 days** upon receipt of the SAE report.

The Sponsor or delegated PV office will inform all investigators concerned of relevant information about SUSARs that could adversely affect the safety of patients.

13.4.5 Annual Development Safety Update Report

In addition to the expedited reporting above, the PV office will assist the PI in the preparation of the annual Data Safety Update Report (DSUR). Following PI review and signoff PV will submit the DSUR to the competent authority (the HPRA in Ireland) and relevant ethics committee. DSUR format as per ICH guideline E2F - Note for guidance on development safety update reports.

13.5 Data Monitoring Committee (DMC)

The independent Data Monitoring Committee (DMC) will review blinded data on a six-monthly basis during the trial and will act according to the DMC Charter, which will be ratified at the organisational meeting.

The DMC will be an independent committee established prior to the commencement of the trial to assess progress, safety data and data security and will meet every six months during the trial. The DMC will hold an organisational meeting prior to recruitment. The Trial Management Group and trial statistician will prepare a report for the Committee circulated two weeks prior to each meeting. A charter will be ratified. No member of the DMC will have a conflict of interest with the Sponsor. Blinded data will be presented to the DMC for safety evaluation every six months. Listing of SAEs and AEs will be provided to the committee prior to the meeting and discussed. SUSARs and SARs will be discussed individually, as appropriate. Should the Committee wish to review unblinded data, this will be provided by an unblinded statistician who will otherwise not be analysing trial data. The DMC will report to the Trial Steering Committee (TSC), which has authority to decide whether the trial should be suspended or ended. Minutes of the DMC meetings including safety evaluations will be presented to the TSC at every meeting.

The advice of the DMC will be notified upon receipt by the Sponsor to the REC and CA that approved the protocol. With this notification a statement will be included indicating whether the advice will be followed. The composition of the DMC will include an independent statistician, an independent trial methodologist, and an independent clinical investigator.

PV will provide listings of SAEs/SARS/SUSARs for the DMC review at intervals agreed with the steering committee.

13.6 Trial Steering Committee

The TSC will comprise investigators, clinical experts not directly involved in the trial, a service user representative, and staff nominated by the Sponsor. The committee will also include members who are independent of the investigators, SPUH, funders and the Sponsor. The TSC will consider and act, as appropriate, upon the recommendations of the DMC and ultimately carries the responsibility for deciding on premature termination of the trial. The TSC will take

responsibility for the scientific validity of the study protocol, assessment of study quality and conduct as well as for the scientific quality of the final study report.

The TSC and DMC charters will have the relevant member details once established.

13.7 Pregnancy

Pregnancy is not considered an AE or SAE. However, the investigator will collect pregnancy information for female trial patients or female partners of male trial patients who become pregnant while participating in the study.

The investigator will record the information on a Pregnancy Notification Form and submit this to the Sponsor via the PV office (refer to 13.4.1). Any pregnancy that occurs in a trial patient or a trial patient's partner during a trial will be followed to outcome. It may be necessary to monitor the development of the new-born for an appropriate period post-delivery.

While ketamine has been shown to be teratogenic in rats, there are no data of its use during human pregnancy, particularly at sub-anaesthetic doses. Insufficient data are available on midazolam to assess its safety in pregnancy; however, other benzodiazepines have been associated with teratogenicity. There have been no studies of sub-anaesthetic doses of midazolam in pregnancy. Date of last menstrual period (LMP) will be recorded at recruitment and the first ketamine infusion along with the results of pregnancy testing. Information regarding the importance of adequate contraception during the trial and informing researchers if there is any possibility of pregnancy will be provided in the Patient Information Leaflet.

14 STATISTICS

14.1 Description of statistical methods

Analyses will be conducted in the intention-to-treat set comprising all participants who complete at least one infusion and one post-infusion evaluation. Data analyses will be performed blinded to allocation by CSTAR.

14.2 Determination of sample size

Based on a randomised trial of adjunctive serial ketamine infusions [1], 41 patients are required per initial randomisation group (n=82) to have 90% power to demonstrate, using a two-sided t-test at 5% level, that mean reduction in MADRS score in the ketamine group will be ≥8 points greater than that achieved in the midazolam group. This calculation conservatively assumes a standard deviation for the change in mean MADRS of 11, and that the assumptions of a t-test are broadly met, which is expected to be the case with approximately 40 patients per group. Allowing for a 20% dropout, we will recruit 52 patients per group (total n=104).

14.3 Demographic and baseline disease characteristics

Demographic and baseline disease characteristic data will be summarized for each treatment group by presenting frequency distributions and/or descriptive statistics.

14.4 Efficacy analysis

The primary analysis will be conducted once at end-of-trial by a statistician blinded to group labels. To produce this, a general linear model will be fitted to the MADRS scores, with trial arm, site and baseline MADRS as covariates, and using heteroscedasticity-consistent “sandwich” variance-covariance estimators [62]. The dependent variable will be the MADRS scores 24 hours after the final infusion, including where the full course of infusions was not completed.

The efficacy of the treatment will be evaluated by way of a statistical test of the coefficient for trial arm, the p-value for which will be compared to 0.05. The coefficient itself will be presented as the effect size, corresponding to an adjusted MADRS score mean difference between arms after the final infusion, and supplemented by a 95% confidence interval. For a single primary outcome, no adjustment to the type I error is needed.

No formal subgroup analyses are planned for the primary outcome, as low power to detect interactions with sub-grouping factors is expected. Binary secondary outcomes of response, remission, and relapse will be summarised per treatment arm and descriptively presented.

Similar generalised linear models to the primary outcome analysis will be used to describe continuous secondary outcomes (QIDS-SR_{16} and MoCA). As these secondary outcomes were not powered by design, we will avoid claims of statistical significance and focus on interpretation of effect sizes and confidence intervals. P-values will be presented to complement this and an adjustment for the False Discovery Rate using the Benjamini-Hochberg approach will be carried out [63].

Non-continuous secondary outcomes relating to safety and tolerability will be presented descriptively; no formal analyses are planned due to anticipated low power.

A complete technically detailed Statistical Analysis Plan has been formulated and approved by the DMC and TSC.

14.5 Safety analysis

Descriptive statistics of adverse events and serious adverse events, and other incidents will be reported, per arm, as per-patient averages in the final trial report.

14.6 Criteria for the termination of the trial

Please see section 11.4 “Definition of end-of-trial”.

14.7 Procedure for accounting for missing, unused and spurious data

The primary analysis will adopt an analytic model using pairwise deletion of the dependent variable (complete case analysis). It is not expected that covariates (trial arm, site and baseline MADRS score) will have any missing values. In the event that more than 5% of participants are missing the endpoint, multiple imputation by chained equations will be performed using baseline and subsequent MADRS scores to attempt to recover as much information as possible.

14.8 Procedure for reporting any deviation(s) from the original statistical plan

Deviations from the original statistical plan will be reported to the Sponsor within a timely interval and discussed by the Trial Steering Committee at the next meeting. These will be recorded and justified in the final report. Where a deviation from the original statistical plan is judged by the investigators or Sponsor to comprise a substantial amendment to the trial protocol, the standard procedure for reporting substantial amendments to the HPRA will be followed. Such deviations will also be updated to the trial registry entry, and their occurrence and impact on the interpretation of the result will be explored in the final trial report and dissemination.

15 DIRECT ACCESS TO SOURCE DATA/DOCUMENTS

The PI and site will ensure to grant direct access will be granted to authorised representatives from the Sponsor, REC, host institution and the regulatory authorities to permit trial-related monitoring, audits and inspections.

16 DATA HANDLING AND RECORD KEEPING

Data will be entered, handled and stored at St Patrick’s University Hospital. It will be anonymised and then processed by members of the research team within the Dept of Psychiatry and Trinity College Institute of Neuroscience in Trinity College Dublin, Clinical Research Facility at University of Galway, Queens University Belfast, and at the Centre for Support and Training, University College Dublin. GDPR data protection polices will be followed.

16.1 Data collection, source documents and case report forms (CRF)

Source documents for this study must be attributable, legible, contemporaneous, original, accurate and complete. They include clinical notes, medication records, and study-specific data collection documents. Information will be extracted from these documents and recorded accurately on secure eCRFs. If an error is made in the source, the error will be crossed through with a single line in such a way that the original entry can still be read, initialled and dated. The correct entry will then be clearly inserted, and the alterations will be initialled and dated by the investigator. Data reported on the CRF that are derived from source documents must be consistent with the source documents or the discrepancies will be explained. The PI must review, approve and sign the eCRF to attest that the contents are accurate.

All documents will be stored safely in confidential conditions in a designated locked filing cabinet in a locked office within the Research Building at St Patrick's University Hospital and confidentiality will be observed at all times.

With the exception of the informed consent form and information exported directly from the electronic health records, patients will be referred to only by their patient identification number on all study-specific documents, whether hard copies or electronic.

Data analysis will also take place in other facilities (i.e. CSTAR, Queens University Belfast) and will be anonymised prior to secure transfer to CSTAR for analysis.

16.2 Data reporting

Central data management will be performed by the Data Management Centre at the Health Research Board Clinical Research Facility at University of Galway (CRFG). Local user access to the electronic CRF will be controlled via assigned usernames and passwords, approved by the study Data Manager based at CRFG. Access to the central study database will be governed by CRFG Standard Operating Procedures and signed off by the Lead Site Investigator. Audit trails will log all transactions of data into and out of the system including time, date, user ID and the records involved. All external electronic communication with the central database will be protected by using Secure Socket Layer technology. The main database will be hosted in a secure enterprise scale data centre.

Once registered to the trial, the patient will be provided with a unique, study-specific participant identifier number and this will be the only way the patient will be identified in the database. Data will be directly entered into the Clinical Data Management System (CDMS) by the site staff. Data entry is by double data entry. Data queries will be generated within the CDMS for the investigational site as required to clarify data discrepancies or request missing information. The designated site staff will be required to respond to these queries and these responses with be reviewed by the Data Management Team. Any amendments to the data will be tracked within the audit trail of the CDMS.

The Data Manager will develop a Data Management Plan (DMP) which will detail all activities relating to the management of the clinical data. All project specific data management documentation will be filed in a Data Management File (DMF). The Data management team will also develop a CDMS to store the clinical data. This will be developed following the relevant Data Management SOPs and adhering to ISO guidelines.

Reconciliation between the safety and clinical databases will be carried out every quarter as per the study reconciliation plan. The PVO in SJH-CRF will provide the Data Management Centre (University of Galway) with a line listing of the SAEs in their safety database at quarterly intervals. These will be compared with the clinical database. Line listings for the Clinical and Safety database will then be reconciled and any queries sent to the Investigator and sponsor.

17 RETENTION OF ESSENTIAL DOCUMENTS

Data derived from essential trial documents will be retained for a period of 25 years in accordance with Clinical Trial Regulation (CTR) (EU Regulation 536/2014). This information is included in the Participant Information Leaflet and the Informed Consent Form.

The essential documents are defined as those that individually and collectively permit evaluation of the conduct of the trial and the quality of the data produced. These documents will be filed in an organised way that facilitates management of the clinical trial, audit and inspection by competent authorities and will be readily available on request.

As this is an academic study, recommendations regarding retention of essential documents for EMA approval/ clinical development of the IMP are not applicable to this study.

The investigator/institution should agree to retain the trial-related essential documents as required by the applicable regulatory requirements and until the Sponsor provides written instructions informing the investigator/institution these documents are no longer necessary.

18 QUALITY CONTROL AND QUALITY ASSURANCE PROCEDURES

This study will be conducted in accordance with the current approved protocol, ICH GCP, relevant regulations and standard operating procedures. The measures taken to ensure data obtained is accurate, complete and reliable include:

(i) Researchers will attend Good Clinical Practice training

(ii) Researchers will be trained in administration of the primary assessment tool used in this study, the MADRS. Administration will be according to specified guidelines and training will be repeated every 6 months to ensure inter-rater reliability.

(iii) A trial-specific Data Entry and Quality Assurance Protocol will be followed by all researchers.

19 MONITORING

Data for each patient will be recorded on an electronic case report form (eCRF). Data collection must be completed for each patient who signs an informed consent form (ICF) and is administered a study drug.

In accordance with GCP and ICH guidelines, the study monitor will carry out source document verification at regular intervals to ensure that the data collected in the eCRF are accurate and reliable.

The investigator must permit the monitor, the REC, the Sponsor's internal auditors, and representatives from regulatory authorities direct access to all study-related documents and pertinent hospital or medical records for confirmation of data contained within the eCRFs.

20 AUDITS AND INSPECTIONS

This trial may be subject to internal or external auditing or inspections procedure to ensure adherence to GCP. Access to all trial-related documents will be given at that time.

In accordance with the legislation, the trial master file comprising the essential documents which enable both the conduct of the trial and the quality of the data produced to

be evaluated will be available to provide the basis for the GCP inspection. Responses to a GCP inspection report will be provided within 30 working days of the date of issue.

21 ETHICS

21.1 Declaration of Helsinki

The Sponsor will ensure that this study is conducted in accordance with the ethical principles that have their origins in the Declaration of Helsinki.

21.2 Good Clinical Practice

This study will be conducted in accordance with Good Clinical Practice (GCP), as defined by the International Conference on Harmonisation (ICH) and in accordance with the ethical principles underlying European Union Directive 2001/20/EC and 2005/28/EC.

21.3 Approvals

Required documents including the protocol, informed consent form, patient information leaflet, investigational medicinal product dossier, investigators brochure and any other required documents will be submitted to a recognised research ethics committee and the competent authority for written approval.

The Sponsor will submit and obtain approval from the above parties for substantial amendments to the original approved documents.

21.4 Informed consent

Prior to any study-related screening procedures being performed on the patient, the informed consent statement will be reviewed, signed and dated by the patient and investigator. Informed consent will be obtained verbally at each intervention (e.g. infusion sessions).

Potential participants will be provided with a Patient Information Leaflet and verbal information at the first point of contact with a member of the research team. Verbal assent will be sought at each treatment step and documented in the source notes. Time will be provided to address questions. Every effort will be made to provide adequate time for the participant to consult with family, friends and their general practitioner prior to making a decision. However, as it is common for treatment for depression to begin on the same day as admission to hospital, in some cases provision of information and the process of obtaining consent may take place on the same day. Participants will be encouraged to reflect on the information provided and ask questions, but it is recognised that some participants may prefer to make a decision at the first point of contact and this will also be accommodated.

21.5 Benefits and risks assessment

This study may further the understanding of depression, a major public health issue, and provide further information about a potential adjunctive therapy to routine care to help accelerate recovery in patients with moderate or severe depression admitted to hospital. In

designing this study, all efforts have been made to reduce the risk to and burden for participants. It is believed that risk to and burden for the patient will be in proportion to the potential value of this research. There are no guaranteed direct benefits to participants. However, all participants may benefit indirectly from participation in terms of increased awareness of mental health issues. Some participants may benefit from the administration of ketamine in terms of accelerating their recovery, but this is not guaranteed. This study will not include incapacitated adults or minors. Issues regarding specific vulnerable populations are addressed individually below.

(i) This study requires inclusion of **adults with mental illness** to address the research question. Only those who have capacity to provide valid informed consent will be invited to participate. Where there is any concern expressed about the capacity of a person to make his/her own treatment decisions, a capacity assessment is performed by the treating team. This assessment will guide investigators in selecting those who have capacity to consent to enrolment in the trial. The trial is expected to benefit participants who have a mental illness indirectly by improving scientific knowledge of a major mental health issue. There are possible direct and indirect benefits to participants with a mental illness in terms of reduced relapse rate and increased awareness of mental health through participation, however there is no guaranteed direct benefit.

(ii) **Women of childbearing** age are defined by the Irish Central Statistics Office as women of ages 15-49 (Census 2011 This is Ireland (Part 1) - CSO - Central Statistics Office). Women of this age group will not be excluded from this study as this group constitutes a significant proportion of the population of interest, i.e. people with MDD. Irish women are more likely to suffer from depression than men and 25% of women in Ireland will require treatment for depression in their lifetime. Thus the primary study objective cannot be accurately achieved without inclusion of women of childbearing age. Previous studies of ketamine have included women of childbearing age and precautions will be taken as detailed here to ensure adequate contraception is in place throughout the trial. Birth control methods which may be considered as highly effective are methods that can achieve a failure rate of less than 1% per year when used consistently and correctly. Such methods include combined (estrogen and progestogen containing) hormonal contraception associated with inhibition of ovulation, progestogen-only hormonal contraception associated with inhibition of ovulation, intrauterine device (IUD), intrauterine hormone-releasing system (IUS), bilateral tubal occlusion, vasectomised partner and sexual abstinence.

21.6 Patient confidentiality

Monitors, auditors, and other authorized agents of the Sponsor and/or its designee, the REC approving this research, as well as that of any other applicable agency(ies), will be granted direct access to the study patients' original medical records for verification of clinical study procedures and/or data, without violating the confidentiality of the patients to the extent permitted by the law and regulations. In any presentations of the results of this study or in publications, the patients' identity will remain confidential.

The trial staff will ensure that the patients' anonymity is maintained. The patients will be identified only by a patient's identification number on the CRF and any database. All documents will be stored securely. The study will comply with the Data Protection Act.

21.7 Other ethical considerations

Use of placebo/active comparator: Participants in this pilot trial will continue usual care as recommended by their responsible clinical team. They may receive an additional treatment as part of this trial but will not be denied any treatment for the purposes of this trial. Participants will be provided with verbal and written information regarding the 1:1 randomisation strategy and the possibility of being randomised to an active comparator group. There is no evidence to suggest that patients who are randomised to the active comparator arm of the study will suffer poorer outcomes, as a definitive study of this nature has not yet been performed. The DMC will monitor data for safety parameters throughout the trial including the possibility of a large discrepancy between midazolam and ketamine groups, and in the event of such a circumstance, will follow the specifications in this protocol.

22 FINANCING AND INSURANCE/INDEMNITY

This trial has been funded by an award from the Health Research Board: DIFA-2018-013. Insurance will be provided by indemnity cover for research in place in both Trinity College Dublin (Sponsor) and St Patrick's Mental Health Services. This will be in place once the trial is approved by the St Patrick's Mental Health Services REC, an application for which will be submitted following approval by the authorised clinical trials REC of Mater Misericordiae Hospitals Group. Physicians involved in the trial will have medical malpractice insurance.

23 CLINICAL STUDY REPORT AND PUBLICATION POLICY

The publication policy involves formal presentation of preliminary study findings at national and international neuroscience and psychiatry meetings. Final findings will be submitted for peer-review and publication in relevant scientific journals and upon publication they may be further publicized in national and international print and electronic media through the Trinity and St. Patrick's University Hospital websites and public relations departments. Further knowledge dissemination will include registering the trial in the EudraCT database and publication of the trial protocol in a peer-reviewed journal.

Research progress and developments will be regularly presented at medical "grand rounds" in St. Patrick's University Hospital, and in-house research meetings and seminars at the Department of Psychiatry, Trinity College Dublin and Trinity College Institute of Neuroscience. Information about the research programme and other ongoing related depression research will also be contributed through our group's website (https://www.tcd.ie/medicine/psychiatry/research/depression/) available to the general public.

Published data must not compromise the objectives of the study. All patient facing materials will be submitted and approved by the REC before they are given to patients.

The clinical study report will be presented to the REC and HPRA within one year of the completion or cancellation of the trial. The format of this summary will comply with the EU Note for Structure and Content of Clinical Study Reports (CPMP/ICH/137/95). The clinical study report will be signed by the principal investigator.

24 REFERENCES

1. Singh, J.B., et al., *A Double-Blind, Randomized, Placebo-Controlled, Dose-Frequency Study of Intravenous Ketamine in Patients With Treatment-Resistant Depression.* American Journal of Psychiatry, 2016. **173**(8): p. 816-826.
2. Wittchen, H.U., et al., *The size and burden of mental disorders and other disorders of the brain in Europe 2010.* European Neuropsychopharmacology, 2011. **21**(9): p. 655-679.
3. Rush, A.J., et al., *Acute and longer-term outcomes in depressed outpatients requiring one or several treatment steps: A STAR*D report.* American Journal Of Psychiatry, 2006. **163**(11): p. 1905-1917.
4. Zanos, P., et al., *Convergent Mechanisms Underlying Rapid Antidepressant Action.* Cns Drugs, 2018. **32**(3): p. 197-227.
5. Daly, E.J., et al., *Efficacy of Esketamine Nasal Spray Plus Oral Antidepressant Treatment for Relapse Prevention in Patients With Treatment-Resistant Depression: A Randomized Clinical Trial.* JAMA Psychiatry, 2019. **76**(9): p. 893-903.
6. Kishimoto, T., et al., *Single-dose infusion ketamine and non-ketamine N-methyl-D-aspartate receptor antagonists for unipolar and bipolar depression: a meta-analysis of efficacy, safety and time trajectories.* Psychological Medicine, 2016. **46**(7): p. 1459-1472.
7. Xu, Y., et al., *Effects of Low-Dose and Very Low-Dose Ketamine among Patients with Major Depression: a Systematic Review and Meta-Analysis.* International Journal of Neuropsychopharmacology, 2016. **19**(4).
8. Han, Y., et al., *Efficacy of ketamine in the rapid treatment of major depressive disorder: a meta-analysis of randomized, double-blind, placebo-controlled studies.* Neuropsychiatric Disease and Treatment, 2016. **12**: p. 2859-2867.
9. Wilkinson, S.T., et al., *The Effect of a Single Dose of Intravenous Ketamine on Suicidal Ideation: A Systematic Review and Individual Participant Data Meta-Analysis.* American Journal of Psychiatry, 2018. **175**(2): p. 150-158.
10. Sanacora, G., et al., *A Consensus Statement on the Use of Ketamine in the Treatment of Mood Disorders.* Jama Psychiatry, 2017. **74**(4): p. 399-405.
11. Short, B., et al., *Side-effects associated with ketamine use in depression: a systematic review.* Lancet Psychiatry, 2018. **5**(1): p. 65-78.

12. Murrough, J.W., et al., *Rapid and Longer-Term Antidepressant Effects of Repeated Ketamine Infusions in Treatment-Resistant Major Depression.* Biological Psychiatry, 2013. **74**(4): p. 250-256.
13. Shiroma, P.R., et al., *Augmentation of response and remission to serial intravenous subanesthetic ketamine in treatment resistant depression.* Journal of Affective Disorders, 2014. **155**: p. 123-129.
14. Ionescu, D.F., et al., *Repeat-dose ketamine augmentation for treatment-resistant depression with chronic suicidal ideation: A randomized, double blind, placebo controlled trial.* J Affect Disord, 2019. **243**: p. 516-524.
15. Daly, E.J., et al., *Efficacy and Safety of Intranasal Esketamine Adjunctive to Oral Antidepressant Therapy in Treatment-Resistant Depression A Randomized Clinical Trial.* Jama Psychiatry, 2018. **75**(2): p. 139-148.
16. Fedgchin, M., et al., *Efficacy and Safety of Fixed-Dose Esketamine Nasal Spray Combined With a New Oral Antidepressant in Treatment-Resistant Depression: Results of a Randomized, Double-Blind, Active-Controlled Study (TRANSFORM-1).* Int J Neuropsychopharmacol, 2019.
17. Wilkinson, S.T., et al., *Impact of midazolam vs. saline on effect size estimates in controlled trials of ketamine as a rapid-acting antidepressant.* Neuropsychopharmacology, 2019. **44**(7): p. 1233-1238.
18. Caddy, C., et al., *Ketamine and other glutamate receptor modulators for depression in adults.* Cochrane Database of Systematic Reviews, 2015(9).
19. McCloud, T.L., et al., *Ketamine and other glutamate receptor modulators for depression in bipolar disorder in adults.* Cochrane Database of Systematic Reviews, 2015(9).
20. Arain, M., et al., *What is a pilot or feasibility study? A review of current practice and editorial policy.* Bmc Medical Research Methodology, 2010. **10**.
21. Pennybaker, S.J., et al., *Symptomatology and predictors of antidepressant efficacy in extended responders to a single ketamine infusion.* J Affect Disord, 2017. **208**: p. 560-566.
22. Niciu, M.J., et al., *Features of dissociation differentially predict antidepressant response to ketamine in treatment-resistant depression.* J Affect Disord, 2018. **232**: p. 310-315.
23. Bremner, J.D., et al., *Measurement of dissociative states with the Clinician-Administered Dissociative States Scale (CADSS).* Journal of Traumatic Stress, 1998. **11**(1): p. 125-136.
24. Finnegan, M., et al., *Ketamine for depression relapse prevention following electroconvulsive therapy: protocol for a randomised pilot trial (the KEEP-WELL trial).* Pilot and Feasibility Studies, 2016. **2**(38): p. 1-11; DOI 10.1186/s40814-016-0080-0.
25. Finnegan, M., et al., *Ketamine Versus Midazolam for Depression Relapse Prevention Following Successful Electroconvulsive Therapy: A Randomized Controlled Pilot Trial.* J ECT, 2019. **35**(2): p. 115-121.
26. Wilkinson, S.T., et al., *Impact of midazolam vs. saline on effect size estimates in controlled trials of ketamine as a rapid-acting antidepressant* Under review, 2019.

27. Fava, M., et al., *Double-blind, placebo-controlled, dose-ranging trial of intravenous ketamine as adjunctive therapy in treatment-resistant depression (TRD).* Mol Psychiatry, 2018.
28. Murrough, J.W., et al., *Antidepressant efficacy of ketamine in treatment-resistant major depression: a two-site randomized controlled trial.* Am J Psychiatry, 2013. **170**(10): p. 1134-42.
29. Murrough, J.W., et al., *Rapid and longer-term antidepressant effects of repeated ketamine infusions in treatment-resistant major depression.* Biol Psychiatry, 2013. **74**(4): p. 250-6.
30. Wan, L.B., et al., *Ketamine safety and tolerability in clinical trials for treatment-resistant depression.* J Clin Psychiatry, 2015. **76**(3): p. 247-52.
31. Montgomery, S.A. and M. Asberg, *NEW DEPRESSION SCALE DESIGNED TO BE SENSITIVE TO CHANGE.* British Journal of Psychiatry, 1979. **134**(APR): p. 382-389.
32. Mathew, S.J., et al., *Riluzole for relapse prevention following intravenous ketamine in treatment-resistant depression: a pilot randomized, placebo-controlled continuation trial.* Int J Neuropsychopharmacol, 2010. **13**(1): p. 71-82.
33. Price, R.B., et al., *Effects of intravenous ketamine on explicit and implicit measures of suicidality in treatment-resistant depression.* Biol Psychiatry, 2009. **66**(5): p. 522-6.
34. aan het Rot, M., et al., *Safety and efficacy of repeated-dose intravenous ketamine for treatment-resistant depression.* Biol Psychiatry, 2010. **67**(2): p. 139-45.
35. Lenderking, W.R., et al., *Daily process methodology for measuring earlier antidepressant response.* Contemporary Clinical Trials, 2008. **29**(6): p. 867-877.
36. Williams, J.B. and K.A. Kobak, *Development and reliability of a structured interview guide for the Montgomery Asberg Depression Rating Scale (SIGMA).* Br J Psychiatry, 2008. **192**(1): p. 52-8.
37. Zwarenstein, M., et al., *Improving the reporting of pragmatic trials: an extension of the CONSORT statement.* British Medical Journal, 2008. **337**.
38. Schulz, K.F., D.G. Altman, and D. Moher, *CONSORT 2010 statement: updated guidelines for reporting parallel group randomised trials.* Bmj, 2010. **340**: p. c332.
39. Semkovska, M., et al., *Bitemporal versus high-dose unilateral twice-weekly electroconvulsive therapy for depression (EFFECT-Dep): a pragmatic, randomized, non-inferiority trial.* American Journal of Psychiatry, 2016. **173**(4): p. 408-417.
40. Sheehan, D.V., et al., *The Mini-International Neuropsychiatric Interview (MINI): The development and validation of a structured diagnostic psychiatric interview for DSM-IV and ICD-10.* Journal of Clinical Psychiatry, 1998. **59**: p. 22-33.
41. Fava, M., *Diagnosis and definition of treatment-resistant depression.* Biol Psychiatry, 2003. **53**(8): p. 649-59.
42. Rush, A.J., et al., *The 16-Item Quick Inventory of Depressive Symptomatology (QIDS), clinician rating (QIDS-C), and self-report (QIDS-SR): a psychometric evaluation in patients with chronic major depression.* Biol Psychiatry, 2003. **54**(5): p. 573-83.

43. Nasreddine, Z.S., et al., *The Montreal Cognitive Assessment, MoCA: a brief screening tool for mild cognitive impairment.* J Am Geriatr Soc, 2005. **53**(4): p. 695-9.
44. Chernik, D.A., et al., *Validity and reliability of the Observer's Assessment of Alertness/Sedation Scale: study with intravenous midazolam.* J Clin Psychopharmacol, 1990. **10**(4): p. 244-51.
45. Overall, J.E. and D.R. Gorham, *The Brief Psychiatric Rating Scale*. Psychological Reports, 1962. **10**(3): p. 799-812.
46. Young, R.C., et al., *A rating scale for mania: reliability, validity and sensitivity.* Br J Psychiatry, 1978. **133**: p. 429-35.
47. Wisniewski, S.R., et al., *Self-rated global measure of the frequency, intensity, and burden of side effects.* J Psychiatr Pract, 2006. **12**(2): p. 71-9.
48. Rush, A.J., et al., *The 16-item Quick Inventory of Depressive Symptomatology (QIDS), clinician rating (QIDS-C), and self-report (QIDS-SR): A psychometric evaluation in patients with chronic major depression.* Biological Psychiatry, 2003. **54**(5): p. 573-583.
49. Overall, J.E. and D.R. Gorham, *The brief psychiatric rating scale*. Psychological Reports, 1962. **10**: p. 799-812.
50. Young, R.C., et al., *RATING-SCALE FOR MANIA - RELIABILITY, VALIDITY AND SENSITIVITY*. British Journal of Psychiatry, 1978. **133**(NOV): p. 429-435.
51. Perry, E.B., et al., *Psychiatric safety of ketamine in psychopharmacology research.* Psychopharmacology, 2007. **192**(2): p. 253-260.
52. Julayanont, P., et al., *Montreal Cognitive Assessment Memory Index Score (MoCA-MIS) as a Predictor of Conversion from Mild Cognitive Impairment to Alzheimer's Disease*. Journal of the American Geriatrics Society, 2014. **62**(4): p. 679-684.
53. Wan, L.B., et al., *Ketamine Safety and Tolerability in Clinical Trials for Treatment-Resistant Depression*. Journal of Clinical Psychiatry, 2015. **76**(3): p. 247-252.
54. Popova, V., et al., *Efficacy and Safety of Flexibly Dosed Esketamine Nasal Spray Combined With a Newly Initiated Oral Antidepressant in Treatment-Resistant Depression: A Randomized Double-Blind Active-Controlled Study.* Am J Psychiatry, 2019. **176**(6): p. 428-438.
55. Zaki, N., et al., *Long-term safety and maintenance of response with esketamine nasal spray in participants with treatment-resistant depression: interim results of the SUSTAIN-3 study.* Neuropsychopharmacology, 2023.
56. Dean, R.L., et al., *Ketamine and other glutamate receptor modulators for depression in adults with unipolar major depressive disorder.* Cochrane Database Syst Rev, 2021. **9**(9): p. CD011612.
57. Williams, N.R., et al., *Attenuation of Antidepressant Effects of Ketamine by Opioid Receptor Antagonism*. American Journal of Psychiatry, 2018: p. appiajp201818020138.
58. Schatzberg, A.F., *A Word to the Wise About Intranasal Esketamine*. Am J Psychiatry, 2019. **176**(6): p. 422-424.
59. Rickels, K., et al., *Physician Withdrawal Checklist (PWC-20).* J Clin Psychopharmacol, 2008. **28**(4): p. 447-51.

60. Peveler, R., et al., *A randomised controlled trial to compare the cost-effectiveness of tricyclic antidepressants, selective serotonin reuptake inhibitors and lofepramine.* Health Technology Assessment, 2005. **9**(16): p. 1-134,iii.
61. Herdman, M., et al., *Development and preliminary testing of the new five-level version of EQ-5D (EQ-5D-5L).* Quality of Life Research, 2011. **20**(10): p. 1727-1736.
62. Long, J.S. and L.H. Ervin, *Using Heteroscedasticity Consistent Standard Errors in the Linear Regression Model.* The American Statistician, 2000. **54**(3): p. 217-224.
63. Benjamini, Y. and Y. Hochberg, *Controlling the False Discovery Rate: A Practical and Powerful Approach to Multiple Testing.* Journal of the Royal Statistical Society: Series B (Methodological), 1995. **57**(1): p. 289-300.

KARMA-Dep (2)

Statistical Analysis Plan

Version history:

Version No.	Date	Contributor	Notes
0.1	12 May 2022	Ricardo Segurado	
0.2	13 May 2022	Ricardo Segurado	
0.3	6 July 2022	Ricardo Segurado	
0.4	9 September 2022	Ricardo Segurado	
0.9	26 November 2022	Ricardo Segurado	
1.0	26 November 2022	Ricardo Segurado	
2.0	9 May 2023	Ricardo Segurado	Approved by DMC
2.1	03 July 2024	Cathal Walsh	Approved by DMC

Table of Contents

1. Background and Introduction 2
1.1 Study Objectives 3
1.2.Study design........ 4
2. Data management 4
2.1 Missing data........ 4
2.2 Analysis sets........ 5
3. Statistical methods 5
3.1 Descriptive statistics 5
3.2 Analysis of efficacy........ 5
3.3 Further analyses........ 7
3.3.1 Sensitivity analysis of primary endpoint 7
3.2.2 Analysis of safety 8
3.2.3 Analysis of secondary endpoints........ 8
4. References 8

1. Background and Introduction

The background to the trial is described in the associated protocol paper (Jelovac et al., 2023). Depression is a leading cause of disability worldwide with a lifetime prevalence of 20% (Hasin et al., 2018). Despite intensive research efforts over many decades, pharmacotherapy for depression has remained focused on drugs targeting monoamine neurotransmitters. However, only about 30% of patients achieve remission after a standard first-line monoaminergic treatment (e.g. an SSRI) and about 30% of patients do not respond after multiple trials of various antidepressant medications (Rush et al., 2006). In addition to the problem of treatment-resistance, these standard treatments can take weeks to exert their therapeutic effects. There is thus a need for novel and faster-acting treatments for depression. The dissociative anaesthetic ketamine, which targets the excitatory neurotransmitter glutamate, may be one such approach.

When given as a single slow infusion at a sub-anaesthetic dose (0.5mg/kg of body weight), ketamine has been shown to have rapid-onset antidepressant effects on core symptoms of depression, including suicidal ideation (Han et al., 2016; Kishimoto et al., 2016; Price et al., 2022; Wilkinson et al.,

2018; Xu et al., 2016). Recent Cochrane reviews of ketamine in unipolar (Dean, Hurducas, et al., 2021) and bipolar depression (Dean, Marquardt, et al., 2021) found it appeared to be more efficacious than placebo at 24 hours after infusion. However, these effects typically last less than a week. A definitive role for ketamine in managing depression is not yet agreed upon (McIntyre et al., 2021; Sanacora et al., 2017). It may be that repeated infusions of ketamine have a more sustained antidepressant effect. There is an unmet clinical need for larger and longer-term parallel group trials to assess safety and efficacy of repeated ketamine infusions. The ongoing KARMA-Dep (2) Trial attempts to address these unanswered questions.

This document aims to set out in necessary detail the procedures for the statistical treatment of the data arising from the KARMA-Dep (2) trial. It therefore contains a brief outline of relevant features of this trial, the procedures for the manipulation of data and construction of new variables for the purposes of analysis, and instructions for the analysis of the primary endpoints leading to a conclusion about efficacy. An outline of further and secondary analyses is also provided. This document also outlines which parameter estimates and their precision will be reported.

It is expected that this document will undergo revisions as variable names, data structure or other aspects of the trial may be amended, and as best practice for the statistical analysis of this trial design is revised by the statistical community. Any revisions will be completed before the final dataset is provided to the trial statistician and statistical analysis of the trial commences. The Trial Steering Committee and the Data Monitoring Committee will sign-off on the final version of this document.

1.1 Study Objectives

The objective of this randomised, controlled rater- and patient-blinded trial is to test the efficacy of repeated ketamine infusions for the improvement of depressive symptoms in a population of inpatients with a DSM-5 diagnosis of a major depressive episode (MDE) in the context of major depressive disorder (MDD) or bipolar affective disorder (BPAD). The Montgomery-Åsberg Depression Rating Scale (MADRS; Montgomery & Åsberg, 1979) score is the primary outcome measure of depressive symptoms.

The monitoring of the safety profile of ketamine infusions is also an objective.

1.2. Study design

This trial will adopt a two-arm parallel longitudinal design with concealed allocation and blinded (to all trial participants, treating clinicians, and triallists except the pharmacist and administering anaesthetist) infusions of the investigational medicinal product or active control substance. Recruitment was initially planned to be from two sites: St Patrick's University Hospital, a 241-bed inpatient psychiatric facility located in central Dublin and its sister facility, the 52-bed St Patrick's Hospital Lucan, located in suburban/semirural County Dublin, Ireland. During the COVID-19 pandemic, the second site served as an isolation facility. As such, the second trial site was never activated.

Based on a randomised trial of adjunctive serial ketamine infusions (Singh et al., 2016), 41 patients are required per initial randomisation group (n=82) to have 90% power to demonstrate, using a two-sided t-test at 5% level, that mean change in MADRS score in the ketamine group will be ≥8 points that achieved in the midazolam group. This calculation conservatively assumes a standard deviation for the change in mean MADRS of 11, and that the assumptions of a t-test are broadly met, which is expected to be the case with approximately 40 patients per group. Anticipating a 20% withdrawal rate, the recruitment target is 52 patients per group (total n=104).

The outcomes of the trial will be assessed according to the schedule in the Protocol. Here we focus on the schedule for the primary outcome, the MADRS. This will be assessed at Visit 0 (screening) at which point it forms part of the exclusion criteria, with patients with scores less than 20 being excluded from the trial. Visits 1 through 8 will take place twice a week as scheduling allows (averaging 3-4 days separation), commencing as soon as consent is obtained and all eligibility criteria are verified to have been met. At each of these 8 visits infusion of the treatment will occur. The MADRS will be measured 40 minutes prior to the infusion commencing, 60 minutes after the infusion commences, 120 minutes after the infusion commences, and 24 hours after the infusion is completed.

2. Data management

2.1 Missing data

Missing data will be tabulated for each outcome variable at each time-point and explored visually. Missing data rates per patient and per visit will be calculated. The proportion of participants with at least one missing endpoint measurement will be obtained to contrast the loss of information between listwise and pairwise approaches.

The primary analysis will adopt an analytic model using pairwise deletion of the dependent variable (complete case analysis). It is not expected that covariates (trial arm and baseline (-40 mins at visit 1) MADRS score) will have any missing values. In the event that more than 5% of participants are missing the endpoint, multiple imputation by chained equations will be performed using baseline and subsequent MADRS scores to attempt to recover as much information as possible.

The primary outcome MADRS and other outcomes are composite scales. There are not expected to be any missing individual items present in the data set as received, as only the summary score will be entered to the eCRF.

2.2 Analysis sets

The primary analysis set will comprise all patients in the group to which they are randomised, irrespective of missing data or protocol violations. Every effort will be made during the trial conduct to collect outcome data for patients who withdraw (for whatever reason) from the treatment or from the study. Participant observations will be ascribed to the arm to which that participant was randomised, whether or not the randomised treatment was received.

The analysis set for safety analyses will comprise all participants who received at least one infusion.

3. Statistical methods

3.1 Descriptive statistics

Descriptive statistics will be constructed for all measures of trial throughflow, including patient throughflow, treatment compliance, and assessment completion. The absolute frequencies will be used to construct a flow chart according to the CONSORT template.

Demographic and clinical characteristics at baseline will be reported using appropriate descriptive statistics, as will the baseline values of the primary and other outcome measures.

3.2 Analysis of efficacy

We present an estimand-to-analysis table (Kang et al., 2022) below (Table 1) to link the objectives of the trial to the planned statistical analysis, in line with the International Conference on Harmonisation E9 (R1) addendum on estimands and sensitivity analysis in clinical trials (European Medicines Agency, 2020).

Table 1 Estimand-to-analysis table for KARMA-Dep (2)

**Objective:** To test the efficacy of ketamine infusions for the improvement of depressive symptoms in a population of voluntary inpatients with a DSM-5 diagnosis of a major depressive episode (MDE)	
Estimand	Mean group difference between arms in MADRS score in inpatients with MDE 24 hours after the final treatment infusion
Treatment	IMPs are biweekly ketamine (0.5 mg/kg of body weight) IV infusions or midazolam (0.045 mg/kg of body weight) IV infusions for up to eight sessions adjunctive to routine care

**ESTIMAND**	**ANALYSIS**
**Target population**<br>Patients hospitalised for moderate-to-severe depression	**Analysis set**<br>Intention-to-treat: all participants who meet trial eligibility criteria and are randomised to treatment or control condition
**Variable**<br>Depression symptom score after the end of the course of infusions	**Outcome measure**<br>MADRS at final visit: measured 24 hours after the final infusion, within the protocol-defined window of ±1h.
**Handling of intercurrent events**<br>• Withdrawal from the study without an endpoint measurement will be treated as a missing value	**Handling of missing data**<br>Withdrawal will be treated pragmatically by analysis of the last MADRS taken, according to randomised group.<br>Patients missing baseline or final MADRS will be excluded from the analysis (complete case approach). In excess of 5% missing data at the endpoint, multiple imputation will be performed to recover information.<br>Sensitivity analysis:<br>a) Truncated or skewed MADRS distribution<br>b) Missing endpoint data (tipping point analysis)<br>c) Treatment * baseline interaction effect
**Population-level summary measure**<br>Mean difference between arms in MADRS at 24h after the final infusion, adjusted for baseline MADRS	**Analysis approach**<br>ANCOVA: Linear model using final MADRS as the dependent variable, and fixed covariates for trial arm and baseline MADRS. The primary outcome will be estimated by the coefficient of a dummy variable for trial arm.

The primary analysis will be conducted once at end-of-trial by a statistician blinded to group labels. To produce this, a general linear model will be fitted to the MADRS scores, with trial arm and baseline MADRS as covariates, and using heteroscedasticity-consistent “sandwich” variance-covariance estimators (Long & Ervin, 2000). The dependent variable will be the MADRS scores 24 hours after the final infusion, including where the full course of infusions was not completed.

The efficacy of the treatment will be evaluated by way of a statistical test of the coefficient for trial arm, the p-value for which will be compared to 0.05. The coefficient itself will be presented as the effect size, corresponding to an adjusted MADRS score mean difference between arms after the final infusion, and supplemented by a 95% confidence interval. For a single primary outcome, no adjustment to the type I error is needed.

3.3 Further analyses

3.3.1 Sensitivity analysis of primary endpoint

Three assumptions underlying the analysis of covariance (ANCOVA) approach to the analysis of the primary endpoint can be examined as sensitivity analyses:

1) The assumption of Normality will be assessed by whether data drawn from a Normal distribution, with the observed parameter estimates assumed to be true values, yields the same result as that observed for the assessment of the primary endpoint. This will establish whether the findings are sensitive to truncation, skew, or other features of the MADRS measurements.
2) In the event of > 5% missing MADRS assessments at the endpoint of primary interest, a “tipping point” analysis will be conducted, imputing the missing data for a realistic worst case scenario of outcomes Missing Not At Random (MNAR). Whether the conclusion on the primary analysis shows the same conclusion will demonstrate the sensitivity of the primary analysis to bias in missing outcomes.
3) Whether there is effect modification of the difference between arms by the baseline MADRS. This will be assessed by including a baseline*arm interaction term into the primary analysis model, and examining two linear contrasts of trial arm: at high and low points on the baseline MADRS scale.

3.2.2 Analysis of safety

Safety analyses will be presented as descriptive statistics of the rates per patient and over time of adverse and serious adverse events. No statistical hypothesis testing will be performed.

3.2.3 Analysis of secondary endpoints

No formal subgroup analyses are planned for the primary outcome, as low power to detect interactions with sub-grouping factors is expected. Binary secondary outcomes of response, remission, and relapse will be summarised per treatment arm and descriptively presented.

Similar generalised linear models to the primary outcome analysis will be used to describe continuous secondary outcomes (i.e. Quick Inventory of Depressive Symptoms, Self-Report (QIDS-SR_{16}) (Rush et al., 2003) and the Montreal Cognitive Assessment (MoCA) (Nasreddine et al., 2005)). As these secondary outcomes were not powered by design, we will avoid claims of statistical significance and focus on interpretation of effect sizes and confidence intervals.

Non-continuous secondary outcomes relating to safety and tolerability will be presented descriptively; no formal analyses are planned due to anticipated low power.

4. References

Benjamini, Y., & Hochberg, Y. (1995). Controlling the False Discovery Rate: A Practical and Powerful Approach to Multiple Testing. *Journal of the Royal Statistical Society: Series B (Methodological)*, *57*(1), 289-300. https://doi.org/https://doi.org/10.1111/j.2517-6161.1995.tb02031.x

Dean, R. L., Hurducas, C., Hawton, K., Spyridi, S., Cowen, P. J., Hollingsworth, S., Marquardt, T., Barnes, A., Smith, R., McShane, R., Turner, E. H., & Cipriani, A. (2021). Ketamine and other glutamate receptor modulators for depression in adults with unipolar major depressive disorder. *Cochrane Database Syst Rev*, *9*(9), CD011612. https://doi.org/10.1002/14651858.CD011612.pub3

Dean, R. L., Marquardt, T., Hurducas, C., Spyridi, S., Barnes, A., Smith, R., Cowen, P. J., McShane, R., Hawton, K., Malhi, G. S., & et al. (2021). Ketamine and other glutamate receptor modulators for depression in adults with bipolar disorder. *Cochrane Database of Systematic Reviews*(10). https://doi.org/10.1002/14651858.CD011611.pub3

European Medicines Agency. (2020). *ICH E9 (R1) addendum on estimands and sensitivity analysis in clinical trials to the guidelines on statistical principles for clinical trials. EMA/CHMP/ICH/436221/2017.* Retrieved July 13 from https://www.ema.europa.eu/en/documents/scientific-guideline/ich-e9-r1-addendum-estimands-sensitivity-analysis-clinical-trials-guideline-statistical-principles_en.pdf

Han, Y., Chen, J. J., Zou, D. Z., Zheng, P., Li, Q., Wang, H. Y., Li, P. F., Zhou, X. Y., Zhang, Y. Q., Liu, Y. Y., & Xie, P. (2016). Efficacy of ketamine in the rapid treatment of major depressive disorder: a meta-analysis of randomized, double-blind, placebo-controlled studies. *Neuropsychiatric Disease and Treatment*, *12*, 2859-2867. https://doi.org/10.2147/ndt.S117146

Hasin, D. S., Sarvet, A. L., Meyers, J. L., Saha, T. D., Ruan, W. J., Stohl, M., & Grant, B. F. (2018). Epidemiology of Adult DSM-5 Major Depressive Disorder and Its Specifiers in the United States. *JAMA Psychiatry*, *75*(4), 336-346. https://doi.org/10.1001/jamapsychiatry.2017.4602

Jelovac, A., McCaffrey, C., Terao, M., Shanahan, E., Mohamed, E., Whooley, E., McDonagh, K., McDonogh, S., Igoe, A., Loughran, O., Shackleton, E., O'Neill, C., & McLoughlin, D. M. (2023). Study protocol for Ketamine as an adjunctive therapy for major depression (2): a randomised controlled trial (KARMA-Dep [2]). *BMC Psychiatry, 23*(1), 850. https://doi.org/10.1186/s12888-023-05365-9

Kang, M., Kendall, M. A., Ribaudo, H., Tierney, C., Zheng, L., Smeaton, L., & Lindsey, J. C. (2022). Incorporating estimands into clinical trial statistical analysis plans. *Clinical Trials*, *19*(3), 285-291. https://doi.org/10.1177/17407745221080463

Kishimoto, T., Chawla, J. M., Hagi, K., Zarate, C. A., Kane, J. M., Bauer, M., & Correll, C. U. (2016). Single-dose infusion ketamine and non-ketamine N-methyl-D-aspartate receptor antagonists for unipolar and bipolar depression: a meta-analysis of efficacy, safety and time trajectories. *Psychological Medicine*, *46*(7), 1459-1472. https://doi.org/10.1017/s0033291716000064

McIntyre, R. S., Rosenblat, J. D., Nemeroff, C. B., Sanacora, G., Murrough, J. W., Berk, M., Brietzke, E., Dodd, S., Gorwood, P., Ho, R., Iosifescu, D. V., Lopez Jaramillo, C., Kasper, S., Kratiuk, K., Lee, J. G., Lee, Y., Lui, L. M. W., Mansur, R. B., Papakostas, G. I., . . . Stahl, S. (2021). Synthesizing the Evidence for Ketamine and Esketamine in Treatment-Resistant Depression: An International Expert Opinion on the Available Evidence and Implementation. *Am J Psychiatry*, *178*(5), 383-399. https://doi.org/10.1176/appi.ajp.2020.20081251

Nasreddine, Z. S., Phillips, N. A., Bedirian, V., Charbonneau, S., Whitehead, V., Collin, I., Cummings, J. L., & Chertkow, H. (2005). The Montreal Cognitive Assessment, MoCA: a brief screening tool for mild cognitive impairment. *J Am Geriatr Soc*, *53*(4), 695-699. https://doi.org/10.1111/j.1532-5415.2005.53221.x

Price, R. B., Kissel, N., Baumeister, A., Rohac, R., Woody, M. L., Ballard, E. D., Zarate, C. A., Jr., Deakin, W., Abdallah, C. G., Feder, A., Charney, D. S., Grunebaum, M. F., Mann, J. J., Mathew, S. J., Gallagher, B., McLoughlin, D. M., Murrough, J. W., Muthukumaraswamy, S., McMillan, R., . . . Wallace, M. L. (2022). International pooled patient-level meta-analysis of ketamine infusion for depression: In search of clinical moderators. *Mol Psychiatry*. https://doi.org/10.1038/s41380-022-01757-7

Rush, A. J., Trivedi, M. H., Ibrahim, H. M., Carmody, T. J., Arnow, B., Klein, D. N., Markowitz, J. C., Ninan, P. T., Kornstein, S., Manber, R., Thase, M. E., Kocsis, J. H., & Keller, M. B. (2003). The 16-Item Quick Inventory of Depressive Symptomatology (QIDS), clinician rating (QIDS-C), and self-report (QIDS-SR): a psychometric evaluation in patients with chronic major depression. *Biol Psychiatry*, *54*(5), 573-583. https://doi.org/10.1016/s0006-3223(02)01866-8

Rush, A. J., Trivedi, M. H., Wisniewski, S. R., Nierenberg, A. A., Stewart, J. W., Warden, D., Niederehe, G., Thase, M. E., Lavori, P. W., Lebowitz, B. D., McGrath, P. J., Rosenbaum, J. F., Sackeim, H. A., Kupfer, D. J., Luther, J., & Fava, M. (2006). Acute and longer-term outcomes in depressed outpatients requiring one or several treatment steps: A STAR*D report. *American Journal of Psychiatry*, *163*(11), 1905-1917. ISI:000241669900014

Sanacora, G., Frye, M. A., McDonald, W., Mathew, S. J., Turner, M. S., Schatzberg, A. F., Summergrad, P., Nemeroff, C. B., & Novel, A. P. A. C. R. T. F. (2017). A Consensus Statement on the Use of Ketamine in the Treatment of Mood Disorders. *Jama Psychiatry*, *74*(4), 399-405. https://doi.org/10.1001/jamapsychiatry.2017.0080

Singh, J. B., Fedgchin, M., Daly, E. J., De Boer, P., Cooper, K., Lim, P., Pinter, C., Murrough, J. W., Sanacora, G., Shelton, R. C., Kurian, B., Winokur, A., Fava, M., Manji, H., Drevets, W. C., & Van Nueten, L. (2016). A Double-Blind, Randomized, Placebo-Controlled, Dose-Frequency Study of Intravenous Ketamine in Patients With Treatment-Resistant Depression. *Am J*

Psychiatry, *173*(8), 816-826. https://doi.org/10.1176/appi.ajp.2016.16010037

Wilkinson, S. T., Ballard, E. D., Bloch, M. H., Mathew, S. J., Murrough, J. W., Feder, A., Sos, P., Wang, G., Zarate, C. A., & Sanacora, G. (2018). The Effect of a Single Dose of Intravenous Ketamine on Suicidal Ideation: A Systematic Review and Individual Participant Data Meta-Analysis. *American Journal of Psychiatry*, *175*(2), 150-158. https://doi.org/10.1176/appi.ajp.2017.17040472

Xu, Y., Hackett, M., Carter, G., Loo, C., Galvez, V., Glozier, N., Glue, P., Lapidus, K., McGirr, A., Somogyi, A. A., Mitchell, P. B., & Rodgers, A. (2016). Effects of Low-Dose and Very Low-Dose Ketamine among Patients with Major Depression: a Systematic Review and Meta-Analysis. *International Journal of Neuropsychopharmacology*, *19*(4), Article pyv124. https://doi.org/10.1093/ijnp/pyv124
