# Supplementary material for: Serial Ketamine Infusions as Adjunctive Therapy to Inpatient Care for Depression: The KARMA-Dep 2 Randomized Clinical Trial
Source: JAMA Psychiatry. 2025 Oct 22;82(12):1216–24. doi: 10.1001/jamapsychiatry.2025.3019 (PMC12547681; doi:10.1001/jamapsychiatry.2025.3019)
Supplement: Supplement 2. — eMethods. Summary of exclusion criteria and cost-effectiveness statistical analysis eFigure 1. Montgomery-Åsberg Depression Rating Scale (MADRS) and 16-Item Quick Inventory of Depressive Symptoms, Self-Report (QIDS-SR-16) Outcomes Before, During and 24 Hours After Each Infusion in the Ketamine and Midazolam Groups eFigure 2. Cost-Effectiveness Plane with Seemingly Unrelated Regression Estimations (Using Observed Data) eFigure 3. Cost-Effectiveness Acceptability Curve Estimated from Seemingly Unrelated Regression (Using Observed Data) eFigure 4. Cost-Effectiveness Plane with Seemingly Unrelated Regression Estimations (Using Stacked Imputed Data) eFigure 5. Cost-Effectiveness Acceptability Curve Estimated from Seemingly Unrelated Regression (Using Stacked Imputed Data) eTable 1. Distress Levels for Symptom Groups from the Patient-Rated Inventory of Side Effects (PRISE) Across All Visits by Treatment Group eTable 2. Frequency of Individual Symptoms from the Patient-Rated Inventory of Side Effects (PRISE) Across All Visits by Treatment Group eTable 3. Unit Cost for Healthcare Services eTable 4. Assessment of Blinding After First Infusion, at the End-of-Treatment, and 24-Week Follow-Up eTable 5. Sample Sizes for Figure 2 Outcomes eReferences [file jamapsychiatry-e253019-s002.pdf]

Supplemental Online Content

Jelovac A, McCaffrey C, Terao M, et al. Serial ketamine infusions as adjunctive therapy to inpatient care for depression: the KARMA-Dep 2 randomized clinical trial. *JAMA Psychiatry*. Published online October 22, 2025. doi:10.1001/jamapsychiatry.2025.3019

eMethods. Summary of exclusion criteria and cost-effectiveness statistical analysis

eFigure 1. Montgomery-Åsberg Depression Rating Scale (MADRS) and 16-Item Quick Inventory of Depressive Symptoms, Self-Report (QIDS-SR-16) Outcomes Before, During and 24 Hours After Each Infusion in the Ketamine and Midazolam Groups

eFigure 2. Cost-Effectiveness Plane with Seemingly Unrelated Regression Estimations (Using Observed Data)

eFigure 3. Cost-Effectiveness Acceptability Curve Estimated from Seemingly Unrelated Regression (Using Observed Data)

eFigure 4. Cost-Effectiveness Plane with Seemingly Unrelated Regression Estimations (Using Stacked Imputed Data)

eFigure 5. Cost-Effectiveness Acceptability Curve Estimated from Seemingly Unrelated Regression (Using Stacked Imputed Data)

eTable 1. Distress Levels for Symptom Groups from the Patient-Rated Inventory of Side Effects (PRISE) Across All Visits by Treatment Group

eTable 2. Frequency of Individual Symptoms from the Patient-Rated Inventory of Side Effects (PRISE) Across All Visits by Treatment Group

eTable 3. Unit Cost for Healthcare Services

eTable 4. Assessment of Blinding After First Infusion, at the End-of-Treatment, and 24-Week Follow-Up

eTable 5. Sample Sizes for Figure 2 Outcomes

eReferences

© 2025 Jelovac A et al. *JAMA Psychiatry*.

This supplemental material has been provided by the authors to give readers additional information about their work.

© 2025 Jelovac A et al. *JAMA Psychiatry*.

Summary of Exclusion Criteria

Main exclusion criteria were: medical condition rendering ketamine or midazolam contraindicated; currently receiving contraindicated medications; active suicidality (score of 6 on MADRS item 10 [Suicidal Thoughts]); history of dementia, schizophrenia or schizoaffective disorder; anorexia nervosa or bulimia nervosa in the past 12 months; alcohol or other substance use disorder (except nicotine) in the previous 6 months; any DSM-5 disorder other than a major depressive episode as the primary presenting diagnosis; ECT within the last 2 months; ketamine or midazolam within the past 12 months; pregnancy, breastfeeding or inability to confirm use of adequate contraception during the trial; and inability to provide informed consent.

© 2025 Jelovac A et al. *JAMA Psychiatry*.

Cost-Effectiveness Statistical Analysis

Full economic analyses will be separately reported in a forthcoming publication. Briefly, the economic evaluation was conducted following the Health Information and Quality Authority 2025 guidelines.[1] Total costs, encompassing health service utilisation, medications, and intervention-specific expenses (acquisition and administration), were calculated for the study period from the first infusion until the 24-week follow-up.

For the analysis of cost, a micro-costing approach was adopted. Direct costs included any usage in three domains: healthcare service, medication, and the cost of carrying out the intervention. Information on health service and resource use was collected using the Client Socio-Demographic Service and Receipt Inventory, which was adapted from the Client Service Receipt Inventory and used in several previous studies of psychiatric care in the Irish healthcare system.[2-4] All over-the-counter or prescription medications and any other therapies were monitored and documented at the screening visit, and 6-, 12- and 24-week follow-ups. The intervention cost included the acquisition and administration costs of ketamine/midazolam in the intervention groups, with administration costs estimated using staff time and salary data.

To estimate the cost of resource use, we used standard national reference costs and previously published studies for the Irish setting[5-10] and supplemented these with data from the Finance Department of St. Patrick's Mental Health Services. To estimate the cost of medications, national guidelines were employed, with the price to the wholesaler extracted from the national medicine database.[11] In case of no identifiable sources, medication costs were estimated using drug tariff prices for the identical product from the British National Formulary. All the costs from sources outside of Ireland were converted to euro using purchasing power parity.[12] All costs were adjusted for inflation to 2024 price using the Consumer Price Index (www.cso.ie) and reported in euro for the year 2024. A list of unit costs and data sources for service usage is provided in **eTable 3** below.

© 2025 Jelovac A et al. *JAMA Psychiatry*.

The primary effectiveness outcome for the economic analyses was the change in the Montgomery-Åsberg Depression Rating Scale (MADRS) score from the first infusion to 24-week follow-up. To account for the potential correlation between individual-level costs and outcomes, a seemingly unrelated regression (SUR) analysis was used to estimate the incremental cost-effectiveness and the 95% CI around the mean predicted difference in cost and effect. The incremental cost-effectiveness ratio (ICER) was calculated from these regression outputs. To further explore the uncertainty surrounding the ICER, the SUR results were validated using non-parametric bootstrapping based on 1,000 resamples from the original data. The resulting cost and effect pairs were plotted on a cost-effectiveness plane to visualise the sampling uncertainty.

Where MADRS scores were missing at 24 weeks (for 4 observations in the midazolam group and 12 observations in the ketamine group), multiple imputation using chained equations was used for imputing missing MADRS scores by predictive mean matching. Five imputations were performed using baseline and the nearest available MADRS score since the last infusion as predictors.[13]

The EQ-5D-5L analysis was conducted using utility values estimated based on the Irish value set.[14] Multiple imputation by chained equations was conducted to impute missing EQ-5D-5L scores. Predictive mean matching with k = 3 nearest neighbours was used, based on baseline EQ-5D-5L score, the nearest score from 6- and 12-week follow-ups, and the corresponding MADRS scores.

© 2025 Jelovac A et al. *JAMA Psychiatry*.

eFigure 1. Montgomery-Åsberg Depression Rating Scale (MADRS) and 16-Item Quick Inventory of Depressive Symptoms, Self-Report (QIDS-SR-16) Outcomes Before, During and 24 Hours After Each Infusion in the Ketamine and Midazolam Groups

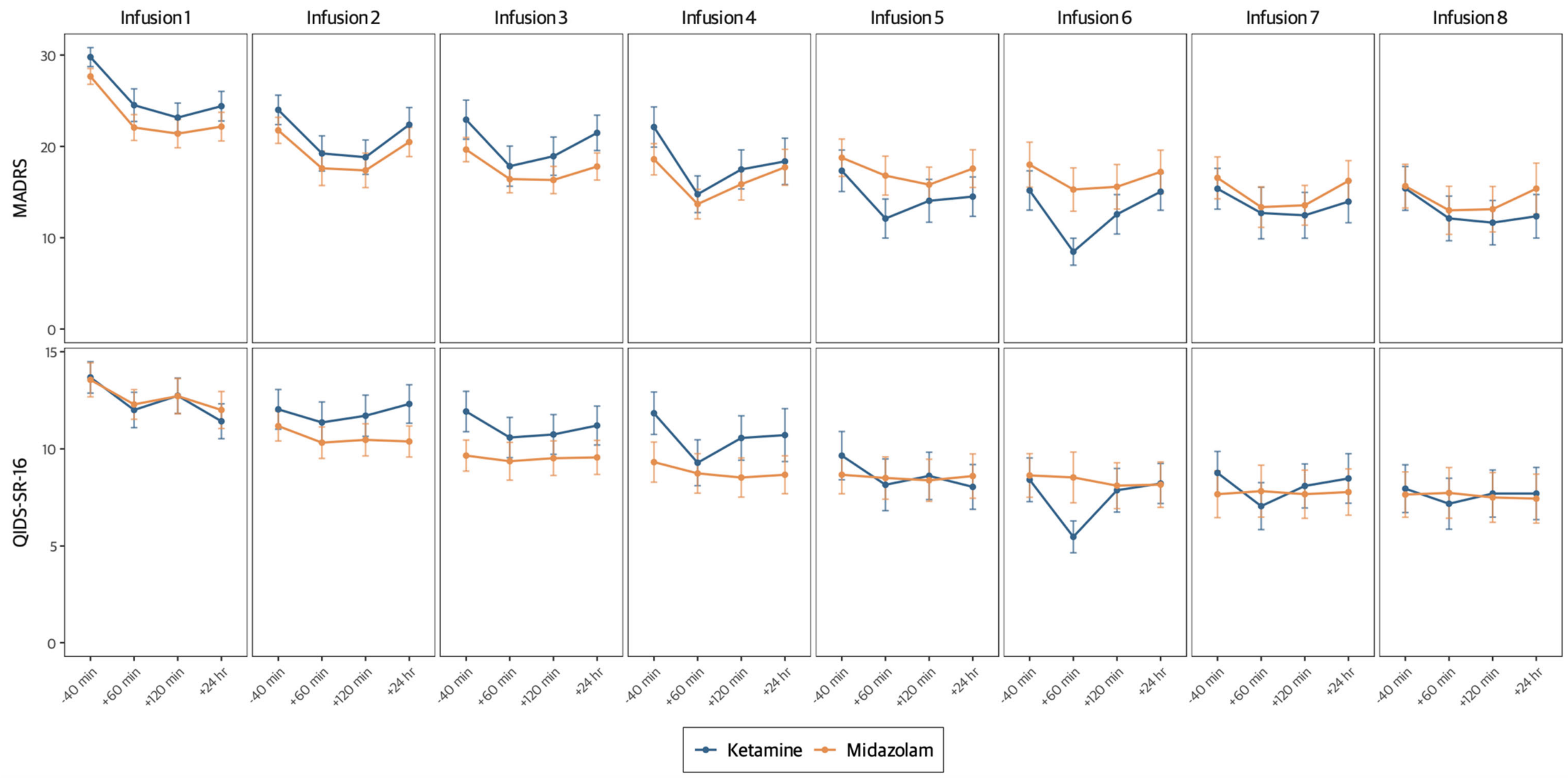

eFigure 2. Cost-Effectiveness Plane with Seemingly Unrelated Regression Estimations (Using Observed Data)

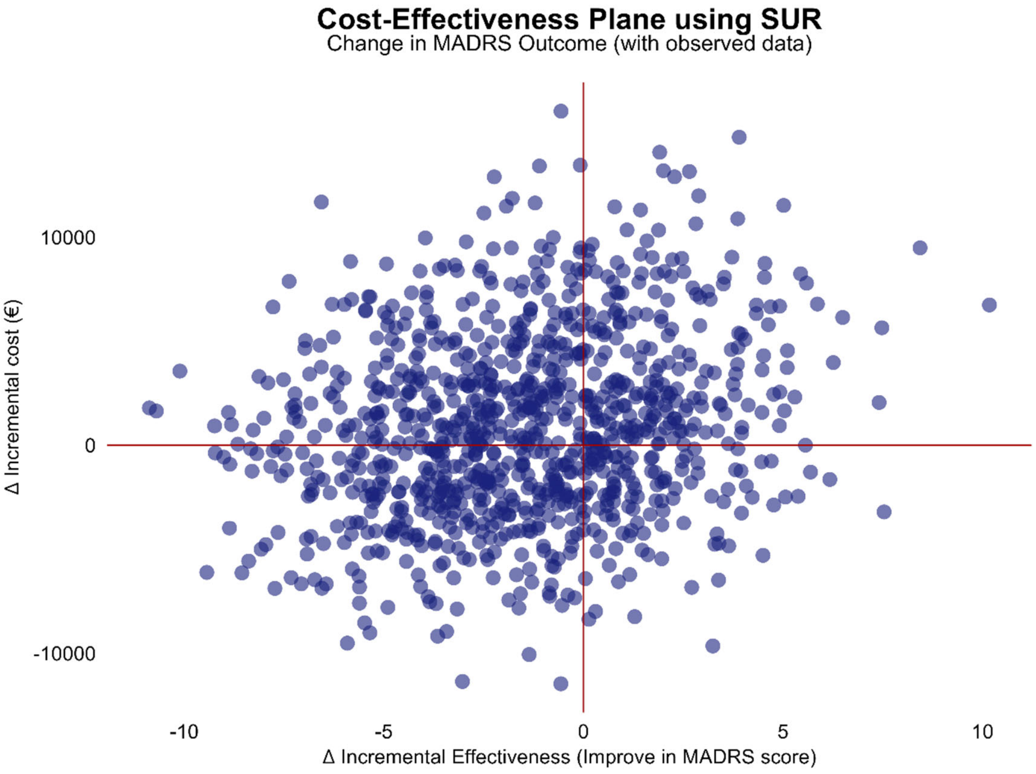

eFigure 3. Cost-Effectiveness Acceptability Curve Estimated from Seemingly Unrelated Regression (Using Observed Data)

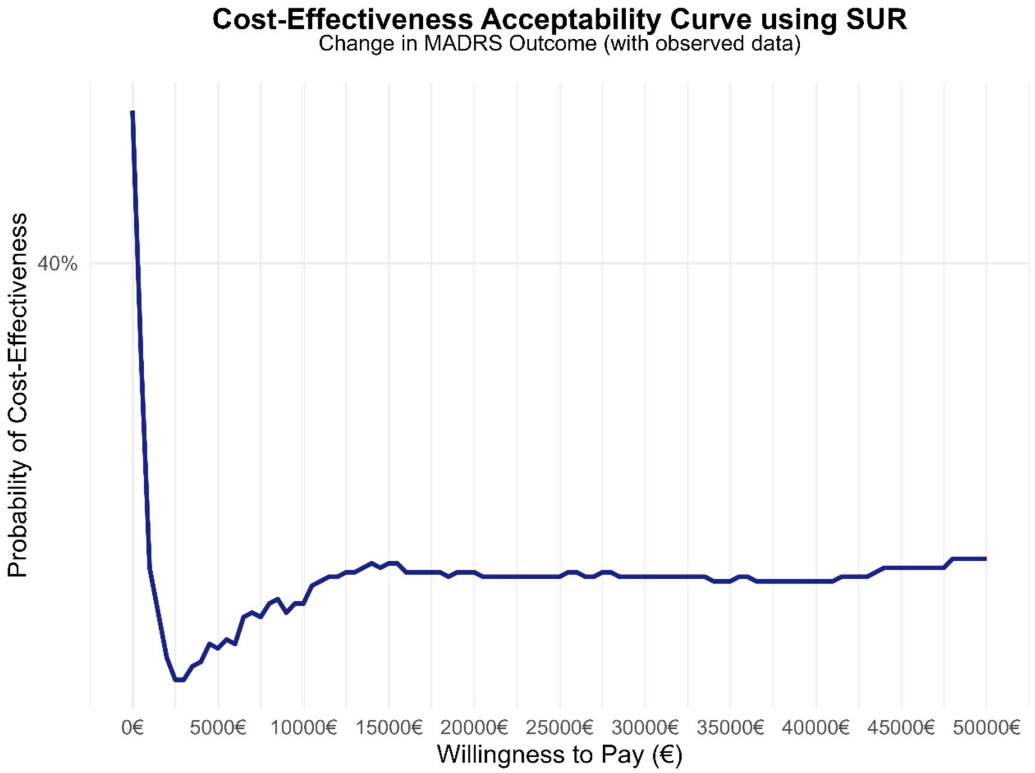

eFigure 4. Cost-Effectiveness Plane with Seemingly Unrelated Regression Estimations (Using Stacked Imputed Data)

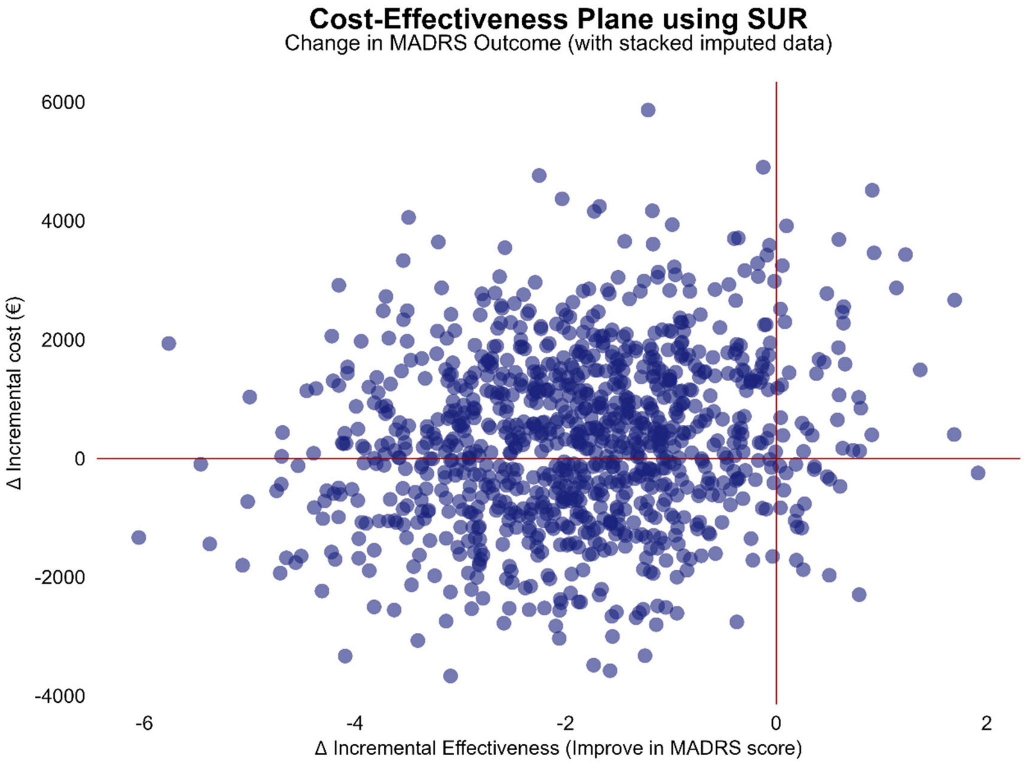

eFigure 5. Cost-Effectiveness Acceptability Curve Estimated from Seemingly Unrelated Regression (Using Stacked Imputed Data)

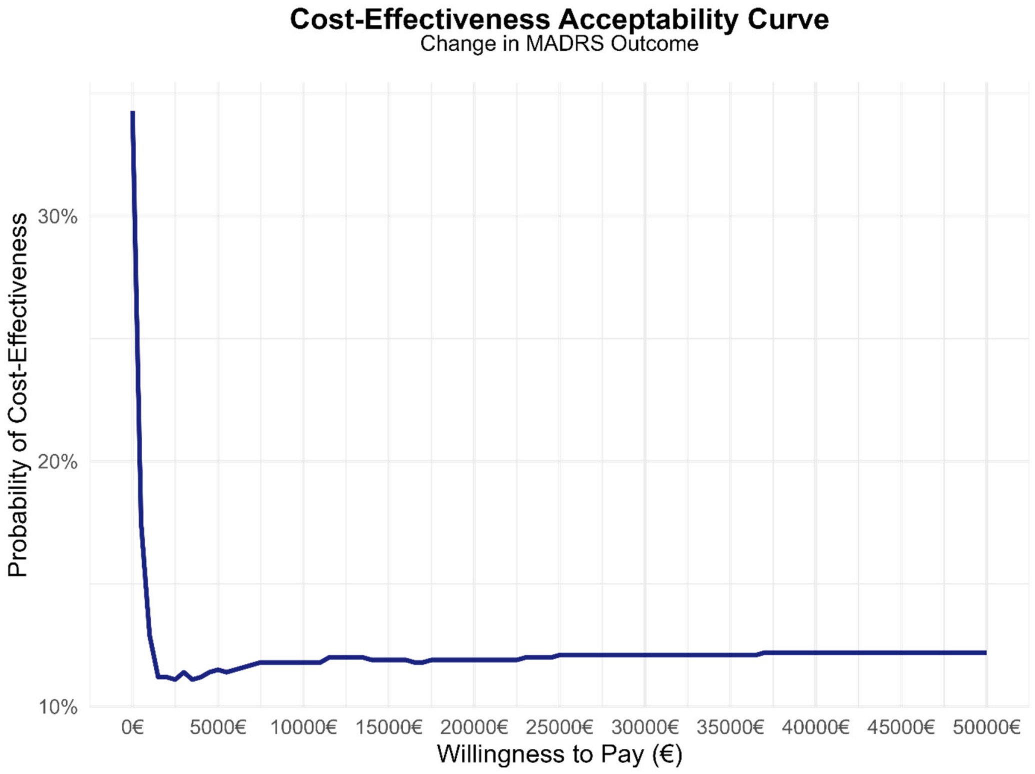

eTable 1. Distress Levels for Symptom Groups from the Patient-Rated Inventory of Side Effects (PRISE) Across All Visits by Treatment Group

	Visit 0 (Screening)		Visit 1		Visit 2		Visit 3		Visit 4		Visit 5		Visit 6		Visit 7		Visit 8		6 wk Follow-Up		12 wk Follow-Up		24 wk Follow-Up	
**Symptom Group**	**K** N = 32	**M** N = 30	**K** N = 32	**M** N = 30	**K** N = 28	**M** N = 29	**K** N = 27	**M** N = 25	**K** N = 25	**M** N = 21	**K** N = 24	**M** N = 21	**K** N = 23	**M** N = 19	**K** N = 22	**M** N = 18	**K** N = 20	**M** N = 16	**K** N = 26	**M** N = 26	**K** N = 25	**M** N = 25	**K** N = 20	**M** N = 26
**Gastrointestinal**																								
None	9 (28.1%)	10 (33.3%)	12 (37.5%)	12 (40.0%)	15 (53.6%)	17 (58.6%)	11 (42.3%)	9 (36.0%)	8 (32.0%)	10 (47.6%)	11 (45.8%)	10 (47.6%)	11 (47.8%)	11 (57.9%)	9 (40.9%)	8 (44.4%)	10 (50.0%)	7 (43.8%)	11 (42.3%)	13 (50.0%)	9 (36.0%)	15 (60.0%)	7 (35.0%)	13 (50.0%)
Tolerable	17 (53.1%)	19 (63.3%)	18 (56.3%)	18 (60.0%)	9 (32.1%)	12 (41.4%)	13 (50.0%)	15 (60.0%)	14 (56.0%)	8 (38.1%)	10 (41.7%)	9 (42.9%)	9 (39.1%)	7 (36.8%)	12 (54.5%)	8 (44.4%)	7 (35.0%)	8 (50.0%)	13 (50.0%)	13 (50.0%)	13 (52.0%)	10 (40.0%)	10 (50.0%)	12 (46.2%)
Distressing	6 (18.8%)	1 (3.3%)	2 (6.3%)	0 (0.0%)	4 (14.3%)	0 (0.0%)	2 (7.7%)	1 (4.0%)	3 (12.0%)	3 (14.3%)	3 (12.5%)	2 (9.5%)	3 (13.0%)	1 (5.3%)	1 (4.5%)	2 (11.1%)	3 (15.0%)	1 (6.3%)	2 (7.7%)	0 (0.0%)	3 (12.0%)	0 (0.0%)	3 (15.0%)	1 (3.8%)
**Heart**																								
None	23 (71.9%)	19 (63.3%)	26 (81.3%)	25 (83.3%)	21 (75.0%)	22 (75.9%)	22 (81.5%)	20 (80.0%)	19 (76.0%)	13 (61.9%)	21 (87.5%)	14 (66.7%)	19 (82.6%)	14 (73.7%)	17 (77.3%)	11 (61.1%)	17 (85.0%)	9 (56.3%)	19 (73.1%)	19 (73.1%)	18 (72.0%)	19 (76.0%)	13 (65.0%)	23 (88.5%)
Tolerable	8 (25.0%)	11 (36.7%)	5 (15.6%)	5 (16.7%)	5 (17.9%)	6 (20.7%)	3 (11.1%)	4 (16.0%)	5 (20.0%)	8 (38.1%)	3 (12.5%)	7 (33.3%)	4 (17.4%)	5 (26.3%)	5 (22.7%)	6 (33.3%)	3 (15.0%)	7 (43.8%)	7 (26.9%)	5 (19.2%)	7 (28.0%)	5 (20.0%)	7 (35.0%)	2 (7.7%)
Distressing	1 (3.1%)	0 (0.0%)	1 (3.1%)	0 (0.0%)	2 (7.1%)	1 (3.4%)	2 (7.4%)	1 (4.0%)	1 (4.0%)	0 (0.0%)	0 (0.0%)	0 (0.0%)	0 (0.0%)	0 (0.0%)	0 (0.0%)	1 (5.6%)	0 (0.0%)	0 (0.0%)	0 (0.0%)	2 (7.7%)	0 (0.0%)	1 (4.0%)	0 (0.0%)	1 (3.8%)
**Skin**																								
None	21 (65.6%)	19 (63.3%)	25 (78.1%)	21 (70.0%)	20 (71.4%)	19 (65.5%)	20 (74.1%)	17 (68.0%)	15 (60.0%)	15 (71.4%)	14 (58.3%)	15 (71.4%)	12 (52.2%)	15 (78.9%)	12 (54.5%)	15 (83.3%)	10 (50.0%)	11 (68.8%)	14 (53.8%)	19 (73.1%)	15 (60.0%)	18 (72.0%)	13 (65.0%)	17 (65.4%)
Tolerable	8 (25.0%)	7 (23.3%)	6 (18.8%)	7 (23.3%)	7 (25.0%)	8 (27.6%)	7 (25.9%)	8 (32.0%)	10 (40.0%)	6 (28.6%)	9 (37.5%)	6 (28.6%)	10 (43.5%)	4 (21.1%)	10 (45.5%)	3 (16.7%)	9 (45.0%)	5 (31.3%)	12 (46.2%)	6 (23.1%)	10 (40.0%)	6 (24.0%)	6 (30.0%)	6 (23.1%)
Distressing	3 (9.4%)	4 (13.3%)	1 (3.1%)	2 (6.7%)	1 (3.6%)	2 (6.9%)	0 (0.0%)	0 (0.0%)	0 (0.0%)	0 (0.0%)	1 (4.2%)	0 (0.0%)	1 (4.3%)	0 (0.0%)	0 (0.0%)	0 (0.0%)	1 (5.0%)	0 (0.0%)	0 (0.0%)	1 (3.8%)	0 (0.0%)	1 (4.0%)	1 (5.0%)	3 (11.5%)
**Nervous System**																								
None	13 (40.6%)	10 (33.3%)	15 (46.9%)	12 (40.0%)	13 (46.4%)	10 (34.5%)	14 (51.9%)	11 (44.0%)	10 (40.0%)	11 (52.4%)	13 (54.2%)	11 (52.4%)	13 (56.5%)	12 (63.2%)	12 (54.5%)	11 (61.1%)	13 (65.0%)	10 (62.5%)	14 (53.8%)	15 (57.7%)	17 (68.0%)	15 (60.0%)	13 (65.0%)	17 (65.4%)
Tolerable	16 (50.0%)	15 (50.0%)	14 (43.8%)	16 (53.3%)	13 (46.4%)	18 (62.1%)	11 (40.7%)	13 (52.0%)	12 (48.0%)	9 (42.9%)	8 (33.3%)	10 (47.6%)	7 (30.4%)	7 (36.8%)	9 (40.9%)	7 (38.9%)	5 (25.0%)	6 (37.5%)	9 (34.6%)	9 (34.6%)	7 (28.0%)	8 (32.0%)	4 (20.0%)	9 (34.6%)
Distressing	3 (9.4%)	5 (16.7%)	3 (9.4%)	2 (6.7%)	2 (7.1%)	1 (3.4%)	2 (7.4%)	1 (4.0%)	3 (12.0%)	1 (4.8%)	3 (12.5%)	0 (0.0%)	3 (13.0%)	0 (0.0%)	1 (4.5%)	0 (0.0%)	2 (10.0%)	0 (0.0%)	3 (11.5%)	2 (7.7%)	1 (4.0%)	2 (8.0%)	3 (15.0%)	0 (0.0%)
**Eyes/Ears**																								
None	26 (81.3%)	23 (76.7%)	26 (81.3%)	23 (76.7%)	24 (85.7%)	24 (82.8%)	22 (81.5%)	18 (72.0%)	20 (80.0%)	17 (81.0%)	19 (79.2%)	16 (76.2%)	19 (82.6%)	14 (73.7%)	18 (81.8%)	12 (66.7%)	16 (80.0%)	11 (68.8%)	22 (84.6%)	21 (80.8%)	19 (76.0%)	19 (76.0%)	17 (85.0%)	23 (88.5%)
Tolerable	4 (12.5%)	6 (20.0%)	5 (15.6%)	6 (20.0%)	3 (10.7%)	3 (10.3%)	3 (11.1%)	7 (28.0%)	4 (16.0%)	4 (19.0%)	4 (16.7%)	5 (23.8%)	4 (17.4%)	4 (21.1%)	3 (13.6%)	5 (27.8%)	4 (20.0%)	5 (31.3%)	3 (11.5%)	5 (19.2%)	6 (24.0%)	5 (20.0%)	3 (15.0%)	3 (11.5%)
Distressing	2 (6.3%)	1 (3.3%)	1 (3.1%)	1 (3.3%)	1 (3.6%)	2 (6.9%)	2 (7.4%)	0 (0.0%)	1 (4.0%)	0 (0.0%)	1 (4.2%)	0 (0.0%)	0 (0.0%)	1 (5.3%)	1 (4.5%)	1 (5.6%)	0 (0.0%)	0 (0.0%)	1 (3.8%)	0 (0.0%)	0 (0.0%)	1 (4.0%)	0 (0.0%)	0 (0.0%)
**Genital/Urinary**																								
None	21 (65.6%)	20 (66.7%)	27 (84.4%)	18 (60.0%)	22 (78.6%)	20 (69.0%)	20 (74.1%)	20 (80.0%)	19 (76.0%)	17 (81.0%)	19 (79.2%)	19 (90.5%)	18 (78.3%)	17 (89.5%)	17 (81.0%)	16 (88.9%)	14 (70.0%)	15 (93.8%)	22 (84.6%)	23 (88.5%)	21 (84.0%)	22 (88.0%)	14 (70.0%)	26 (100.0%)
Tolerable	9 (28.1%)	10 (33.3%)	5 (15.6%)	12 (40.0%)	4 (14.3%)	8 (27.6%)	7 (25.9%)	4 (16.0%)	6 (24.0%)	3 (14.3%)	4 (16.7%)	2 (9.5%)	5 (21.7%)	2 (10.5%)	4 (19.0%)	2 (11.1%)	5 (25.0%)	1 (6.3%)	4 (15.4%)	2 (7.7%)	2 (8.0%)	3 (12.0%)	4 (20.0%)	0 (0.0%)
Distressing	2 (6.3%)	0 (0.0%)	0 (0.0%)	0 (0.0%)	2 (7.1%)	1 (3.4%)	0 (0.0%)	1 (4.0%)	0 (0.0%)	1 (4.8%)	1 (4.2%)	0 (0.0%)	0 (0.0%)	0 (0.0%)	0 (0.0%)	0 (0.0%)	1 (5.0%)	0 (0.0%)	0 (0.0%)	1 (3.8%)	2 (8.0%)	0 (0.0%)	2 (10.0%)	0 (0.0%)
**Sleep**																								
None	3 (9.4%)	9 (30.0%)	11 (34.4%)	10 (34.5%)	8 (28.6%)	7 (24.1%)	11 (40.7%)	7 (28.0%)	11 (44.0%)	7 (33.3%)	9 (37.5%)	6 (28.6%)	12 (52.2%)	7 (36.8%)	11 (50.0%)	8 (44.4%)	11 (55.0%)	7 (43.8%)	16 (61.5%)	14 (53.8%)	13 (52.0%)	15 (60.0%)	11 (55.0%)	18 (69.2%)

	Visit 0 (Screening)		Visit 1		Visit 2		Visit 3		Visit 4		Visit 5		Visit 6		Visit 7		Visit 8		6 wk Follow-Up		12 wk Follow-Up		24 wk Follow-Up	
**Symptom Group**	**K** N = 32	**M** N = 30	**K** N = 32	**M** N = 30	**K** N = 28	**M** N = 29	**K** N = 27	**M** N = 25	**K** N = 25	**M** N = 21	**K** N = 24	**M** N = 21	**K** N = 23	**M** N = 19	**K** N = 22	**M** N = 18	**K** N = 20	**M** N = 16	**K** N = 26	**M** N = 26	**K** N = 25	**M** N = 25	**K** N = 20	**M** N = 26
Tolerable	14 (43.8%)	14 (46.7%)	11 (34.4%)	14 (48.3%)	12 (42.9%)	18 (62.1%)	8 (29.6%)	15 (60.0%)	9 (36.0%)	11 (52.4%)	9 (37.5%)	13 (61.9%)	6 (26.1%)	11 (57.9%)	5 (22.7%)	7 (38.9%)	4 (20.0%)	6 (37.5%)	6 (23.1%)	9 (34.6%)	9 (36.0%)	8 (32.0%)	6 (30.0%)	7 (26.9%)
Distressing	15 (46.9%)	7 (23.3%)	10 (31.3%)	5 (17.2%)	8 (28.6%)	4 (13.8%)	8 (29.6%)	3 (12.0%)	5 (20.0%)	3 (14.3%)	6 (25.0%)	2 (9.5%)	5 (21.7%)	1 (5.3%)	6 (27.3%)	3 (16.7%)	5 (25.0%)	3 (18.8%)	4 (15.4%)	3 (11.5%)	3 (12.0%)	2 (8.0%)	3 (15.0%)	1 (3.8%)
**Sexual Functioning**																								
None	19 (59.4%)	14 (46.7%)	24 (75.0%)	19 (63.3%)	21 (75.0%)	22 (75.9%)	20 (76.9%)	20 (80.0%)	17 (68.0%)	14 (66.7%)	19 (79.2%)	16 (76.2%)	18 (78.3%)	12 (63.2%)	16 (72.7%)	12 (66.7%)	14 (70.0%)	12 (75.0%)	18 (69.2%)	23 (88.5%)	17 (70.8%)	24 (96.0%)	17 (85.0%)	21 (80.8%)
Tolerable	11 (34.4%)	15 (50.0%)	5 (15.6%)	10 (33.3%)	7 (25.0%)	5 (17.2%)	5 (19.2%)	4 (16.0%)	7 (28.0%)	6 (28.6%)	5 (20.8%)	5 (23.8%)	5 (21.7%)	7 (36.8%)	6 (27.3%)	6 (33.3%)	5 (25.0%)	4 (25.0%)	6 (23.1%)	2 (7.7%)	6 (25.0%)	0 (0.0%)	2 (10.0%)	4 (15.4%)
Distressing	2 (6.3%)	1 (3.3%)	3 (9.4%)	1 (3.3%)	0 (0.0%)	2 (6.9%)	1 (3.8%)	1 (4.0%)	1 (4.0%)	1 (4.8%)	0 (0.0%)	0 (0.0%)	0 (0.0%)	0 (0.0%)	0 (0.0%)	0 (0.0%)	1 (5.0%)	0 (0.0%)	2 (7.7%)	1 (3.8%)	1 (4.2%)	1 (4.0%)	1 (5.0%)	1 (3.8%)
**Other**																								
None	0 (0.0%)	1 (3.3%)	2 (6.5%)	1 (3.4%)	6 (21.4%)	0 (0.0%)	5 (18.5%)	2 (8.0%)	1 (4.0%)	1 (4.8%)	6 (25.0%)	4 (19.0%)	6 (26.1%)	2 (10.5%)	8 (36.4%)	2 (11.1%)	7 (35.0%)	4 (25.0%)	5 (19.2%)	4 (15.4%)	5 (20.0%)	5 (20.0%)	5 (25.0%)	10 (38.5%)
Tolerable	13 (40.6%)	10 (33.3%)	16 (51.6%)	18 (62.1%)	16 (57.1%)	20 (69.0%)	14 (51.9%)	17 (68.0%)	19 (76.0%)	15 (71.4%)	15 (62.5%)	13 (61.9%)	10 (43.5%)	13 (68.4%)	9 (40.9%)	12 (66.7%)	10 (50.0%)	6 (37.5%)	16 (61.5%)	14 (53.8%)	14 (56.0%)	18 (72.0%)	12 (60.0%)	15 (57.7%)
Distressing	19 (59.4%)	19 (63.3%)	13 (41.9%)	10 (34.5%)	6 (21.4%)	9 (31.0%)	8 (29.6%)	6 (24.0%)	5 (20.0%)	5 (23.8%)	3 (12.5%)	4 (19.0%)	7 (30.4%)	4 (21.1%)	5 (22.7%)	4 (22.2%)	3 (15.0%)	6 (37.5%)	5 (19.2%)	8 (30.8%)	6 (24.0%)	2 (8.0%)	3 (15.0%)	1 (3.8%)

Abbreviations: K, Ketamine; M, Midazolam

eTable 2. Frequency of Individual Symptoms from the Patient-Rated Inventory of Side Effects (PRISE) Across All Visits by Treatment Group

	Visit 0 (Screening)		Visit 1		Visit 2		Visit 3		Visit 4		Visit 5		Visit 6		Visit 7		Visit 8		6 wk Follow-Up		12 wk Follow-Up		24 wk Follow-Up	
**PRISE Item**	**K** N = 32	**M** N = 30	**K** N = 32	**M** N = 30	**K** N = 28	**M** N = 29	**K** N = 27	**M** N = 25	**K** N = 25	**M** N = 21	**K** N = 24	**M** N = 21	**K** N = 23	**M** N = 19	**K** N = 22	**M** N = 18	**K** N = 20	**M** N = 16	**K** N = 26	**M** N = 26	**K** N = 25	**M** N = 25	**K** N = 20	**M** N = 26
Diarrhea	3 (9.4%)	3 (10.0%)	4 (12.5%)	2 (6.7%)	1 (3.6%)	0 (0.0%)	4 (14.8%)	2 (8.0%)	3 (12.0%)	2 (9.5%)	3 (12.5%)	0 (0.0%)	1 (4.3%)	1 (5.3%)	2 (9.1%)	1 (5.6%)	1 (5.0%)	1 (6.3%)	3 (11.5%)	0 (0.0%)	4 (16.0%)	1 (4.0%)	2 (10.0%)	0 (0.0%)
Constipation	12 (37.5%)	7 (23.3%)	5 (15.6%)	6 (20.0%)	6 (21.4%)	6 (20.7%)	7 (25.9%)	6 (24.0%)	4 (16.0%)	3 (14.3%)	3 (12.5%)	4 (19.0%)	4 (17.4%)	4 (21.1%)	6 (27.3%)	5 (27.8%)	4 (20.0%)	5 (31.3%)	5 (19.2%)	4 (15.4%)	10 (40.0%)	3 (12.0%)	7 (35.0%)	4 (15.4%)
Dry mouth	18 (56.3%)	13 (43.3%)	17 (53.1%)	16 (53.3%)	13 (46.4%)	10 (34.5%)	10 (37.0%)	11 (44.0%)	12 (48.0%)	6 (28.6%)	11 (45.8%)	8 (38.1%)	9 (39.1%)	5 (26.3%)	12 (54.5%)	7 (38.9%)	8 (40.0%)	7 (43.8%)	11 (42.3%)	7 (26.9%)	10 (40.0%)	9 (36.0%)	9 (45.0%)	10 (38.5%)
Nausea/vomiting	5 (15.6%)	5 (16.7%)	2 (6.3%)	3 (10.0%)	2 (7.1%)	1 (3.4%)	2 (7.4%)	1 (4.0%)	4 (16.0%)	2 (9.5%)	2 (8.3%)	1 (4.8%)	2 (8.7%)	1 (5.3%)	1 (4.5%)	0 (0.0%)	1 (5.0%)	1 (6.3%)	4 (15.4%)	3 (11.5%)	3 (12.0%)	0 (0.0%)	2 (10.0%)	2 (7.7%)
Palpitation	3 (9.4%)	3 (10.0%)	3 (9.4%)	1 (3.3%)	1 (3.6%)	2 (6.9%)	2 (7.4%)	1 (4.0%)	1 (4.0%)	3 (14.3%)	1 (4.2%)	2 (9.5%)	0 (0.0%)	2 (10.5%)	0 (0.0%)	3 (16.7%)	0 (0.0%)	2 (12.5%)	1 (3.8%)	3 (11.5%)	2 (8.0%)	1 (4.0%)	2 (10.0%)	2 (7.7%)
Dizziness on standing	4 (12.5%)	7 (23.3%)	4 (12.5%)	4 (13.3%)	6 (21.4%)	5 (17.2%)	3 (11.1%)	4 (16.0%)	4 (16.0%)	5 (23.8%)	1 (4.2%)	5 (23.8%)	4 (17.4%)	3 (15.8%)	4 (18.2%)	5 (27.8%)	2 (10.0%)	4 (25.0%)	4 (15.4%)	4 (15.4%)	5 (20.0%)	4 (16.0%)	5 (25.0%)	2 (7.7%)
Chest pain	4 (12.5%)	1 (3.3%)	1 (3.1%)	0 (0.0%)	1 (3.6%)	0 (0.0%)	2 (7.4%)	0 (0.0%)	1 (4.0%)	0 (0.0%)	1 (4.2%)	0 (0.0%)	0 (0.0%)	0 (0.0%)	1 (4.5%)	0 (0.0%)	1 (5.0%)	2 (12.5%)	3 (11.5%)	2 (7.7%)	1 (4.0%)	1 (4.0%)	2 (10.0%)	1 (3.8%)
Rash	2 (6.3%)	1 (3.3%)	0 (0.0%)	0 (0.0%)	0 (0.0%)	0 (0.0%)	0 (0.0%)	1 (4.0%)	2 (8.0%)	2 (9.5%)	2 (8.3%)	0 (0.0%)	1 (4.3%)	0 (0.0%)	2 (9.1%)	0 (0.0%)	1 (5.0%)	1 (6.3%)	0 (0.0%)	1 (3.8%)	2 (8.0%)	1 (4.0%)	0 (0.0%)	2 (7.7%)
Increased perspiration	6 (18.8%)	5 (16.7%)	4 (12.5%)	4 (13.3%)	2 (7.1%)	6 (20.7%)	2 (7.4%)	4 (16.0%)	3 (12.0%)	2 (9.5%)	2 (8.3%)	3 (14.3%)	3 (13.0%)	1 (5.3%)	2 (9.1%)	1 (5.6%)	3 (15.0%)	1 (6.3%)	4 (15.4%)	4 (15.4%)	5 (20.0%)	1 (4.0%)	2 (10.0%)	4 (15.4%)
Itching	5 (15.6%)	2 (6.7%)	2 (6.3%)	4 (13.3%)	4 (14.3%)	3 (10.3%)	3 (11.1%)	2 (8.0%)	3 (12.0%)	1 (4.8%)	2 (8.3%)	2 (9.5%)	2 (8.7%)	1 (5.3%)	2 (9.1%)	1 (5.6%)	2 (10.0%)	0 (0.0%)	3 (11.5%)	1 (3.8%)	4 (16.0%)	2 (8.0%)	1 (5.0%)	5 (19.2%)
Dry skin	5 (15.6%)	4 (13.3%)	5 (15.6%)	3 (10.0%)	4 (14.3%)	5 (17.2%)	5 (18.5%)	3 (12.0%)	8 (32.0%)	2 (9.5%)	9 (37.5%)	3 (14.3%)	11 (47.8%)	3 (15.8%)	10 (45.5%)	2 (11.1%)	10 (50.0%)	3 (18.8%)	9 (34.6%)	2 (7.7%)	8 (32.0%)	3 (12.0%)	5 (25.0%)	4 (15.4%)
Headache	6 (18.8%)	5 (16.7%)	5 (15.6%)	3 (10.0%)	4 (14.3%)	6 (20.7%)	5 (18.5%)	3 (12.0%)	5 (20.0%)	2 (9.5%)	3 (12.5%)	2 (9.5%)	2 (8.7%)	2 (10.5%)	3 (13.6%)	1 (5.6%)	3 (15.0%)	1 (6.3%)	3 (11.5%)	2 (7.7%)	2 (8.0%)	2 (8.0%)	0 (0.0%)	2 (7.7%)
Tremors	8 (25.0%)	6 (20.0%)	6 (18.8%)	9 (30.0%)	2 (7.1%)	7 (24.1%)	4 (14.8%)	7 (28.0%)	7 (28.0%)	4 (19.0%)	4 (16.7%)	3 (14.3%)	3 (13.0%)	2 (10.5%)	2 (9.1%)	2 (11.1%)	3 (15.0%)	3 (18.8%)	5 (19.2%)	2 (7.7%)	3 (12.0%)	2 (8.0%)	2 (10.0%)	1 (3.8%)
Poor coordination	5 (15.6%)	4 (13.3%)	4 (12.5%)	5 (16.7%)	8 (28.6%)	7 (24.1%)	4 (14.8%)	7 (28.0%)	4 (16.0%)	5 (23.8%)	3 (12.5%)	5 (23.8%)	2 (8.7%)	3 (15.8%)	3 (13.6%)	4 (22.2%)	2 (10.0%)	3 (18.8%)	5 (19.2%)	3 (11.5%)	3 (12.0%)	3 (12.0%)	3 (15.0%)	2 (7.7%)
Dizziness	13 (40.6%)	12 (40.0%)	12 (37.5%)	9 (30.0%)	11 (39.3%)	9 (31.0%)	10 (37.0%)	8 (32.0%)	11 (44.0%)	6 (28.6%)	8 (33.3%)	5 (23.8%)	9 (39.1%)	5 (26.3%)	8 (36.4%)	4 (22.2%)	7 (35.0%)	4 (25.0%)	6 (23.1%)	8 (30.8%)	5 (20.0%)	8 (32.0%)	5 (25.0%)	7 (26.9%)
Blurred vision	3 (9.4%)	3 (10.0%)	2 (6.3%)	3 (10.0%)	3 (10.7%)	3 (10.3%)	3 (11.1%)	4 (16.0%)	3 (12.0%)	1 (4.8%)	3 (12.5%)	1 (4.8%)	3 (13.0%)	1 (5.3%)	3 (13.6%)	1 (5.6%)	3 (15.0%)	1 (6.3%)	3 (11.5%)	3 (11.5%)	4 (16.0%)	5 (20.0%)	3 (15.0%)	3 (11.5%)
Ringing in ears	4 (12.5%)	5 (16.7%)	4 (12.5%)	5 (16.7%)	1 (3.6%)	2 (6.9%)	2 (7.4%)	4 (16.0%)	2 (8.0%)	3 (14.3%)	2 (8.3%)	4 (19.0%)	1 (4.3%)	4 (21.1%)	1 (4.5%)	5 (27.8%)	1 (5.0%)	5 (31.3%)	1 (3.8%)	2 (7.7%)	2 (8.0%)	3 (12.0%)	0 (0.0%)	0 (0.0%)

	Visit 0 (Screening)		Visit 1		Visit 2		Visit 3		Visit 4		Visit 5		Visit 6		Visit 7		Visit 8		6 wk Follow-Up		12 wk Follow-Up		24 wk Follow-Up	
**PRISE Item**	**K** N = 32	**M** N = 30	**K** N = 32	**M** N = 30	**K** N = 28	**M** N = 29	**K** N = 27	**M** N = 25	**K** N = 25	**M** N = 21	**K** N = 24	**M** N = 21	**K** N = 23	**M** N = 19	**K** N = 22	**M** N = 18	**K** N = 20	**M** N = 16	**K** N = 26	**M** N = 26	**K** N = 25	**M** N = 25	**K** N = 20	**M** N = 26
Difficulty urinating	0 (0.0%)	1 (3.3%)	0 (0.0%)	0 (0.0%)	0 (0.0%)	0 (0.0%)	0 (0.0%)	0 (0.0%)	0 (0.0%)	0 (0.0%)	0 (0.0%)	0 (0.0%)	0 (0.0%)	0 (0.0%)	0 (0.0%)	0 (0.0%)	0 (0.0%)	0 (0.0%)	0 (0.0%)	0 (0.0%)	0 (0.0%)	0 (0.0%)	0 (0.0%)	0 (0.0%)
Painful urination	3 (9.4%)	2 (6.7%)	1 (3.1%)	3 (10.0%)	2 (7.1%)	3 (10.3%)	3 (11.1%)	1 (4.0%)	3 (12.0%)	1 (4.8%)	1 (4.2%)	0 (0.0%)	1 (4.3%)	1 (5.3%)	1 (4.5%)	0 (0.0%)	1 (5.0%)	0 (0.0%)	1 (3.8%)	0 (0.0%)	1 (4.0%)	0 (0.0%)	1 (5.0%)	0 (0.0%)
Frequent urination	9 (28.1%)	6 (20.0%)	4 (12.5%)	8 (26.7%)	5 (17.9%)	6 (20.7%)	4 (14.8%)	4 (16.0%)	4 (16.0%)	3 (14.3%)	4 (16.7%)	2 (9.5%)	4 (17.4%)	1 (5.3%)	4 (18.2%)	2 (11.1%)	5 (25.0%)	1 (6.3%)	2 (7.7%)	3 (11.5%)	3 (12.0%)	2 (8.0%)	4 (20.0%)	0 (0.0%)
Menstrual irregularity	0 (0.0%)	1 (3.3%)	0 (0.0%)	2 (6.7%)	0 (0.0%)	1 (3.4%)	0 (0.0%)	0 (0.0%)	0 (0.0%)	0 (0.0%)	0 (0.0%)	0 (0.0%)	0 (0.0%)	0 (0.0%)	0 (0.0%)	0 (0.0%)	0 (0.0%)	0 (0.0%)	1 (3.8%)	0 (0.0%)	1 (4.0%)	1 (4.0%)	2 (10.0%)	0 (0.0%)
Difficulty sleeping	25 (78.1%)	18 (60.0%)	18 (56.3%)	15 (50.0%)	16 (57.1%)	14 (48.3%)	15 (55.6%)	14 (56.0%)	14 (56.0%)	11 (52.4%)	14 (58.3%)	10 (47.6%)	11 (47.8%)	11 (57.9%)	10 (45.5%)	8 (44.4%)	9 (45.0%)	9 (56.3%)	6 (23.1%)	10 (38.5%)	7 (28.0%)	6 (24.0%)	7 (35.0%)	7 (26.9%)
Sleeping too much	6 (18.8%)	4 (13.3%)	5 (15.6%)	5 (16.7%)	6 (21.4%)	9 (31.0%)	4 (14.8%)	6 (24.0%)	1 (4.0%)	4 (19.0%)	1 (4.2%)	6 (28.6%)	1 (4.3%)	1 (5.3%)	1 (4.5%)	2 (11.1%)	1 (5.0%)	0 (0.0%)	5 (19.2%)	4 (15.4%)	5 (20.0%)	4 (16.0%)	3 (15.0%)	1 (3.8%)
Loss of sexual desire	2 (6.3%)	2 (6.7%)	1 (3.1%)	3 (10.0%)	1 (3.6%)	3 (10.3%)	1 (3.7%)	2 (8.0%)	1 (4.0%)	3 (14.3%)	1 (4.2%)	2 (9.5%)	1 (4.3%)	3 (15.8%)	1 (4.5%)	3 (16.7%)	1 (5.0%)	2 (12.5%)	1 (3.8%)	2 (7.7%)	1 (4.0%)	0 (0.0%)	1 (5.0%)	2 (7.7%)
Trouble achieving orgasm	3 (9.4%)	0 (0.0%)	2 (6.3%)	0 (0.0%)	1 (3.6%)	1 (3.4%)	3 (11.1%)	1 (4.0%)	2 (8.0%)	1 (4.8%)	2 (8.3%)	1 (4.8%)	2 (8.7%)	1 (5.3%)	2 (9.1%)	1 (5.6%)	2 (10.0%)	1 (6.3%)	1 (3.8%)	0 (0.0%)	1 (4.0%)	0 (0.0%)	1 (5.0%)	0 (0.0%)
Trouble with erections	12 (37.5%)	15 (50.0%)	8 (25.0%)	11 (36.7%)	7 (25.0%)	7 (24.1%)	7 (25.9%)	5 (20.0%)	8 (32.0%)	6 (28.6%)	5 (20.8%)	4 (19.0%)	4 (17.4%)	5 (26.3%)	6 (27.3%)	5 (27.8%)	6 (30.0%)	4 (25.0%)	8 (30.8%)	2 (7.7%)	6 (24.0%)	1 (4.0%)	2 (10.0%)	4 (15.4%)
Anxiety	25 (78.1%)	23 (76.7%)	24 (75.0%)	22 (73.3%)	15 (53.6%)	18 (62.1%)	16 (59.3%)	16 (64.0%)	12 (48.0%)	9 (42.9%)	12 (50.0%)	12 (57.1%)	9 (39.1%)	10 (52.6%)	9 (40.9%)	8 (44.4%)	8 (40.0%)	5 (31.3%)	9 (34.6%)	11 (42.3%)	9 (36.0%)	10 (40.0%)	6 (30.0%)	10 (38.5%)
Poor concentration	30 (93.8%)	24 (80.0%)	25 (78.1%)	23 (76.7%)	18 (64.3%)	19 (65.5%)	19 (70.4%)	15 (60.0%)	19 (76.0%)	13 (61.9%)	13 (54.2%)	11 (52.4%)	16 (69.6%)	13 (68.4%)	13 (59.1%)	11 (61.1%)	10 (50.0%)	9 (56.3%)	16 (61.5%)	17 (65.4%)	19 (76.0%)	17 (68.0%)	9 (45.0%)	8 (30.8%)
General malaise	12 (37.5%)	10 (33.3%)	12 (37.5%)	10 (33.3%)	12 (42.9%)	10 (34.5%)	10 (37.0%)	6 (24.0%)	11 (44.0%)	6 (28.6%)	8 (33.3%)	4 (19.0%)	5 (21.7%)	6 (31.6%)	6 (27.3%)	3 (16.7%)	6 (30.0%)	3 (18.8%)	4 (15.4%)	5 (19.2%)	7 (28.0%)	5 (20.0%)	3 (15.0%)	0 (0.0%)
Restlessness	19 (59.4%)	14 (46.7%)	14 (43.8%)	11 (36.7%)	11 (39.3%)	8 (27.6%)	13 (48.1%)	6 (24.0%)	7 (28.0%)	5 (23.8%)	9 (37.5%)	3 (14.3%)	8 (34.8%)	7 (36.8%)	8 (36.4%)	5 (27.8%)	7 (35.0%)	2 (12.5%)	7 (26.9%)	6 (23.1%)	5 (20.0%)	3 (12.0%)	7 (35.0%)	2 (7.7%)
Fatigue	25 (78.1%)	19 (63.3%)	19 (59.4%)	15 (50.0%)	17 (60.7%)	15 (51.7%)	15 (55.6%)	15 (60.0%)	14 (56.0%)	9 (42.9%)	15 (62.5%)	9 (42.9%)	12 (52.2%)	9 (47.4%)	10 (45.5%)	8 (44.4%)	11 (55.0%)	7 (43.8%)	13 (50.0%)	10 (38.5%)	12 (48.0%)	7 (28.0%)	6 (30.0%)	8 (30.8%)
Decreased energy	27 (84.4%)	19 (63.3%)	22 (68.8%)	18 (60.0%)	18 (64.3%)	18 (62.1%)	14 (51.9%)	13 (52.0%)	17 (68.0%)	10 (47.6%)	11 (45.8%)	11 (52.4%)	13 (56.5%)	10 (52.6%)	11 (50.0%)	9 (50.0%)	10 (50.0%)	8 (50.0%)	11 (42.3%)	11 (42.3%)	10 (40.0%)	9 (36.0%)	11 (55.0%)	12 (46.2%)
Other	1 (3.1%)	2 (6.7%)	2 (6.3%)	1 (3.3%)	0 (0.0%)	0 (0.0%)	0 (0.0%)	1 (4.0%)	2 (8.0%)	0 (0.0%)	2 (8.3%)	1 (4.8%)	2 (8.7%)	2 (10.5%)	1 (4.5%)	0 (0.0%)	1 (5.0%)	1 (6.3%)	2 (7.7%)	1 (3.8%)	3 (12.0%)	0 (0.0%)	1 (5.0%)	0 (0.0%)

Abbreviations: K, Ketamine; M, Midazolam; PRISE, Patient-Rated Inventory of Side Effects

eTable 3. Unit Cost for Healthcare Services

Services	Index year	Original price[a]	Adjusted price, 2024	Sources
**Inpatient services**				
Acute psychiatric ward	2018	€453.00	€550.77	Keegan et al 2020[10]
Psychiatric rehabilitation ward	2015	€458.30	€562.61	Appleton et al 2023[6]
Emergency / crisis center	2018	€298.00	€362.32	Keegan et al 2020[10]
General medical ward	2015	€259.55	€318.62	Appleton et al 2023[6]
Home Care Package	2006	€75.00	€97.25	National Economic Social Forum (2008)[8]
**Outpatient hospital services**				
Psychiatric outpatient visit	2004	€94.00	€131.11	O'Shea et al 2008[7]
Other hospital outpatients visit	2018	€171.00	€207.91	Keegan et al 2020[10]
Day hospital	2004	€182.00	€253.85	O'Shea et al 2008[7]
Group therapy (hospital-based)	2015	€10.01	€12.29	Appleton et al 2023[6]
**Community based day services**				
Community mental health center	2015	€44.88	€55.09	Murphy et al 2020[9]
Group therapy	2015	€10.01	€12.29	Appleton et al 2023[6]
**Other primary & community care contacts**				
Psychiatrist	2015	€51.89	€63.70	Murphy et al 2020[9]
Psychologist	2019	€99.00	€118.80	Smith et al 2020[5]
General practitioner	2019	€46.00	€55.20	Smith et al 2020[5]
District nurse	2015	€32.72	€40.17	Murphy et al 2020[9]
Community psychiatric nurse/case manager	2015	€44.88	€55.09	Murphy et al 2020[9]
Social worker	2015	€39.60	€48.61	Murphy et al 2020[9]
Occupational therapist	2015	€39.21	€48.13	Murphy et al 2020[9]
House help / care worker	2019	€33.00	€39.60	Smith et al 2020[5]

[a]Index year is the year of original price quoted from the source.

eTable 4. Assessment of Blinding After First Infusion, at the End-of-Treatment, and 24-Week Follow-Up

Visit	Assessed Group	Ketamine (n)	Midazolam (n)	χ^2	df	*P*[a]
After First Infusion	Patients	32	28	3.02	1	.082
	Raters	32	30	34.12	1	<.001
End-of-Treatment	Patients	32	30	7.84	1	.005
	Raters	32	30	43.81	1	<.001
24-Week Follow-up	Patients	20	26	8.27	1	.004
	Raters	20	26	24.77	1	<.001

[a]Pearson's chi-square test

eTable 5. Sample Sizes for Figure 2 Outcomes

Outcome Measure	Timepoint	Ketamine (n)	Midazolam (n)
MADRS	Baseline	32	30
	End-of-Treatment	32	30
	6-Week Follow-Up	26	26
	12-Week Follow-Up	25	25
	24-Week Follow-Up	20	26
QIDS-SR-16	Baseline	31	29
	End-of-Treatment	32	30
	6-Week Follow-Up	26	26
	12-Week Follow-Up	24	22
	24-Week Follow-Up	19	25
MoCA	Baseline	31	30
	After First Infusion	31	30
	End-of-Treatment	28	30
	12-Week Follow-Up	22	19
	24-Week Follow-Up	19	24
PWC-20	Baseline	32	30
	After Fourth Infusion	24	21
	End-of-Treatment	28	30
	6-Week Follow-Up	26	25
	12-Week Follow-Up	24	22

Abbreviations: MADRS, Montgomery-Åsberg Depression Rating Scale; MoCA, Montreal Cognitive Assessment; PWC-20, 20-item Physician Withdrawal Checklist; QIDS-SR-16, 16-item Quick Inventory of Depressive Symptoms, Self-Report

eReferences

1. Health Information and Quality Authority. *National Guidelines for the Economic Evaluation of Health Technologies in Ireland*. 2025. https://www.hiqa.ie/sites/default/files/2025-03/Economic-Evaluation-Guidelines.pdf

2. Behan C. Economic Evaluation of Early Intervention in Psychosis in Comparison to Treatment as Usual. University College Dublin; 2017. http://hdl.handle.net/10147/624108

3. Raftery M, Burke K, Murray N, et al. An intensive personalised support approach to treating individuals with psychosis and co-morbid mild intellectual disability. *Ir J Psychol Med*. 2017;34(2):99-109. doi:10.1017/ipm.2016.19

4. Behan C, Kennelly B, Roche E, et al. Early intervention in psychosis: health economic evaluation using the net benefit approach in a real-world setting. *Br J Psychiatry*. 2020;217(3):484-490. doi:10.1192/bjp.2019.126

5. Smith S, Jiang J, Normand C, et al. Unit costs for non-acute care in Ireland 2016-2019. *HRB Open Res*. 2021;4(39):39. doi:10.12688/hrbopenres.13256.1

6. Appleton R, Canaway A, Tuomainen H, et al. Predictors of transitioning to adult mental health services and associated costs: a cross-country comparison. *BMJ Ment Health*. 2023;26(1):e300814. doi:10.1136/bmjment-2023-300814

7. O'Shea E, Kennelly B. *The economics of mental health care in Ireland*. 2008. 2008. Accessed 2009-11-26t14:56:37z. http://hdl.handle.net/10147/86946

8. National Economic Social Forum. *The Policy Implications of Social Capital*. 2008:40-45. http://files.nesc.ie/nesf_archive/nesf_reports/NESF_38_full.pdf

9. Murphy A, Bourke J, Flynn D, et al. A cost-effectiveness analysis of dialectical behaviour therapy for treating individuals with borderline personality disorder in the community. *Ir J Med Sci*. 2020;189(2):415-423. doi:10.1007/s11845-019-02091-8

10. Keegan C, Brick A, Bergin A, et al. *Projections of Expenditure for Public Hospitals in Ireland, 2018–2035, Based on the Hippocrates Model*. 2020. https://www.esri.ie/publications/projections-of-expenditure-for-public-hospitals-in-ireland-2018-2035

11. Health Service Executive. *Welcome to Primary Care Reimbursement Service - HSE.ie*. 2017. http://www.hse.ie/eng/staff/PCRS/

12. Joint Formulary Committee. *British National Formulary*. 87 ed. BNF. BMJ Group and Pharmaceutical Press; 2024.

13. White IR, Royston P, Wood AM. Multiple imputation using chained equations: Issues and guidance for practice. *Stat Med*. 2011;30(4):377–399. https://doi.org/10.1002/sim.4067

14. Hobbins A, Barry L, Kelleher D, Shah K, Devlin N, Goni JMR, O'Neill C. Utility Values for Health States in Ireland: A Value Set for the EQ-5D-5L. *PharmacoEconomics*. 2018;36(11):1345-1353. https://doi:10.1007/s40273-018-0690-x
