# Supplementary material for: Serial Ketamine Infusions as Adjunctive Therapy to Inpatient Care for Depression: The KARMA-Dep 2 Randomized Clinical Trial
Source: JAMA Psychiatry. 2025 Oct 22;82(12):1216–24. doi: 10.1001/jamapsychiatry.2025.3019 (PMC12547681; doi:10.1001/jamapsychiatry.2025.3019)
Supplement: Supplement 3. — Data sharing statement [file jamapsychiatry-e253019-s003.pdf]

Data Sharing Statement

Jelovac. Serial Ketamine Infusions as Adjunctive Therapy to Inpatient Care for Depression. *JAMA Psychiatry*. Published October 22, 2025. doi:10.1001/jamapsychiatry.2025.3019

Data
Additional Information: ClinicalTrials.gov https://clinicaltrials.gov/study/NCT04939649 NCT04939649
Data available: No

Additional Information
Explanation for why data not available: Individual participant data cannot be shared as not all trial participants consented to data sharing.
